# Synthesis, characterisation and antibacterial activity of flavone-based Sn(iv) and Sb(iii) complexes

**DOI:** 10.1039/d6ra04588j

**Published:** 2026-06-05

**Authors:** Jakia Parbin Sultana, Sunali Saikia, Partha Pratim Saharia, Namisha Das, Debasish Borbora, Tridib K. Goswami

**Affiliations:** a Department of Chemistry, Gauhati University Guwahati 781014 Assam India tridibgoswami05@gmail.com; b Department of Biotechnology, Gauhati University Guwahati 781014 Assam India debasish.borbora@gauhati.ac.in

## Abstract

Three 3-hydroxyflavone ligands (L_1_–L_3_) and their corresponding Sn(iv) and Sb(iii) complexes (1–6) of general formula [Sn(L)_3_]Cl (1–3) and [Sb(L)_2_Cl] (4–6), where L is the monoanionic form of 3-hydroxy flavone (L_1_ in 1 and 4), naphthyl flavone (L_2_ in 2 and 5) or anthracenyl flavone (L_3_ in 3 and 6), are synthesized and characterised using UV-visible, fluorescence, IR, NMR and high-resolution mass spectral studies. The compounds were evaluated against *Staphylococcus aureus* (MTCC 96), *Escherichia coli* (MTCC 111), *Pseudomonas aeruginosa* (MTCC 1688) and *Klebsiella pneumoniae* (MTCC 432). In general, coordination of the flavone ligands to Sn(iv) and Sb(iii) enhanced antibacterial activity relative to the parent ligands and the corresponding metal salts. Among the series, the Sb(iii) complexes were the most active, with complex 6 showing the highest potency, particularly against *S. aureus*, for which the minimum inhibitory concentration (MIC) was 2.9 µg mL^−1^, comparable to ciprofloxacin under the tested conditions. The expanded MIC dataset also indicated a clear organism-dependent susceptibility pattern, with *S. aureus* being more susceptible than the tested Gram-negative bacteria. Checkerboard analysis further revealed synergistic interactions between the Sb(iii) complexes and ciprofloxacin against *S. aureus*. Mechanistic studies, including scanning electron microscopy, membrane permeability analysis and propidium iodide staining, indicated that the antibacterial action of complex 6 involves disruption and permeabilization of the bacterial membrane. In addition, the cytotoxic effects of the two most active complexes, 5 and 6, were assessed in human dermal fibroblast (HDF) cells by MTT assay. Both complexes maintained high HDF viability at their respective MICs against *S. aureus*, indicating that their anti-staphylococcal activity was achieved at concentrations that did not cause marked cytotoxicity under the tested conditions. These findings identify Sb(iii)-bound flavonoid complexes as promising scaffolds for further antibacterial investigation.

## Abstract 2

Sn(iv) and Sb(iii) flavonoid complexes display significant *in vitro* antibacterial activity, especially against the Gram-positive *S. aureus* (MTCC 96) probably *via* rupture of cell membrane as evident from scanning electron microscopic images.

## Graphical abstract

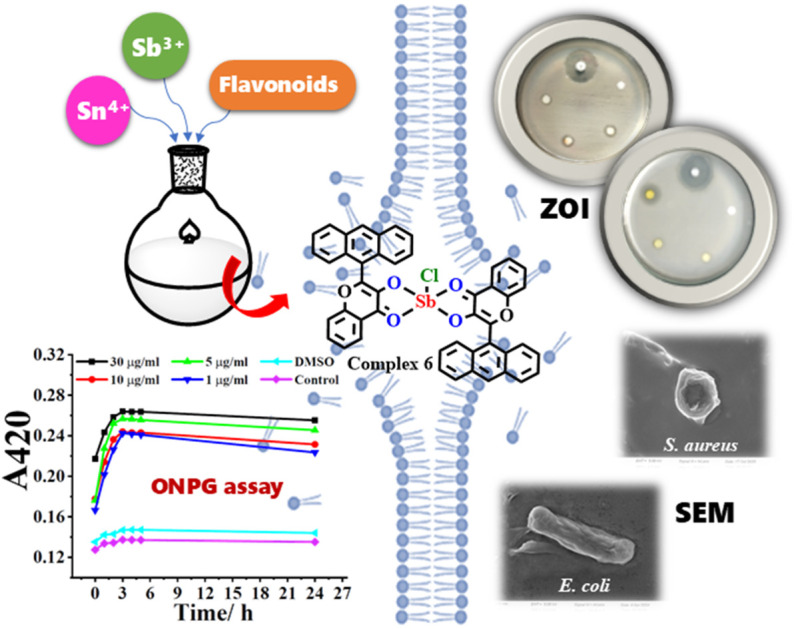

## Introduction

Antibiotics have played a central role in the treatment of bacterial infections in human and veterinary medicine, agriculture and allied sectors. However, the continued emergence of antimicrobial resistance (AMR) has reduced the effectiveness of many existing agents and has become a major global health concern.^1–7^ Multiple factors contribute to this problem, including genetic variation in bacterial pathogens, altered cell envelope properties, biofilm formation, and other resistance-associated adaptations.^4,5^ In parallel, the clinical antibacterial pipeline remains limited, particularly with respect to structurally novel agents.^8,9^ These challenges have stimulated continued interest in identifying new antibacterial scaffolds with distinct structural and physicochemical features. One such approach involves developing metal-based antimicrobial agents. Since the approval of cisplatin as an anticancer drug in 1978, metal complexes have become a fundamental component of medicinal chemistry. In the last twenty years, several ruthenium, titanium, iron, silver, gold, palladium, gallium, bismuth and copper-based metal complexes have advanced to clinical trials for the treatment of cancer, malaria, and neurodegenerative diseases.^10–16^ However, only a few studies have focused on their antimicrobial applications. The unique coordination chemistry of metal ions provides a wide variety of three-dimensional geometries, offering more opportunities to create and manage structural variation. Besides, metal complexes have been reported to act by interacting with intracellular biomolecules, inhibiting enzymes and altering the functions of bacterial cell membranes, *etc.*^17,18^ This ability to affect multiple cellular functions in bacteria slows the development of resistance.^9,17,19^

Among the various metals explored, tin and antimony stand out for their unique coordination chemistry and reported biological activity.^20–23^ Antimony has been used in medicine for a long time. The ancient Egyptians employed antimony ores and salts to treat fevers and skin irritation.^24,25^ Pentavalent antimonial complexes, such as sodium stibogluconate (Pentostam™) and meglumine antimonate (Glucantime™), have been used to treat leishmaniasis, a disease caused by the protozoan parasite *Leishmania*.^26^ Similarly, Tin complexes have shown promising antimicrobial properties, attributed to their ability to interact with biological macromolecules and disrupt microbial cellular processes.^27–29^

**Chart 1:**
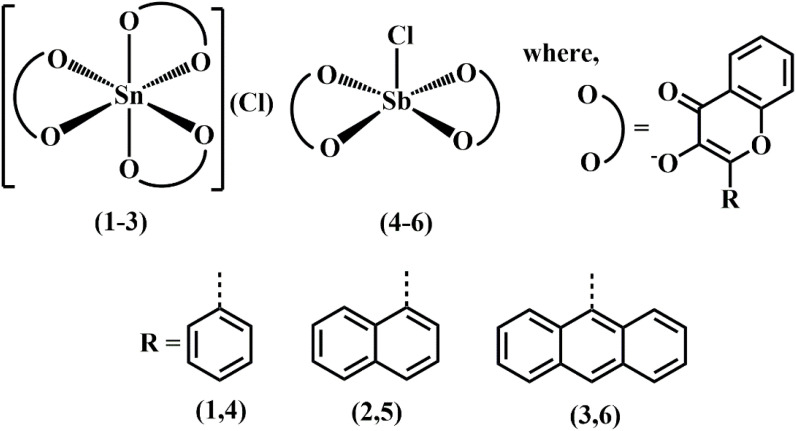
Schematic drawings of the complexes [Sn(L)_3_]Cl (1–3) and [Sb(L)_2_Cl] (4–6) where, L is 3-hydroxy flavone (L_1_) (1, 4), naphthyl flavone (L_2_) (2, 5) and anthracenyl flavone (L_3_) (3, 6) respectively.

To further enhance their biological efficacy and specificity, metal complexes are often functionalized with nature-inspired bioactive organic ligands. Flavonoids, a class of bioactive compounds, have been extensively investigated due to their intriguing medicinal properties.^30–35^ Studies suggest that, in addition to their inherent antimicrobial properties, flavonoids show increased activity when chelated to metal ions. This improvement is mainly due to increased lipophilicity, synergistic interactions and improved binding affinity to targets.^36–38^ This inspired us to design and synthesize metal complexes having flavonoids as ligands. Herein, we present the synthesis of three flavones, *viz.* 3-hydroxy-2-phenyl-4*H*-chromen-4-one (L_1_); 3-hydroxy-2-(naphthalen-2-yl)-4*H*-chromen-4-one (L_2_); 2-(anthracen-9-yl)-3-hydroxy-4*H*-chromen-4-one (L_3_) and their corresponding Sn(iv) and Sb(iii) complexes having the formulation, [Sn(L)_3_]Cl (1–3) and [Sb(L)_2_Cl] (4–6) where, L = L_1_ (in 1 and 4), L_2_ (in 2 and 5) and L_3_ (in 3 and 6) respectively (Chart 1). The *in vitro* antibacterial activity of the metal salts, ligands and synthesized complexes was evaluated against *Staphylococcus aureus* (MTCC 96), *Escherichia coli* (MTCC 111), *Pseudomonas aeruginosa* (MTCC 1688) and *Klebsiella pneumoniae* (MTCC 432) using disc diffusion and broth dilution assays. On the basis of MIC values, complex 6 emerged as the most promising candidate, particularly against Gram-positive *S. aureus*. To gain insight into its antibacterial mechanism, membrane damage induced by complex 6 was investigated using scanning electron microscopy to visualise changes in cell morphology and membrane integrity. In addition, PI staining and ONPG-based inner membrane permeabilization assays were performed to examine membrane permeability and cytoplasmic leakage in *S. aureus* and *E. coli*. The results suggest membrane disruption as a possible mechanism for antibacterial action. Cytotoxicity of the most effective complexes, 5 and 6, were tested on HDF cells using MTT assay. At the minimum inhibitory concentrations against *S. aureus*, both complexes exhibited high cell viability. This indicates that their antibacterial properties are effective at doses that do not cause significant damage to normal human cells.

## Experimental section

### Materials and measurements

All the solvents and reagents used for synthesis and biological assays were purchased from commercial sources (Loba Chemie, India; Sigma-Aldrich, U.S.A.; HiMedia, India) and used without further purification. Tris-(hydroxymethyl)aminomethane–HCl (Tris–HCl) buffer solution was prepared using deionized and sonicated double distilled water. 1-Naphthaldehyde, 9-anthracene carboxaldehyde, antimony(iii)chloride, 2-hydroxyacetophenone, ciprofloxacin and doxorubicin were purchased from Sigma-Aldrich. Tin(ii) chloride and benzaldehyde were purchased from Merck and Loba Chemie, respectively. Nutrient broth, Mueller–Hinton agar, alpha-MEM, fetal bovine serum, antibiotic antimycotic solution-penicillin & streptomycin, trypsin–EDTA solution, DPBS and antibiotic discs were obtained from HiMedia. The 3-hydroxy flavone ligands were synthesized according to a previously published literature.^39^

The elemental analysis was performed on a Thermo Finnigan Flash EA 1112 CHN analyzer. Shimadzu IR Affinity-1S, Shimadzu UV-1800, and Hitachi F-2500 spectrophotometers were used to acquire infrared, UV-visible and emission spectra, respectively. Electrospray ionization (ESI) mass spectral measurements were carried out using a Waters Xevo G2 XS QTof high-resolution mass spectrometer. ^1^H and ^13^C NMR spectra were recorded on Bruker 400 MHz NMR spectrometer at room temperature. The absorbance values in 96-well plates were recorded using a Thermo Fisher microplate reader (Multi-scan Go) and analysed using Skanit 7.0 software. The scanning electron microscopic images were obtained from Carl Zeiss ΣIGMA Scanning Electron Microscope. Fluorescence microscopic images were observed under a Zeiss Axiovert 5 inverted fluorescence microscope equipped with Axiocam 202 mono camera (ZEISS, Germany) using ZEISS ZEN 3.12 software.

### General procedure for synthesis of metal complexes (1–6)

The complexes 1–3 were synthesized by following a general procedure where the 3-hydroxyflavone ligands (1 mmol) [L = 3-hydroxyflavone (L_1_, 1) (0.24 g), naphthyl flavone (L_2_, 2) (0.29 g) and anthracenyl flavone (L_3_, 3) (0.34 g)] were dissolved in 10 mL methanol along with 1 equivalent of NaOH (1 mmol, 0.04 g). This solution was then added dropwise to a stirred solution of tin(ii) chloride dihydrate (0.5 mmol, 0.11 g) in methanol (5 mL). The reaction mixture was then stirred for 6 hours at room temperature. The complexes precipitated as yellow solids, which were then washed with diethyl ether and dried in a vacuum desiccator over anhydrous CaCl_2._ Complexes 4–6 were also synthesized following a similar synthetic procedure using Sb(iii) chloride (0.5 mmol, 0.11 g) as metal precursor [yield: 0.48 g, 75% for 1, 0.54 g, 72% for 2, 0.69 g, 79% for 3, 0.54 g, 77% for 4, 0.36 g, 71% for 5, 0.48 g, 78% for 6].

Anal. calcd for C_45_H_27_O_9_Sn (1): C, 62.42; H, 3.14; found: C, 62.19; H, 3.18. Selected IR data (KBr phase, cm^−1^): 3444br (O–H), 1609s (C=O), 1478w (C=C), 1415w, 1201w (C–O–C) (*vs.*, very strong; s, strong; sh, sharp; m, medium; w, weak; br, broad). UV-visible in DMSO–Tris–HCl buffer (pH 7.2) (1 : 4 v/v) [*λ*_max_/nm (*ε*/M^−1^ cm^−1^)]: 234(31 840), 310(18 720), 344(21 240). HRMS (ESI) in MeCN: *m*/*z* = 831.0684 [M]^+^ (calcd: 831.0677). ^1^H NMR (400 MHz, CDCl_3_): *δ*ppm, 8.30–8.28 (m, 6H), 8.24–8.22 (m, 3H), 7.76–7.72 (t, *J* = 8 Hz, 3H), 7.69–7.67 (m, 3H), 7.59–7.50 (m, 6H), 7.59–7.50 (t, *J* = 8 Hz, 3H), 7.40–7.36 (t, *J* = 8 Hz, 3H), 7.03 (s, 3H). ^13^C NMR (100 MHz, CDCl_3_): *δ*ppm, 178.723, 154.623, 134.566, 133.064, 129.448, 128.900, 128.185, 127.680, 125.717, 125.291, 124.432, 120.469, 118.160.

Anal. calcd for C_57_H_33_O_9_Sn (2): C, 67.38; H, 3.27; found: C, 67.26; H, 3.31. Selected IR data (KBr phase, cm^−1^): 3440br (O–H), 1639s (C=O), 1513w (C=C), 1415w, 1386w (C–O–C). UV-visible in DMSO–Tris–HCl buffer (pH 7.2) (1 : 4 v/v) [*λ*_max_/nm (*ε*/M^−1^ cm^−1^)]: 237(38 800), 331(24 440). HRMS (ESI) in MeCN: *m*/*z* = 981.1187 [M]^+^ (calcd: 981.1144). ^1^H NMR (400 MHz, CDCl_3_): *δ*ppm, 8.36–8.33 (dd, 3H), 8.03 (d, *J* = 4, 3H), 7.97–7.95 (m, 3H), 7.90–7.85 (m, 6H), 7.75–7.71 (m, 3H), 7.65–7.61 (m, 3H), 7.59–7.52 (m, 9H), 7.50–7.46 (m, 3H), 6.54 (s, 3H). ^13^C NMR (100 MHz, CDCl_3_): *δ*ppm, 173.158, 156.666, 145.594, 140.578, 134.154, 133.829, 131.289, 130.451, 130.127, 128.921, 127.261, 127.203, 125.760, 125.579, 125.219, 124.865, 123.457, 121.667, 118.773.

Anal. calcd for C_69_H_39_O_9_Sn (3): C, 71.06; H, 3.37; found: C, 70.90; H, 3.31. Selected IR data (KBr phase, cm^−1^): 3444br (O–H), 1609s (C=O), 1476s (C=C), 1401w, 1345w (C–O–C). UV-visible in DMSO–Tris–HCl buffer (pH 7.2) (1 : 4 v/v) [*λ*_max_/nm (*ε*/M^−1^ cm^−1^)]: 250(31 480), 264(27 920), 329(15 200), 377(13 280), 393(13 280). HRMS (ESI) in MeCN: *m*/*z* = 1131.2443 [M]^+^ (calcd: 1131.1616). ^1^H NMR (400 MHz, CDCl_3_): *δ*ppm, 8.69 (s, 3H), 8.46–8.43 (dd, 3H), 8.14–8.12 (m, 6H), 7.86–7.83 (m, 6H), 7.78–7.74 (m, 3H), 7.57–7.50 (m, 18H). ^13^C NMR (100 MHz, CDCl_3_): *δ*ppm, 193.566, 156.041, 147.098, 139.050, 133.724, 131.212, 128.780, 128.635, 127.762, 126.968, 126.347, 125.626, 125.395, 125.026, 124.702, 121.309, 118.502.

Anal. calcd for C_30_H_18_ClO_6_Sb (4): C, 57.04; H, 2.87; found: C, 56.93; H, 2.91. Selected IR data (KBr phase, cm^−1^): 3440br (O–H), 1619s (C=O), 1555s, 1496s (C=C), 1417s, 1356w, 1309w (C–O–C). UV-visible in DMSO–Tris–HCl buffer (pH 7.2) (1 : 4 v/v) [*λ*_max_/nm (*ε*/M^−1^ cm^−1^)]: 245(35 560), 312(18 840), 343(20 480). HRMS (ESI) in MeCN: *m*/*z* = 595.0128 [M–Cl]^+^ (calcd: 595.0136). ^1^H NMR (400 MHz, CDCl_3_): *δ*ppm, 8.42–8.39 (m, 2H), 8.26–8.23 (m, 4H), 7.76–7.68 (m, 4H), 7.60–7.58 (m, 2H), 7.54–7.50 (t, *J* = 8 Hz, 4H), 7.47–7.46 (m, 2H), 7.23 (s, 2H). ^13^C NMR (100 MHz, CDCl_3_): *δ*ppm, 193.746, 163.583, 145.474, 138.711, 134.590, 130.923, 129.646, 129.040, 128.657, 127.047, 126.138, 120.118, 118.134.

Anal. calcd for C_38_H_22_O_6_Sb (5): C, 62.37; H, 3.03; found: C, 62.25; H, 3.11. Selected IR data (KBr phase, cm^−1^): 3440br (O–H), 1619w, 1582s (C=O), 1535s (C=C), 1411w, 1392w (C–O–C). UV-visible in DMSO–Tris–HCl buffer (pH 7.2) (1 : 4 v/v) [*λ*_max_/nm (*ε*/M^−1^ cm^−1^)]: 236(35 200), 331(20 640). HRMS (ESI) in MeCN: *m*/*z* = 695.0419 [M–Cl]^+^ (calcd: 695.0449). ^1^H NMR (400 MHz, CDCl_3_): *δ*ppm, 8.38–8.36 (d, *J* = 8, 2H), 8.05 (d, *J* = 8.4, 2H), 7.99–7.97 (m, 2H), 7.92–7.87 (m, 4H), 7.77–7.73 (m, 2H), 7.67–7.63 (m, 2H), 7.60–7.54 (m, 6H), 7.50 (t, *J* = 7.6, 2H), 6.56 (s, 2H). ^13^C NMR (100 MHz, CDCl_3_): *δ*ppm, 173.324, 156.096, 146.973, 139.055, 133.764, 131.166, 130.603, 128.806, 128.676, 127.795, 127.009, 126.388, 125.659, 125.435, 125.074, 124.735, 121.328, 118.557.

Anal. calcd for C_46_H_26_ClO_6_Sb (6): C, 66.41; H, 3.15; found: C, 66.32; H, 3.12. Selected IR data (KBr phase, cm^−1^): 3444br (O–H), 1637s, 1584s (C=O), 1482w (C=C). UV-visible in DMSO–Tris–HCl buffer (pH 7.2) (1 : 4 v/v) [*λ*_max_/nm (*ε*/M^−1^ cm^−1^)]: 250(3040), 329(14 600), 376(13 280), 393(13 280). HRMS (ESI) in MeCN: *m*/*z* = 795.0751 [M–Cl]^+^ (calcd: 795.0762). ^1^H NMR (400 MHz, CDCl_3_): *δ*ppm, 8.69 (s, 2H), 8.44 (d, *J* = 8.4, 2H), 8.12 (d, *J* = 9.2, 4H), 7.84–7.81 (d, *J* = 7.6, 4H), 7.87–7.85 (m, 2H), 7.56–7.50 (m, 12H). ^13^C NMR (100 MHz, CDCl_3_): *δ*ppm, 193.219, 163.807, 142.688, 136.654, 133.803, 131.284, 129.884, 129.386, 128.982, 128.809, 127.105, 126.636, 125.481, 125.120, 124.846, 119.938, 118.697.

### Antibacterial activity

The ligands and their corresponding metal complexes were screened for antibacterial activity against *Staphylococcus aureus* (MTCC 96), *Escherichia coli* (MTCC 111), *Pseudomonas aeruginosa* (MTCC 1688) and *Klebsiella pneumoniae* (MTCC 432) using the disc diffusion method on Mueller–Hinton agar.^40^ The bacterial inoculum was prepared by diluting an overnight culture of bacteria grown in nutrient broth and adjusting the turbidity to 0.5 according to McFarland's standard, corresponding to approximately 1 × 10^8^ to 2 × 10^8^ CFU mL^−1^. From this, 100 µL of bacterial inoculum was taken and spread uniformly over prepared agar plates, which were allowed to dry for 3–5 minutes. Filter paper discs soaked with the standard antibiotic (ciprofloxacin, 0.2 mg mL^−1^) and various concentrations (3 mg mL^−1^, 1.5 mg mL^−1^, and 1.2 mg mL^−1^) of test compounds were carefully placed over the agar plate using sterile forceps. A disc containing DMSO alone served as the control. The plates were incubated at 37 °C for 24 hours, and the zone of inhibition was measured. The antimicrobial efficacy of the ligands and their corresponding metal complexes was assessed by measuring the diameter of inhibition zones formed around the discs. All the tests were performed in duplicate.

### Determination of MIC using resazurin-based microdilution assay

The resazurin-based broth microdilution method was used to evaluate the minimum inhibitory concentration of the ligands and their corresponding metal complexes.^41,42^ Sterile, disposable microdilution plates with 96 U-shaped wells were used. The wells containing 100 µL of nutrient broth were inoculated with the same inoculum (10 µL) used to measure the inhibition zone. Compounds dissolved in DMSO at a concentration of 3 mg mL^−1^ were added to the first well of the plate and were serially diluted up to the 16th well. After overnight incubation, 20 µL of resazurin solution (5 mg mL^−1^) was pipetted into each well to serve as a growth indicator, changing colour in the presence of viable bacteria. Plates were incubated for 2 hours at 37 °C prior to measuring the absorbance of each well using a microplate reader (Multi-scan Go, Thermo Fisher) at 595 nm.^43^ In the medium, the final concentration of DMSO was less than 1%, which had no effect on the growth of the bacterial strains. For the experiment, ciprofloxacin hydrochloride at 0.2 mg mL^−1^ was used as the reference antimicrobial agent.

### Synergy assay

On the basis of zones of inhibition and MIC values, the Sb(iii) complexes were found to be more potent than their Sn(iv) counterparts. Therefore, the synergistic effects of complexes 4, 5 and 6 in combination with the standard antibiotic ciprofloxacin were investigated using a checkerboard assay in 96-well microtiter plates.^44–47^ Nutrient broth was used as a growth medium for both Gram-positive and Gram-negative strains. The concentration of ciprofloxacin was varied, ranging from 20 µg mL^−1^ to 0.0006 µg mL^−1^, whereas for the complexes, the concentrations ranged from 1500 µg mL^−1^ to 0.0457 µg mL^−1^ 10 µL of inoculum was added to each well and incubated for 24 hours at 37 °C. After determining the MIC for each combination, the fractional inhibitory concentration (FIC) was calculated as:ΣFIC = FIC_A_ + FIC_B_ = (*C*_A_/MIC_A_) + (*C*_B_/MIC_B_)where MIC_A_ and MIC_B_ are the MICs of drugs A and B alone, respectively, and *C*_A_ and *C*_B_ are the concentrations of the drugs in combinations, respectively. FICi (fractional inhibitory concentration index) values were interpreted as follows: FICi < 0.5, synergistic; FICi > 0.5 and <4, no interaction; FICi > 4, antagonistic.^44^

### Scanning electron microscopy

Since complex 6 exhibited the highest activity among all tested compounds, its effect on bacterial cells was further investigated using scanning electron microscopy against Gram-positive *Staphylococcus aureus* (MTCC 96) and Gram-negative *Escherichia coli* (MTCC 111).^48,49^ The bacterial cultures were treated with complex 6 at a concentration equal to its MIC at 37 °C for 24 hours. Following incubation, the cells were fixed with 2.5% glutaraldehyde for 2 hours, washed thrice with 0.1 M phosphate buffer saline (PBS) and dehydrated by ethanol washes (30%, 40%, 50%, 60%, 70%, 80%, 90%; for 10 minutes each and 100%; twice for 30 min). The samples were then air dried and coated with a thin layer of gold (5 nm) in a sputter coater. The images were obtained at different magnifications using Sigma 300 VP (Carl Zeiss) operating at an accelerating voltage of 5 kV.

### Inner membrane permeabilization assay

In this assay, the release of cytoplasmic β-galactosidase from live bacteria was quantified based on their reaction to the substrate *o*-nitrophenyl-β-d-galactoside (ONPG).^50^ The bacterial inoculum was prepared by harvesting an overnight culture of bacteria grown in nutrient broth. The collected cells were washed and resuspended in 0.01 mol L^−1^ PBS solution, with the absorbance adjusted to 1.2 at 420 nm (*A*_420_). Solutions of complex 6 (100 µL) at different concentrations were added to 100 µL of bacterial inoculum in PBS. 10 µL of 30 mM ONPG was added to the mixture for both Gram-positive and Gram-negative strains. A control experiment was also conducted without any test compound. An increase in absorbance at 420 nm (*A*_420_) over time indicated the formation of *o*-nitrophenol, which was monitored at one-hour intervals up to 24 hours.

### Assessment of membrane permeabilization by propidium iodide staining

Permeability of the membrane due to membrane-damage induced by the compounds in *S. aureus* (MTCC 96) and *E. coli* (MTCC 111) was evaluated by propidium iodide (PI) staining with the help of fluorescence microscopy.^51^ Compound 6 was used as a representative compound for the study. Bacterial cultures were prepared by harvesting an overnight culture of bacteria grown in nutrient broth. The bacterial suspensions were then incubated overnight (15 hours) with the test compound at its MIC for each strain. After treatment, the cells were collected by centrifugation, washed twice with PBS and resuspended in the same. Propidium iodide was added to each sample to a final concentration of 10 µg mL^−1^ and incubated in the dark at room temperature for 15 minutes. Untreated cells served as the negative controls. Excess dye was removed by gentle washing with PBS. A small aliquot of the stained cell suspension was placed on a glass slide, covered with a coverslip, and examined under a Zeiss Axiovert 5 inverted fluorescence microscope equipped with an A-Plan 100×/1.25 Oil objective, Filter Set 109 HE LED and Axiocam 202 mono camera (ZEISS, Germany). The samples were excited at 565 nm (Green) by a 4-channel solid-state light source (Colibri 3 Type RGB-UV). The images were acquired using ZEISS ZEN 3.12 software.

### Cytotoxicity assay (MTT)

The cytotoxic effects of complexes 5 and 6 were evaluated in human dermal fibroblast (HDF) cells using the MTT assay, a colorimetric method based on the reduction of a tetrazolium salt to insoluble formazan by metabolically active cells.^52^ HDF cells were cultured in α-MEM supplemented with fetal bovine serum and penicillin-streptomycin at 37 °C in a humidified 5% CO_2_ atmosphere (HiMedia, India). For the assay, 200 µL of cell suspension containing 1 × 10^4^ cells per well was seeded into 96-well plates and incubated for 24 hours to allow attachment. The cells were then treated with complexes 5 and 6 at concentrations of 1.45, 2.9, 5.8, 11.6 and 23.2 µg mL^−1^ for 24 hours. Medium without cells served as the blank, untreated cells as the negative control, and doxorubicin (2.5 µM) as the positive control.

After treatment, the spent medium was removed, and MTT was added to each well to obtain a final concentration of 0.5 mg mL^−1^. The plates were incubated in the dark for 3 hours at 37 °C to allow formation of formazan crystals. Thereafter, the MTT-containing solution was aspirated, and the formazan crystals were dissolved in 100 µL DMSO with gentle agitation. Absorbance was measured at 570 nm using a microplate reader, and cell viability was calculated relative to the untreated control after subtraction of blank absorbance.

### Statistical analysis

Statistical analyses were two-tailed with a significance threshold of *α* = 0.05. Comparisons of means were performed using an unpaired two-sample *t*-test (two-tailed) *via* GraphPad QuickCalcs (https://www.graphpad.com/quickcalcs/ttest2/; last assessed 22.05.26). Non-parametric comparisons used the Mann–Whitney *U* test (two-tailed) *via* StatsKingdom (https://www.statskingdom.com/170median_mann_whitney.html; last assessed 22.05.26).

## Result and discussion

### Synthesis and general aspects

**Fig. 1:**
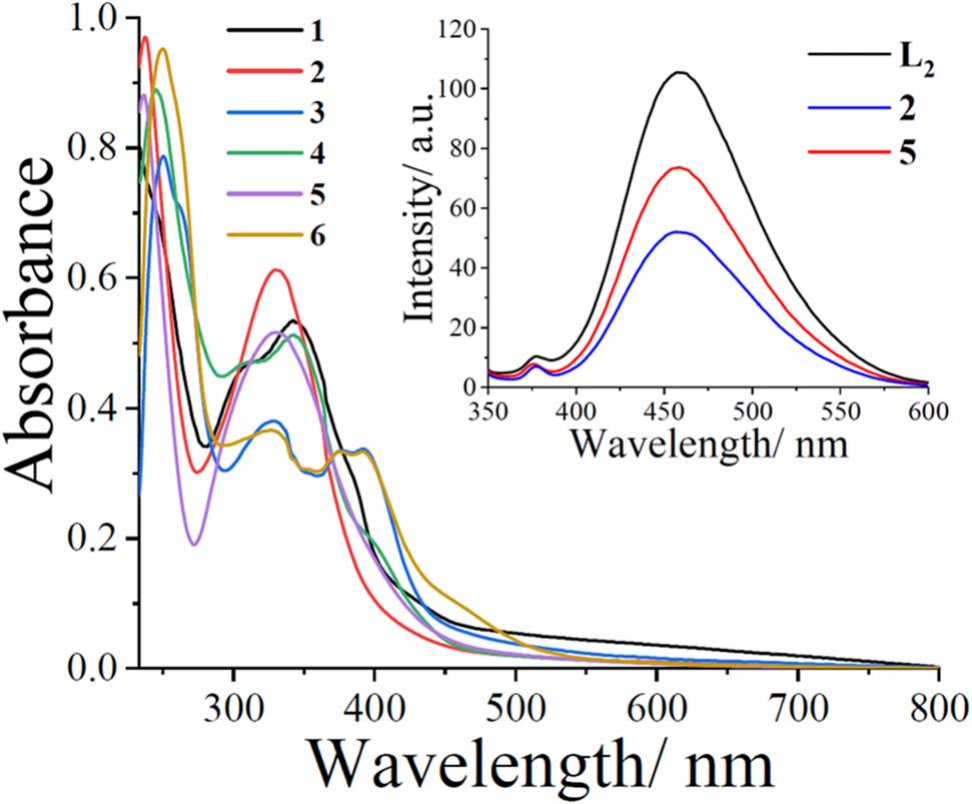
UV-visible spectra of the complexes 1–6 (1 mM) in DMSO–Tris–HCl buffer (pH 7.2) (1 : 4 v/v). The inset shows the emission spectra of naphthyl flavone (L_2_) and its corresponding complexes 2 and 5 (10 µM) in the same solvent.

The 3-hydroxy flavone ligands L_1_–L_3_ were synthesized following a reported procedure and characterized by various analytical methods.^39^ The complexes 1–3 were prepared by reaction of Sn(ii) chloride dihydrate with 3-hydroxy flavone ligands in methanol. Complexes 4–6 were obtained following a similar synthetic procedure using Sb(iii) chloride as the metal precursor. The compounds were thoroughly characterized by various spectroscopic and analytical techniques. The IR spectra of the 3-hydroxyflavone ligands (L_1_–L_3_) demonstrated characteristic peaks at ∼1640 and ∼1560 cm^−1^ corresponding to C=O and C=C groups of the flavones (Fig. S1–S3, SI). However, the complexes (1–6) displayed characteristic peaks at ∼1610 and ∼1505 cm^−1^ corresponding to C=O and C=C groups of the flavone ligand coordinated to the metal centre (Fig. S4–S9, SI). In comparison to the free ligands, the C=O stretching bands shift significantly towards lower frequencies, indicating the participation of oxygen atoms in metal coordination. The UV-visible spectra of the ligands and their respective complexes were obtained in DMSO–Tris–HCl buffer (pH 7.2) (1 : 4 v/v). The three synthesised ligands show characteristic absorption bands in the range of 230–240 corresponding to π → π* transitions of the benzoyl moiety and at around 300–350 due to n → π* transitions within the cinnamoyl segment of the flavones. Upon coordination to the metal ions, the bands undergo a red shift, as evident from the absorption spectra of the complexes (Fig. 1, S10 SI). The ligands showed an emission band with a maximum at ∼470 nm upon excitation at ∼340 nm in DMSO at ambient temperature. Upon complexation, quenching of the fluorescence is observed for both Sn(iv) and Sb(III) complexes (Fig. 1, S11–S13, SI). This result can be attributed to the heavy metal effect of tin and antimony, promoting intersystem crossing and enhancing non-radiative decay pathways.

The solution phase stability of the ligands and corresponding metal complexes 1–6 was evident from their high-resolution mass spectra (HRMS) in acetonitrile. The ligands showed base peak corresponding to [M + H]^+^ whereas complexes 1–3 displayed [M]^+^ ions and complexes 4–6 exhibited [M–Cl]^+^ ions respectively (Fig. S14–S22, SI). The ^1^H NMR spectra of the ligands L_1_–L_3_ exhibit a consistent aromatic proton pattern characteristic of 3-hydroxyflavones (Fig. S23–S25, SI).^53,54^ The variation in chemical shift in ^1^H NMR signals for the complexes 1–6 arises mainly due to redistribution of electron density in the ligand framework as a result of metal coordination. In case of the complex 1 and 4 bearing 3-hydroxyflavone (L_1_) as ligands, deshielding of the chromone protons leads to slight downfield shifts indicating coordination of the ligands to the metal centre. Similarly, in complexes 2 and 5 having naphthyl flavone (L_2_) as ligands, the *ortho*-proton of the flavone ring B exhibits a minor downfield shift suggesting a decrease in electron density around this proton resulting from metal coordination. The complexes 3 and 6, having anthracenyl flavone (L_3_) as ligands show no such changes, however, the absence of the phenolic OH proton signal indicates successful metal coordination through 3-hydroxy-4-keto site of the flavone (Fig. S29–S34, SI). The ^13^C NMR signals of the free ligands were assigned based on available literature.^55,56^ (Fig. S26–S28, SI). For the complexes, the downfield shift of the carbonyl carbon indicates its participation in complex formation. (Fig. S35–S40, SI).

### Solubility

The complexes 1–6 are soluble in acetone, acetonitrile, DMF, DMSO; moderately soluble in methanol, ethanol and insoluble in diethyl ether, hexane, toluene, petroleum ether, and hydrocarbon solvents.

### Antibacterial activity

**Fig. 2:**
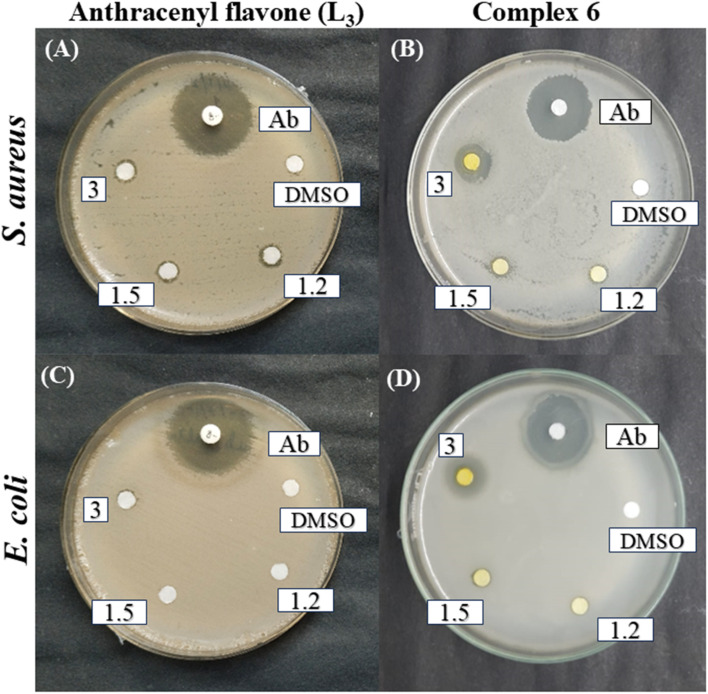
Zone of inhibition of the ligand L_3_ [(A) and (C)] and its corresponding Sb(iii) complex 6 [(B) and (D)] against *S. aureus* and *E. coli*. [Here, Ab indicates the reference antibiotic (positive control), while DMSO serves as the negative (solvent) control. The numerical values (3, 1.5 and 1.2) indicate the concentrations (mg mL^−1^) of the test compounds used for the assay.].

The antibacterial activity of the ligands, metal salts and synthesized complexes was evaluated against *Staphylococcus aureus* (MTCC 96), *Escherichia coli* (MTCC 111), *Pseudomonas aeruginosa* (MTCC 1688) and *Klebsiella pneumoniae* (MTCC 432) by measuring the zones of inhibition (ZOI) using the disc diffusion method (Table 1, Fig. 2, S32–S39, SI).^40^ Zones of inhibition were significantly greater for antimony-based compounds than for tin-based compounds (Mann–Whitney *U* test: *U* = 1694, *Z* = 4.031, *p* < 0.001; mean ranks 59.79 *vs.* 37.21; *n* = 48 per group).

**Table 1:** Zone of inhibition displayed by the complexes 1–6, L_1_–L_3_, metal salts and ciprofloxacin against *S. aureus*, *E. coli*, *P. aeruginosa* and *K. pneumoniae*^a^

Compounds	Concentration (mg mL^−1^)	Zone of inhibition (mm)			
		*S. aureus*	*E. coli*	*P. aeruginosa*	*K. pneumoniae*
L_1_	3	6.10 ± 0.14	6.55 ± 0.64	6.15 ± 1.57	6.25 ± 1.62
	1.5	6.05 ± 0.07	6.20 ± 0.28	0	0
	1.2	0	0	0	0
L_2_	3	6.20 ± 0.14	6.35 ± 0.35	6.30 ± 0.28	6.15 ± 0.21
	1.5	6.35 ± 0.07	6.30 ± 0.15	0	0
	1.2	6.15 ± 0.07	0	0	0
L_3_	3	6.45 ± 0.21	6.60 ± 0.56	6.20 ± 0.14	6.15 ± 0.21
	1.5	6.25 ± 0.21	6.05 ± 0.07	0	0
	1.2	0	0	0	0
SnCl_2_	3	6.30 ± 0.14	0	6.90 ± 0.14	6.05 ± 0.07
	1.5	6.05 ± 0.07	0	6.20 ± 0.14	0
	1.2	0	0	6.05 ± 0.07	0
SbCl_3_	3	6.10 ± 0.14	6.05 ± 0.07	7.00 ± 0.14	6.35 ± 0.21
	1.5	0	0	6.65 ± 0.21	6.15 ± 0.21
	1.2	0	0	6.35 ± 0.07	0
Complex 1	3	6.25 ± 0.07	6.35 ± 0.35	6.10 ± 0.14	6.10 ± 0.14
	1.5	6.05 ± 0.07	6.20 ± 0.28	6.20 ± 0.14	0
	1.2	0	0	6.05 ± 0.07	0
Complex 2	3	6.35 ± 0.07	6.50 ± 0.28	6.20 ± 0.28	6.35 ± 0.21
	1.5	6.05 ± 0.07	6.10 ± 0.14	6.05 ± 0.07	6.30 ± 0.28
	1.2	0	0	0	0
Complex 3	3	6.35 ± 0.35	6.35 ± 0.07	6.10 ± 0.14	6.15 ± 0.21
	1.5	6.10 ± 0.14	6.10 ± 0.14	6.05 ± 0.07	6.35 ± 0.35
	1.2	0	0	0	0
Complex 4	3	6.65 ± 0.21	7.45 ± 1.41	6.75 ± 0.35	6.55 ± 0.35
	1.5	6.25 ± 0.21	7.25 ± 0.07	6.65 ± 0.21	6.40 ± 0.14
	1.2	6.15 ± 0.21	7.15 ± 0.91	6.15 ± 0.21	6.25 ± 0.12
Complex 5	3	6.60 ± 0.14	7.25 ± 2.82	7.20 ± 0.14	7.05 ± 0.21
	1.5	6.20 ± 0.28	7.10 ± 0.14	6.90 ± 0.14	6.80 ± 0.28
	1.2	6.15 ± 0.21	7.05 ± 0.21	6.65 ± 0.21	6.10 ± 0.14
Complex 6	3	9.00 ± 0.84	8.15 ± 0.70	7.35 ± 0.35	7.15 ± 0.21
	1.5	6.20 ± 0.14	7.15 ± 0.07	7.10 ± 0.56	6.80 ± 0.42
	1.2	6.05 ± 0.07	6.20 ± 0.14	6.80 ± 0.42	6.20 ± 0.28
Ciprofloxacin	0.02	23.00 ± 0.70	27.00 ± 1.41	13.50 ± 0.70	23.00 ± 0.41

a Where, L_1_ is 3-hydroxy flavone; L_2_ is naphthyl flavone; L_3_ is anthracenyl flavone and their corresponding Sn(iv) and Sb(iii) complexes having the formulation, [Sn(L)_3_]Cl (1–3) and [Sb(L)_2_Cl] (4–6) where, L = L_1_ (in 1 and 4), L_2_ (in 2 and 5) and L_3_ (in 3 and 6) respectively.

The free ligands (L_1_–L_3_) and metal salts (SnCl_2_ and SbCl_3_) showed limited or no activity, especially at lower concentrations. Complexes 1–3 displayed similar marginal activity with inhibition zones in the range of 6.05 ± 0.07 to 6.50 ± 0.28 mm for all the four strains. In contrast, complexes 4–6 exhibited improved activity, especially against *S. aureus* and *E. coli*. On the other hand, the activity was moderate for *P. aeruginosa* and *K. pneumoniae* with ZOI ranging from 6.15 ± 0.21 to 7.35 ± 0.35 mm. Among the tested compounds, complex 6 showed the most pronounced antibacterial activity, particularly at the highest concentration employed (3 mg mL^−1^). Complex 6 showed a significantly higher ZOI than the ligand L_3_ [(t(6) = 5.24, *p* = 0.0019] (Fig. 2). The complex produced zones of inhibition of 9.00 ± 0.84 mm against *S. aureus* and 8.15 ± 0.70 mm against *E. coli*. It exhibited zones of inhibition against *P. aeruginosa* (7.35 ± 0.35) and *K. pneumoniae* (7.15 ± 0.21) at 3 mg mL^−1^ suggesting superior activity than the ligand. Complexes 4 and 5 also exhibited improved antibacterial activity compared to their corresponding free ligands. These complexes produced zones of inhibition in the range of 6.10 ± 0.14 to 7.45 ± 1.14 mm, respectively, against all the four tested bacteria.

Although ciprofloxacin remained significantly more active, with ZOI in the range of 13.50 ± 0.70 to 27.00 ± 1.41 mm against the tested strains, these recorded activities are significantly higher than those reported in previous reports at similar or higher concentrations.^57–65^ For instance, Sb(v) dicarboxylates bearing cinnamate moieties are reported to show ZOI ranging from 7.00 ± 0.50 to 29.00 ± 1.23 mm against the four strains tested in this study at 10 mg mL^−1^.^57^ Furthermore, certain remarkably potent thiosemicarbazone complexes of Sb(iii) displayed larger ZOI ranging from 33 mm to 36 mm against *S. aureus* and *E. coli* strains, these effects required substantially higher concentration of 20 mg mL^−1^ for disc impregnation.^58^

Similarly, several Sn(iv) Schiff base complexes exhibited significant activity with ZOI ranging from 11 to 26 mm against *S. aureus*, *E. coli* and *P. aeruginosa* at a stock concentration of 20 mg mL^−1^.^59,60^ Therefore, the inhibitory effect observed for our complexes at a substantially lower dose of 3 mg mL^−1^ confirms that these compounds possess sufficient intrinsic potency to target bacterial cells. However, it must be noted that some dithiocarbamate complexes of Sb(iii) are reported to demonstrate superior activity, yielding ZOI of 10.67 ± 1.53, 13.00 ± 1.00 and 9.00 ± 1.00 mm for *S. aureus*, *E. coli* and *K. pneumoniae*, respectively, at a concentration of only 0.05 mg mL^−1^.^61^

This suggests a higher inherent activity of the specific ligand structural class. Despite these comparisons, the antibacterial effect observed for the novel Sn(iv) and Sb(iii) flavonoids presented here is profoundly significant in the current environment dominated by the crisis of antimicrobial resistance.

### Determination of MIC using resazurin-based microdilution assay

The antibacterial activity of the compounds was quantified by determining their MIC values using a resazurin-based microdilution assay against the bacterial strains *S. aureus*, *E. coli*, *P. aeruginosa* and *K. pneumoniae*.^41,42^ The MIC values of the Sb(iii) complexes *i.e.*4–6 displayed markedly enhanced antibacterial activity compared to their ligands and metal salt precursors. Complex 6 showed the lowest MIC of 2.9 µg mL^−1^ (3.48 µM) against *S. aureus*, equal to that of the standard antibiotic ciprofloxacin. Among the Gram-negative strains, *E. coli* showed the highest susceptibility, whereas *P. aeruginosa* was the least susceptible overall, with MICs ranging from 93.7 to 187.5 µg mL^−1^. The complexes 4 and 5 showed relatively high MIC values of 93.7 µg mL^−1^ (148.33 µM) and 5.8 µg mL^−1^ (7.92 µM), respectively, against the *S. aureus* strain. The corresponding values for the other three strains ranged from 23.4 to 375 µg mL^−1^ for complexes 4 and 5, which were higher than those for complex 6. Complexes 5 and 6 remained the most active compounds in the series, with particularly low MIC values against *S. aureus*.

The Sn(iv) complexes (1–3) exhibited no significant antibacterial activity, with high MIC values across all four strains. The free flavonoid ligands (L_1_–L_3_) exhibited significantly higher MIC values (≥187.5 µg mL^−1^) in all four strains, indicating weaker antibacterial properties. Metal salts, *i.e.* SnCl_2_ and SbCl_3_, exhibited moderate activity, but were less effective than their flavonoid complexes (Table 2). Overall, the data suggest that metal complexation significantly improves the antibacterial properties of the ligands, with Sb(iii) complexes showing significantly lower MICs than Sn(iv) complexes (Mann–Whitney U, *Z* = 2.88, *p* = 0.003). The standardised effect size was large (*r* ≈ 0.83), indicating a strong difference in potency between the groups.

**Table 2:** Minimum Inhibitory Concentration (MIC) of the complexes 1–6, L_1_–L_3_, metal salts and ciprofloxacin against growth of *S. aureus*, *E. coli*, *P. aeruginosa* and *K. pneumoniae*^a^

Compounds	MIC (µg mL^−1^)			
	*S. aureus*	*E. coli*	*P. aeruginosa*	*K. pneumoniae*
L_1_	750	1500	375	375
L_2_	187.5	375	375	375
L_3_	750	750	750	375
SnCl_2_	375	375	375	187.5
SbCl_3_	187.5	750	375	93.75
Complex 1	187.5	375	187.5	375
Complex 2	375	187.5	375	375
Complex 3	375	375	187.5	375
Complex 4	93.7	23.4	187.5	187.5
Complex 5	5.8	93.7	375	187.5
Complex 6	2.9	93.7	187.5	93.7
Ciprofloxacin	2.9	0.18	23.4	11.7
DMSO	375	375	375	375

a Where, L_1_ is 3-hydroxy flavone; L_2_ is naphthyl flavone; L_3_ is anthracenyl flavone and their corresponding Sn(iv) and Sb(iii) complexes having the formulation, [Sn(L)_3_]Cl (1–3) and [Sb(L)_2_Cl] (4–6) where, L = L_1_ (in 1 and 4), L_2_ (in 2 and 5) and L_3_ (in 3 and 6) respectively.

This enhanced antibacterial efficacy of metal complexes has been explained in the literature with the help of Tweedy's chelation theory and Overtone's concept.^62,66^ According to the chelation theory, the overlap between ligand orbitals and metal ion enables partial delocalisation of the metal's positive charge onto the coordinating groups. This redistribution of charge across the chelated complex markedly reduces the polarity of the metal centre, thereby improving lipophilicity. In line with Overtone's concept, a more lipophilic compound exhibits greater affinity for lipid-rich membranes. This enhanced lipid solubility allows more efficient penetration through the microbial membrane, contributing to the observed antibacterial effect.^46,59,67–69^ Furthermore, the MIC values observed for the Sb(iii)-flavonoids are notably lower than many antimony-based complexes reported earlier.^55,63,70,71^ Most of these Sb(iii) complexes exhibit MIC values in the range of 5.5 to 145 µg mL^−1^ against *S. aureus*, *E. coli*, *P. aeruginosa* and *K. pneumoniae*, whereas the Sb(iii) complexes in the present study displayed substantially improved potency with MIC values as low as 2.9 µg mL^−1^ for *S. aureus*. This increase in activity probably arises from the synergistic contribution from the intrinsic antibacterial properties of flavonoids and the antimony centre.^37,72^

### Synergy assay

The combination of the complexes 4–6 with ciprofloxacin has been screened for growth inhibitory effect against *S. aureus* and *E. coli* using a synergy assay.^44–47^ The assay revealed a considerable decrease in the MICs of the compounds when combined with ciprofloxacin (Table 3). In case of *S. aureus*, complex 4 did not show a significant decrease in MIC in the synergy assay; however, complexes 5 and 6 showed a 4-fold decrease in MIC and inhibited growth at concentrations 1.4 and 0.7 µg mL^−1^, respectively. A synergistic effect in lowering of MICs was also observed against *E. coli* with ∼2-fold decrease for complex 4 and 8-fold decrease for complex 5. Under similar conditions, complex 6 displayed a 4-fold increase in activity in *E. coli* with a MIC value of 23.4 µg mL^−1^.

**Table 3:** Minimum Inhibitory Concentration (MIC) of the complexes 4–6 in combination with ciprofloxacin^a^

Compounds	MIC (µg mL^−1^)		FICi	
	*S. aureus*	*E. coli*	*S. aureus*	*E. coli*
Complex 4 + ciprofloxacin	46.8	11.7	0.21	1.36
Complex 5 + ciprofloxacin	1.4	11.7	0.06	0.087
Complex 6 + ciprofloxacin	0.7	23.4	0.24	1.73
Ciprofloxacin	2.9	0.18	—	—
DMSO	375	375	—	—

a MIC and FICi values for the combinations were determined using a fixed 1 : 1 complex: ciprofloxacin mixture.

Fractional inhibitory concentration index (FICi) values indicate variable efficacy of drugs when applied in combination and classify the interaction as synergistic (<0.5), indifferent (0.5–4) or antagonistic (>4). The FICi values of the complexes 4–6 in combination with ciprofloxacin were evaluated against *S. aureus* and *E. coli*. All these three complexes show synergistic interactions with ciprofloxacin against *S. aureus* with FICi values of 0.21, 0.06 and 0.24, respectively, indicating enhanced antibacterial activity. For *E. coli*, complexes 4 and 6 fall within the indifferent range, indicating little to no synergistic benefit. In contrast, complex 5 shows synergistic effect (FICi = 0.087), suggesting significant improvement over ciprofloxacin alone. Overall, the data suggest that the Sb(iii) complexes are more effective in augmenting ciprofloxacin activity against *S. aureus* than *E. coli*. These observations can be compared with the synergistic effect observed in a previously reported Cr(iii) complex in combination with ciprofloxacin.^46^ The complex [Cr(phen)]^3+^ exhibited FIC values ranging from 0.096 to 0.191, showing strong synergistic benefits against both *S. aureus* and *E. coli*. Complexes 4–6 potentiate ciprofloxacin against *S. aureus*. However, only complex 5 shows synergy with ciprofloxacin against *E. coli* under these conditions. This indicates that while Sb(iii)-flavonoids can enhance the activity of ciprofloxacin towards Gram-positive bacteria, their synergistic effects are not as broad as those reported for certain transition-metal compounds. Nonetheless, the observed synergy against *S. aureus* highlights the potential of these complexes in synergistic combination therapy.

### Scanning electron microscopy

**Fig. 3:**
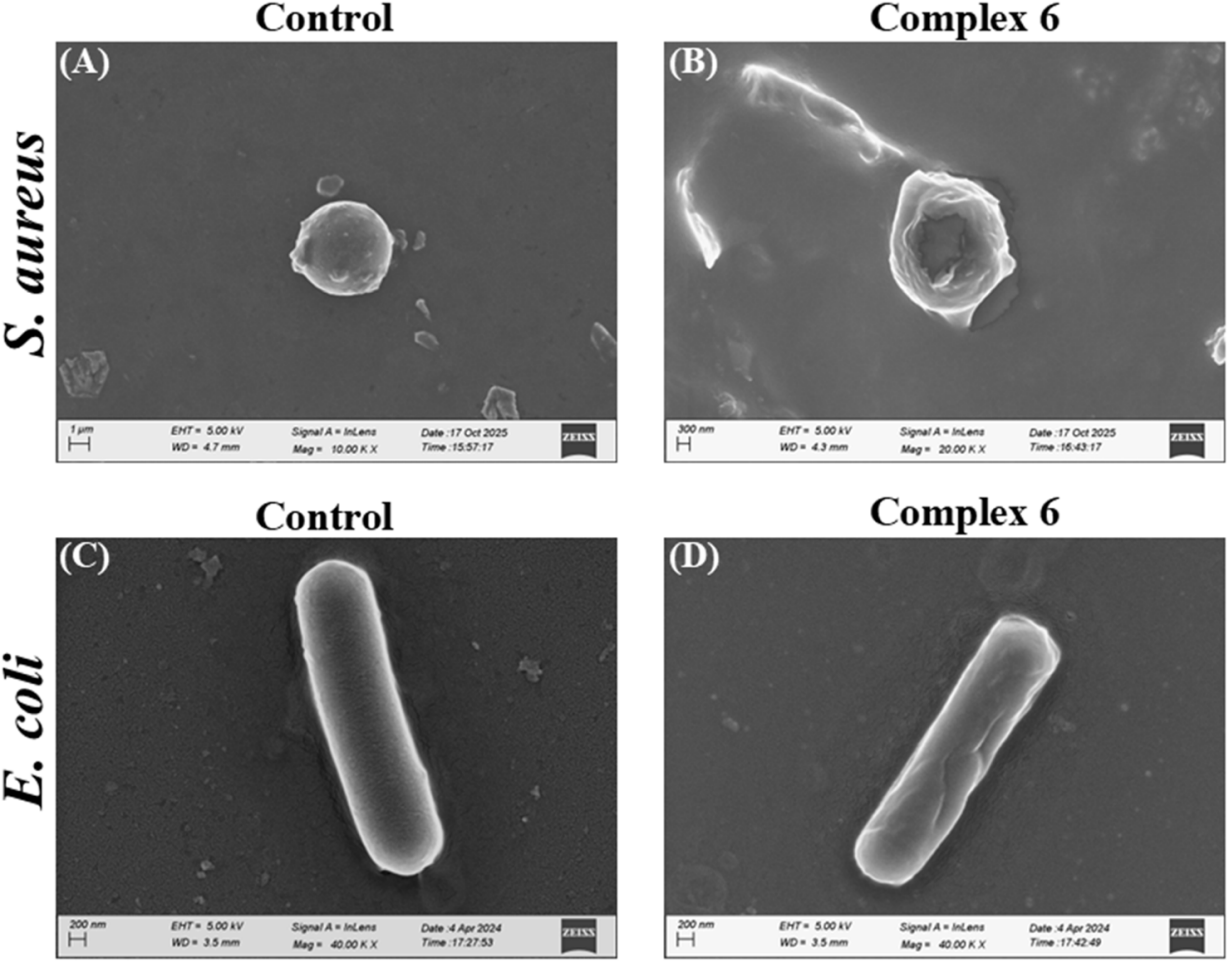
SEM images of *S. aureus* [(A) control cells, (B) cells treated with complex 6] and *E. coli* [(C) control cells, (D) cells treated with complex 6].

The morphological changes in bacterial cells induced by complex 6 were examined in *S. aureus* and *E. coli* using scanning electron microscopy.^48,49^ In case of *S. aureus*, the control cells appear to be spherical with smooth surface and intact morphology (Fig. 3, panel A). However, the cells after treatment revealed significant morphological deformities. The treated cell exhibited a ruptured cell membrane with noticeable pore formation and leakage of intracellular contents (Fig. 3, panel B). These distortions indicate compromised cell wall integrity, suggesting that the treatment disrupted the bacterial membrane, ultimately leading to cell death. For *E. coli*, control cells appeared intact with a smooth surface (Fig. 3, panel C). In contrast, the treated cells displayed evident shrinkage with an irregular, corrugated surface, which is indicative of partial damage to the cell wall (Fig. 3, panel D).

### Inner membrane permeabilization assay

The effect of complex 6 on bacterial cell membrane permeability was evaluated by monitoring the release of cytoplasmic β-galactosidase.^50^ When the bacterial membrane becomes permeable upon treatment with a drug, the chromogenic substrate *o*-nitrophenyl-β-d-galactoside (ONPG) can enter the cells. After entering the cell, it is hydrolysed by β-galactosidase to produce *o*-nitrophenol, which exhibits a yellow colour with a wavelength of maximum absorption at ∼420 nm. Therefore, an increase in absorbance at 420 nm serves as an indirect indicator of enzymatic hydrolysis of ONPG and membrane permeabilization.

**Fig. 4:**
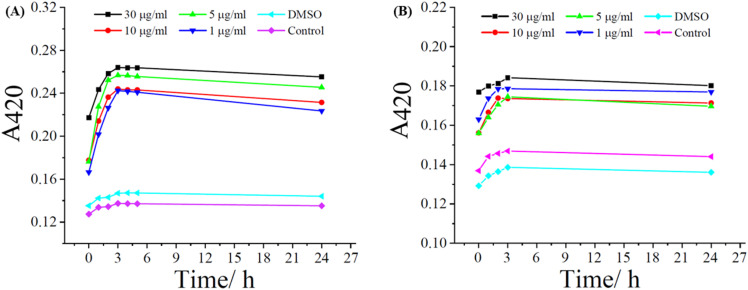
Release of cytoplasmic β-galactosidase by (A) *S. aureus* and (B) *E. coli* treated with complex 6 at various concentrations.

In the case of the Gram-positive *S. aureus* strain, complex 6 induced a concentration-dependent increase in *o*-nitrophenol formation as indicated by the rise of absorbance at 420 nm. All tested concentrations of complex 6 caused a rapid increase in absorbance within the first 3 hours, which suggested disruption of the bacterial cell membrane leading to release of cytoplasmic β-galactosidase (Fig. 4, panel A). A similar trend was observed for *E. coli* treated with complex 6, although the extent of enzyme release was substantially lower than *S. aureus* (Fig. 4, panel B). Overall, it can be concluded that treatment with complex 6 leads to significant damage of cell membrane, although to different extents, in the bacterial strains *S. aureus* and *E. coli*.

### Assessment of membrane permeabilization by propidium iodide staining

**Fig. 5:**
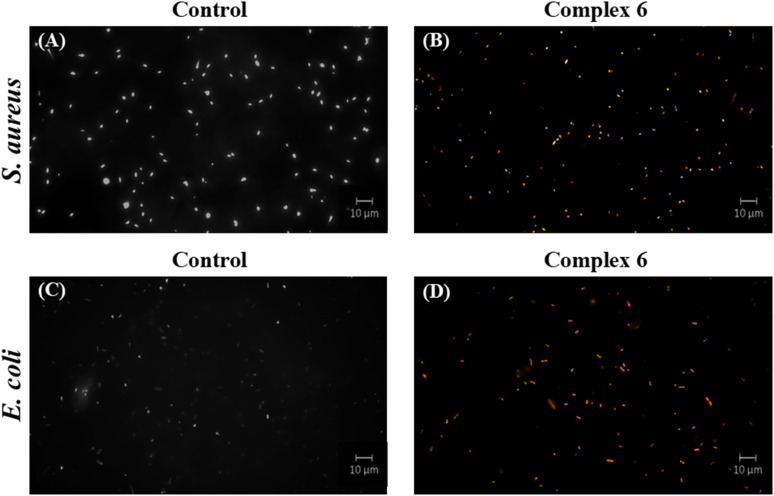
Fluorescence microscope images of *S. aureus* [(A) control cells, (B) cells treated with complex 6] and *E. coli* [(C) control cells, (D) cells treated with complex 6]. Scale bar: 10 µm.

Membrane permeabilization induced by complex 6 in the strains *Staphylococcus aureus* (MTCC 96) and *Escherichia coli* (MTCC 111) was investigated by propidium iodide staining assay using fluorescence microscopy.^51^ Untreated bacterial cells showed negligible red fluorescence indicating intact membranes that effectively excluded PI (Fig. 5, panels A and C). In contrast, cells treated with complex 6 exhibited a marked increase in red fluorescence for both bacterial strains (Fig. 5, panels B and D). This pronounced increase in fluorescence suggests severe disruption of bacterial cell membranes of both *S. aureus* and *E. coli*. The loss of membrane integrity facilitates PI penetration into the cytoplasm, where it binds to nucleic acids and emits strong red fluorescence. Furthermore, this observation of membrane damage correlates with the results of SEM and inner membrane permeability assays, confirming it to be the key mechanism underlying the antibacterial activity of complex 6.

### Cytotoxicity assay (MTT)

The cytotoxic effects of the two most active antibacterial candidates, complexes 5 and 6, were assessed in human dermal fibroblast (HDF) cells using an MTT assay after 24 hours of exposure.^52^ Complex 6 showed HDF viabilities of 99.56%, 97.26%, 93.82%, 48.28% and 41.31% at 1.45, 2.9, 5.8, 11.6 and 23.2 µg mL^−1^, respectively (Fig. S50, SI). Importantly, at its MIC against *S. aureus* (2.9 µg mL^−1^), complex 6 retained 97.26% HDF viability. Complex 5 exhibited HDF viabilities of 99.91%, 97.00%, 90.29%, 82.61% and 76.43% at 1.45, 2.9, 5.8, 11.6 and 23.2 µg mL^−1^, respectively (Fig. S49, SI). At its MIC against *S. aureus* (5.8 µg mL^−1^), complex 5 maintained 90.29% HDF viability. These findings indicate that both complexes display anti-staphylococcal activity at concentrations that did not cause marked toxicity to HDF cells under the tested conditions.

## Conclusion

In this study, 3-hydroxy flavones (L_1_–L_3_) and their corresponding Sn(iv) and Sb(iii) complexes (1–6) were synthesized and evaluated for their antibacterial activity against *Staphylococcus aureus* (MTCC 96), *Escherichia coli* (MTCC 111), *Pseudomonas aeruginosa* (MTCC 1688) and *Klebsiella pneumoniae* (MTCC 432). Complexation of the flavone-based ligands with Sn(iv) and Sb(iii) enhanced antibacterial activity relative to the free ligands and the corresponding metal salt precursors. Among the tested compounds, the Sb(iii) complexes showed the most promising activity in the bacterial panel, with complex 6 emerging as the most active derivative, particularly against *S. aureus*, where its MIC was comparable to that of ciprofloxacin under the tested conditions. In addition, checkerboard analysis demonstrated synergistic interactions between the Sb(iii) complexes and ciprofloxacin against *S. aureus*, as reflected by low FIC index values. Mechanistic studies further supported the antibacterial action of complex 6. Scanning electron microscopy revealed pronounced surface damage in treated bacterial cells, while increased membrane permeability and strong propidium iodide uptake were consistent with membrane permeabilization as a major component of its antibacterial effect. The cytotoxic effects of the two most active antibacterial complexes, 5 and 6, were also assessed in human dermal fibroblast (HDF) cells by MTT assay. Both compounds maintained high HDF viability at their respective MICs against *S. aureus*, indicating that their anti-staphylococcal activity was observed at concentrations that did not cause marked cytotoxicity under the tested conditions. The expanded MIC dataset also revealed a clear organism-dependent susceptibility pattern, with *S. aureus* being more susceptible than the tested Gram-negative bacteria. Overall, these findings identify Sb(iii)-bound flavonoid complexes as promising scaffolds for further antibacterial investigation. However, because the present study was limited to a small panel of reference strains and the tested bacteria remained susceptible to ciprofloxacin, the results should be interpreted as a preliminary assessment of antibacterial potential rather than as evidence of efficacy against resistant clinical isolates. Broader evaluation against clinically relevant resistant bacteria, together with expanded mammalian-cell toxicity profiling, will be necessary to better define the translational potential of these Sn(iv)- and Sb(iii)-containing complexes.

## Conflicts of interest

There are no conflicts to declare.

## Supplementary Material

RA-016-D6RA04588J-s001

## Data Availability

The data supporting this article have been included as part of the supplementary information (SI). Supplementary information: characterization, antibacterial and cytotoxicity studies data (Fig. S1–S50). See DOI: https://doi.org/10.1039/d6ra04588j.
