# Supplementary material for: Synthesis, characterisation and antibacterial activity of flavone-based Sn(iv) and Sb(iii) complexes
Source: RSC Adv. 2026 Jun 5;16(33):30649–61. doi: 10.1039/d6ra04588j (PMC13245310; doi:10.1039/d6ra04588j)
Supplement: RA-016-D6RA04588J-s001 [file RA-016-D6RA04588J-s001.pdf]

Supplementary Information (SI) for RSC Advances.
This journal is © The Royal Society of Chemistry 2026

Electronic Supplementary Information (ESI†)

for

Synthesis, Characterisation and Antibacterial Activity of Flavone Based Sn(IV) And Sb(III) Complexes

Jakia Parbin Sultana,[a] Sunali Saikia,[b] Partha Pratim Saharia,[a] Namisha Das,[a] Debasish Borbora,[*,b] and Tridib K. Goswami [*,a]

[a] *Department of Chemistry, Gauhati University, Guwahati 781014, Assam, India*

[b] *Department of Biotechnology, Gauhati University, Guwahati 781014, Assam, India*

Table of Contents

Fig. S1-S3 IR spectra of the ligands **L_1-L_3** in solid KBr matrix.

Fig. S4-S9 IR spectra of the complexes **1-6** in solid KBr matrix.

Fig. S10 UV-visible spectra of complexes **1-6** (25 μM) in DMSO-Tris-HCl buffer (pH 7.2) (1:4 v/v) showing the ligand centred bands.

Fig. S11-S13 Comparative emission spectra of the ligands **L_1-L_3** with their corresponding Sn(IV) and Sb(III) complexes in DMSO-Tris-HCl buffer (pH 7.2) (1:4 v/v).

Fig. S14-S16 ESI Mass spectra of the ligands **L_1-L_3** in methanol showing the $[M+H]^+$ peaks.

Fig. S17-S22 ESI Mass spectra of the complexes **1-6** in methanol showing $[M]^+$ and $[M-Cl]^+$ peaks.

Fig. S23-S25 ^1H NMR spectra of the ligands **L_1-L_3** in $CDCl_3$.

Fig. S26-S28 ^{13}C NMR spectra of the ligands **L_1-L_3** in $CDCl_3$.

Fig. S29-S34 ^1H NMR spectra of the complexes **1-6** in $CDCl_3$.

Fig. S35-S40 13C NMR spectra of the complexes **1-6** in $CDCl_3$.

Fig. S41 Zone of inhibition (mm) of ligands (**L_1-L_3**) and metal salts against *S. aureus*.

Fig. S42 Zone of inhibition (mm) of the metal complexes **1-6** against *S. aureus*.

Fig. S43 Zone of inhibition (mm) of ligands (**L_1-L_3**) and metal salts against *E. coli*.

Fig. S44 Zone of inhibition (mm) of the metal complexes **1-6** against *E. coli*.

Fig. S45 Zone of inhibition (mm) of ligands (**L_1-L_3**) and metal salts against *P. aeruginosa*.

Fig. S46 Zone of inhibition (mm) of the metal complexes **1-6** against *P. aeruginosa*.

Fig. S47 Zone of inhibition (mm) of ligands (**L_1-L_3**) and metal salts against *K. pneumoniae*.

Fig. S48 Zone of inhibition (mm) of the metal complexes **1-6** against *K. pneumoniae*.

Fig. S49 Mean % cell viability of HDF cells after exposing to complex **5** for 24 hours.

Fig. S50 Mean % cell viability of HDF cells after exposing to complex **6** for 24 hours.

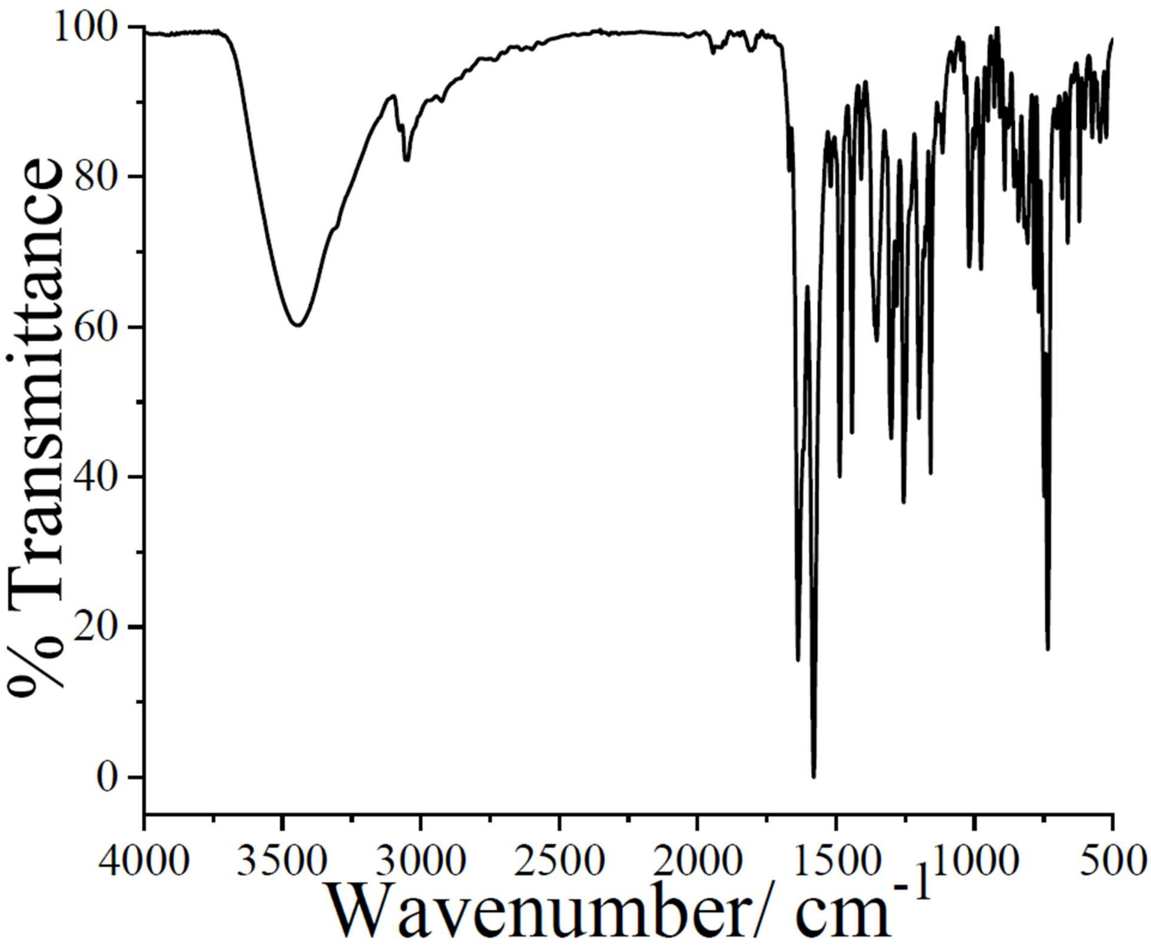

Fig. S1 IR spectra of the ligand $\mathbf{L_1}$ in solid KBr matrix.

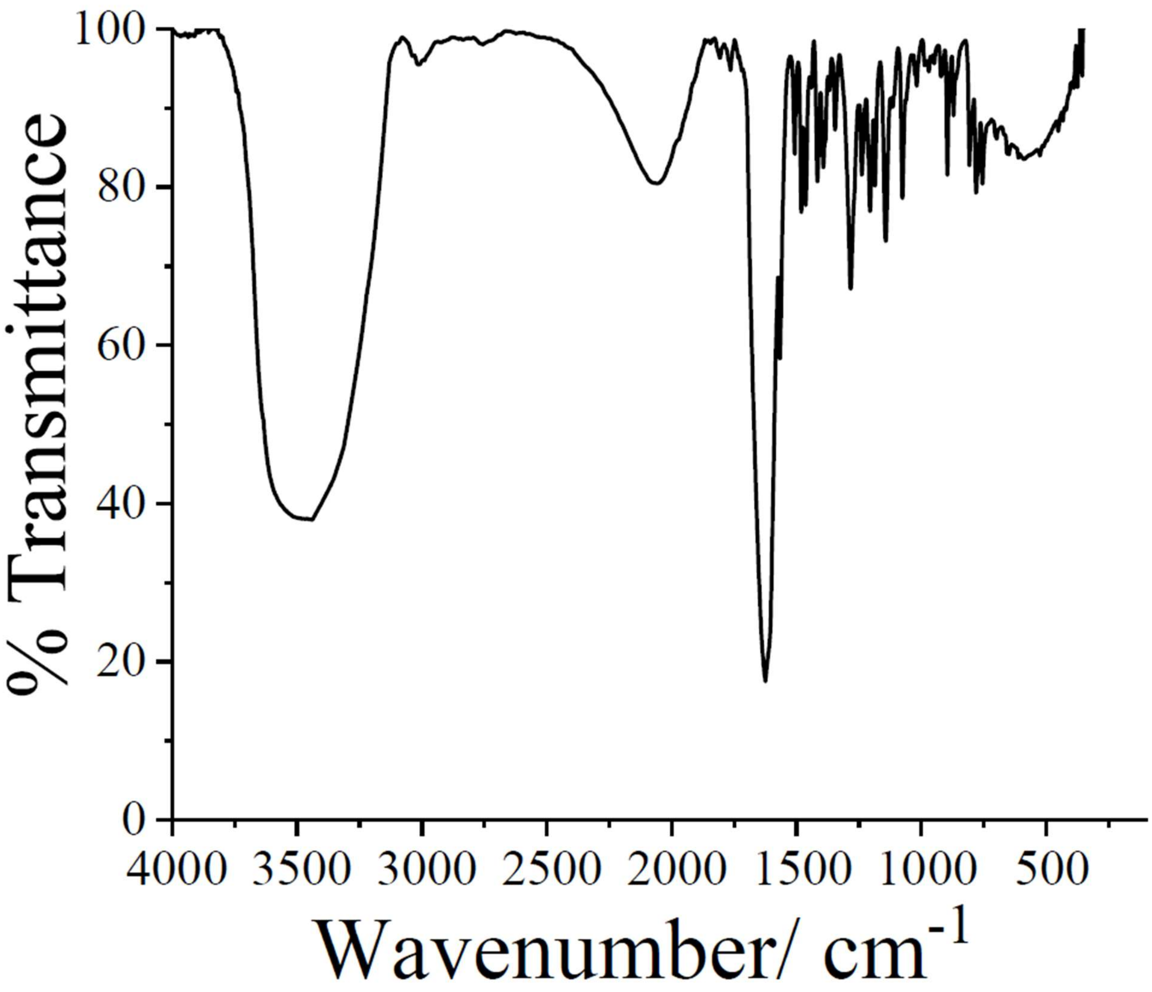

Fig. S2 IR spectra of the ligand **L₂** in solid KBr matrix.

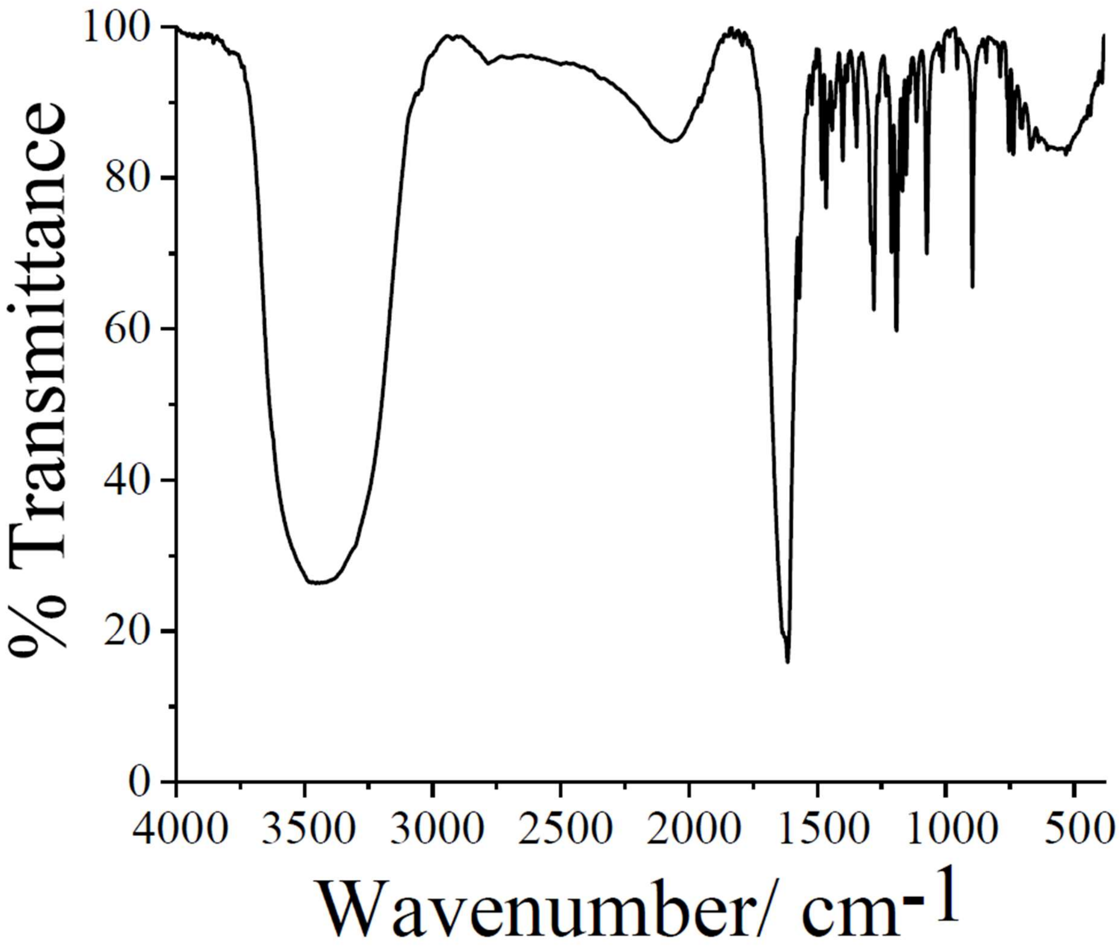

Fig. S3 IR spectra of the ligand $\mathbf{L_3}$ in solid KBr matrix.

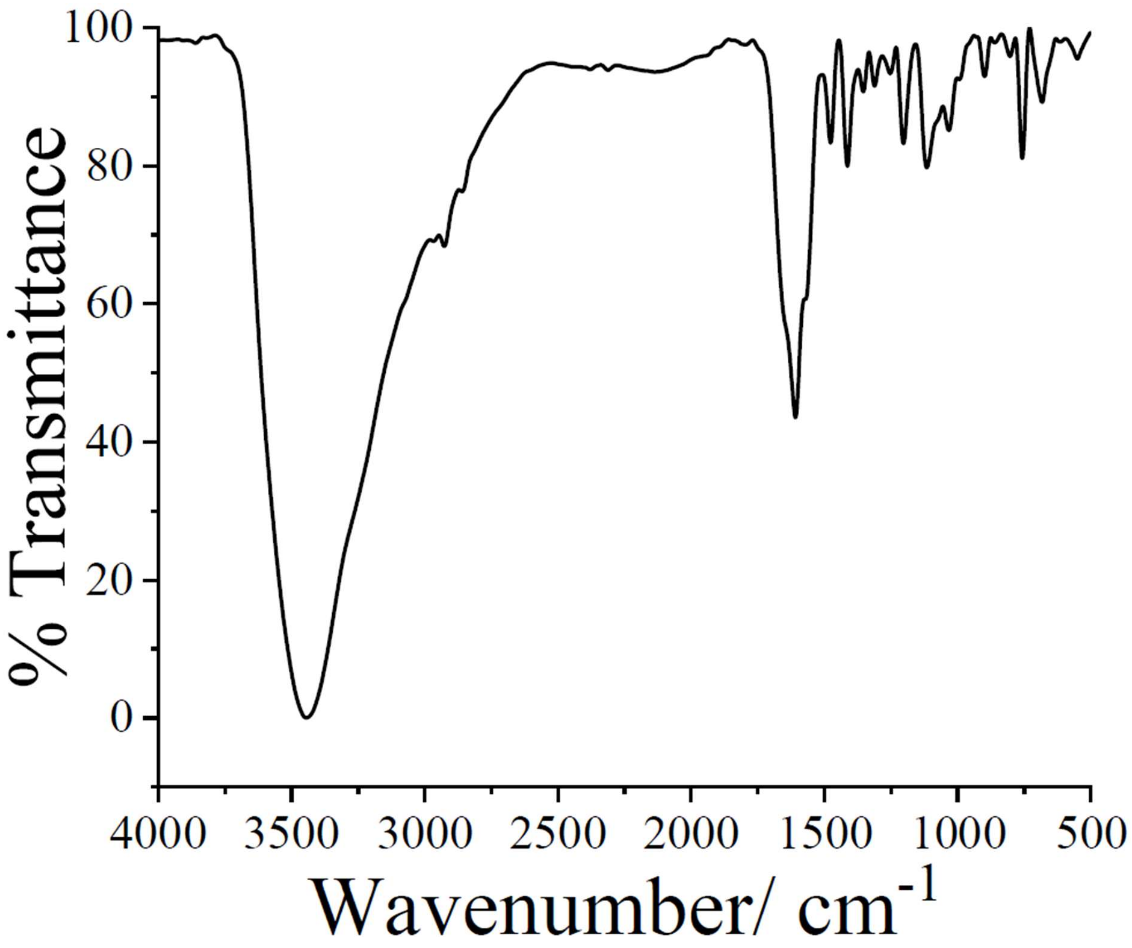

Fig. S4 IR spectra of the complex **1** in solid KBr matrix.

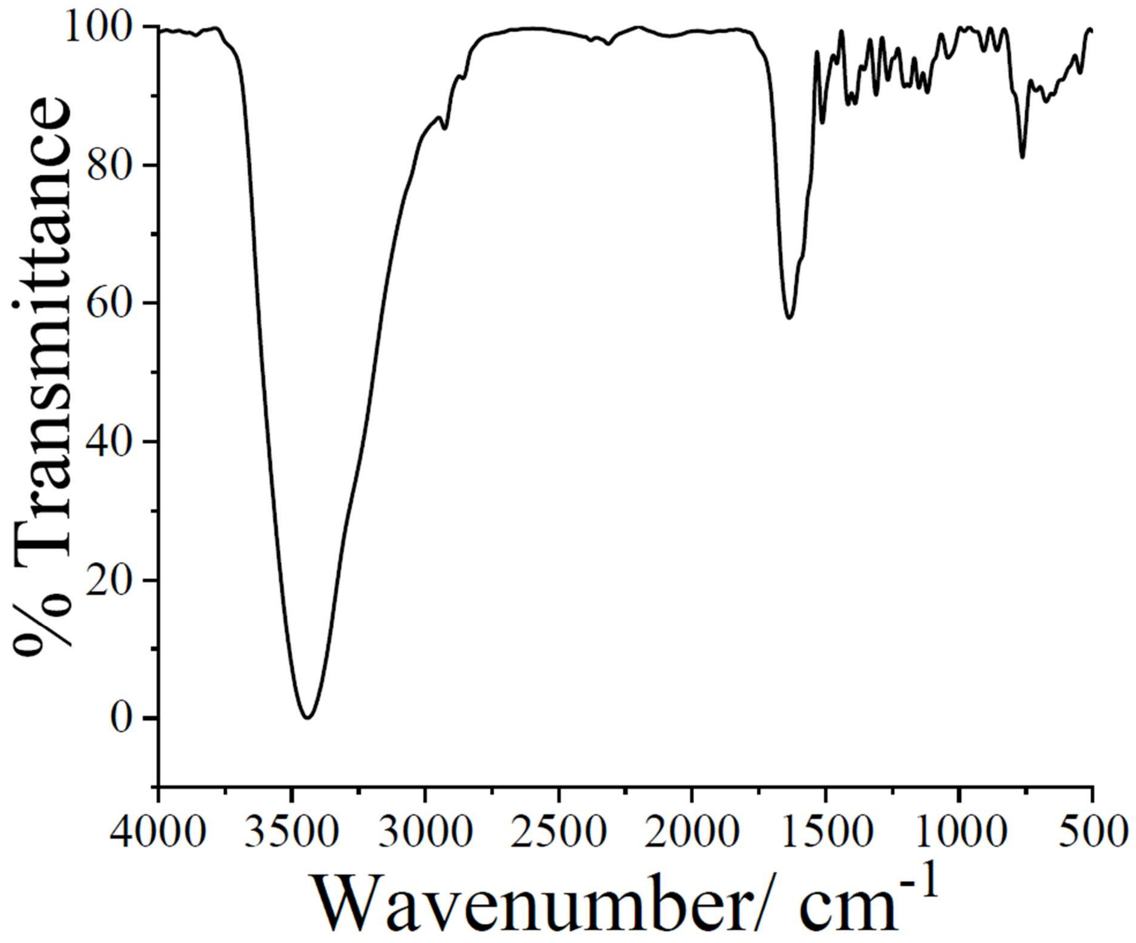

Fig. S5 IR spectra of the complex **2** in solid KBr matrix.

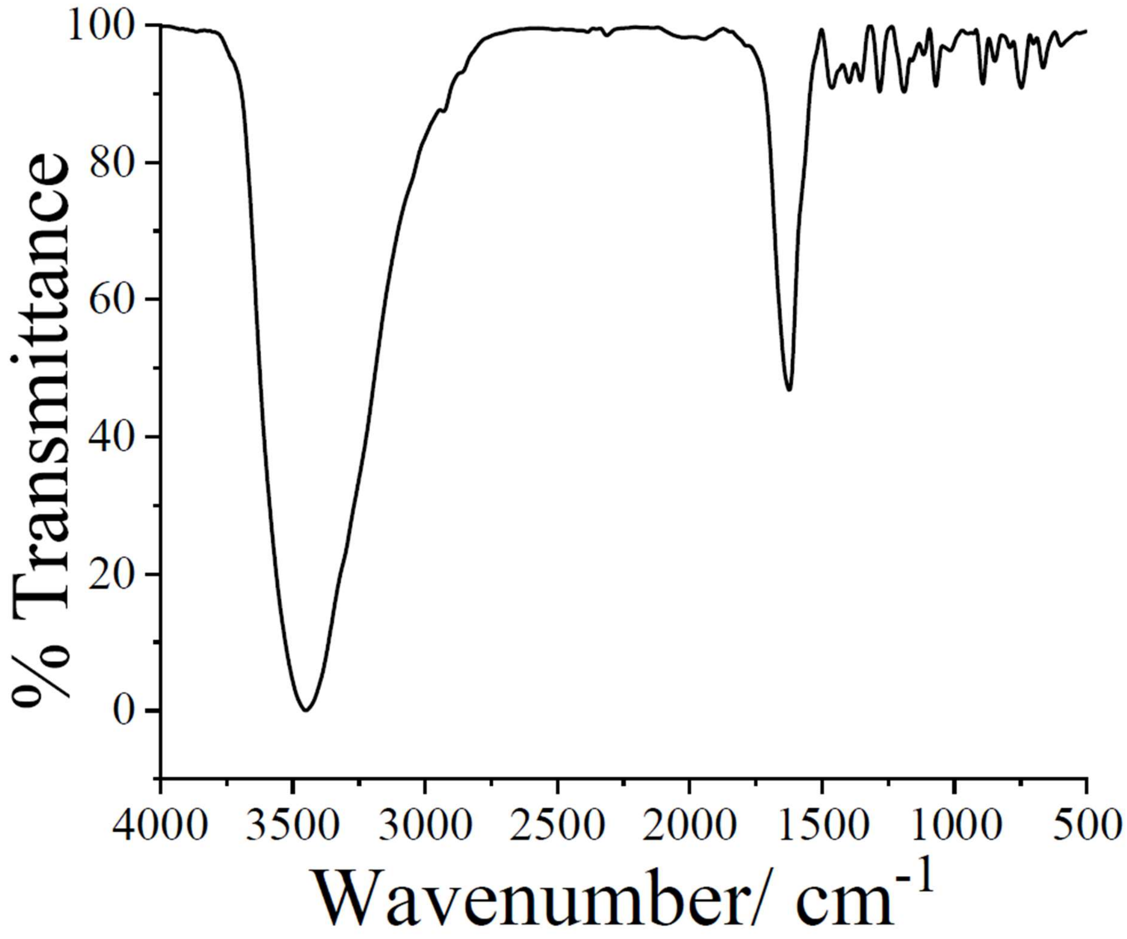

Fig. S6 IR spectra of the complex **3** in solid KBr matrix.

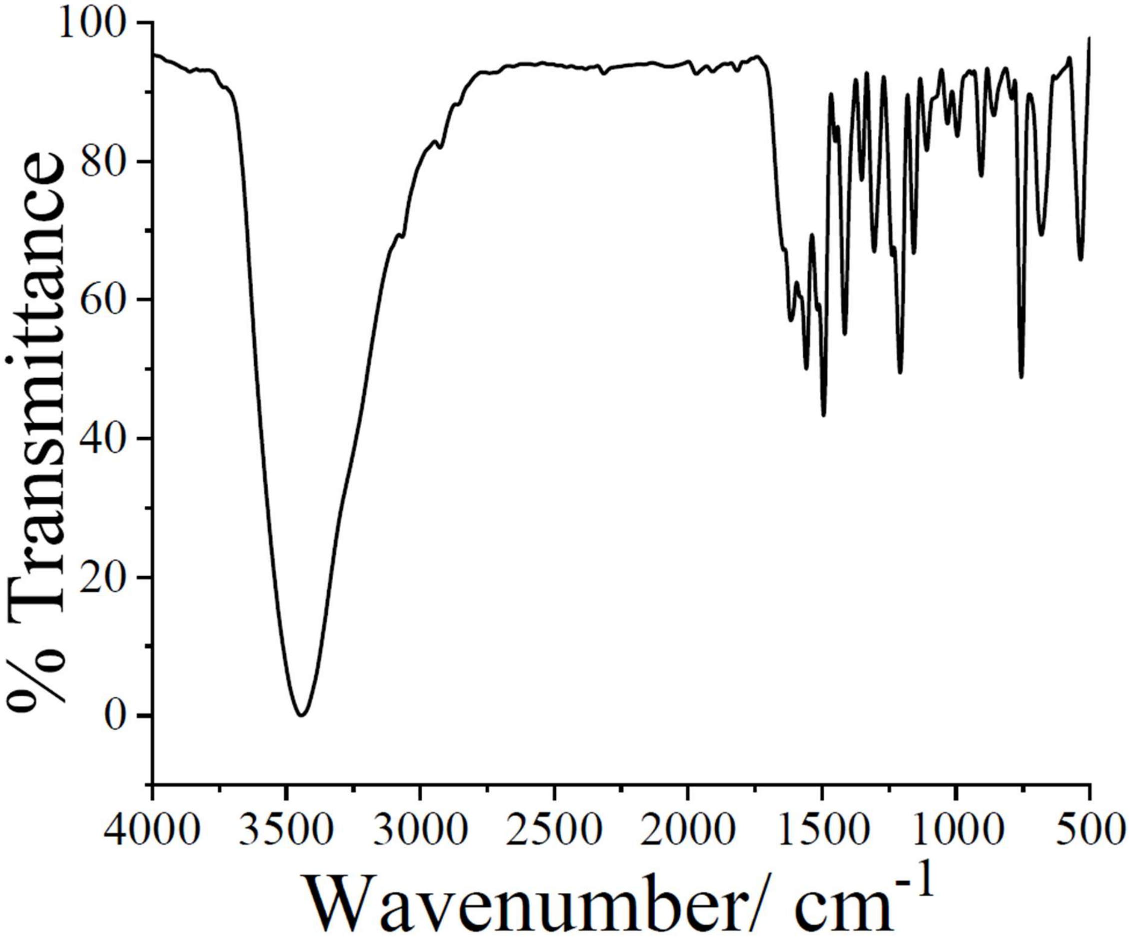

Fig. S7 IR spectra of the complex **4** in solid KBr matrix.

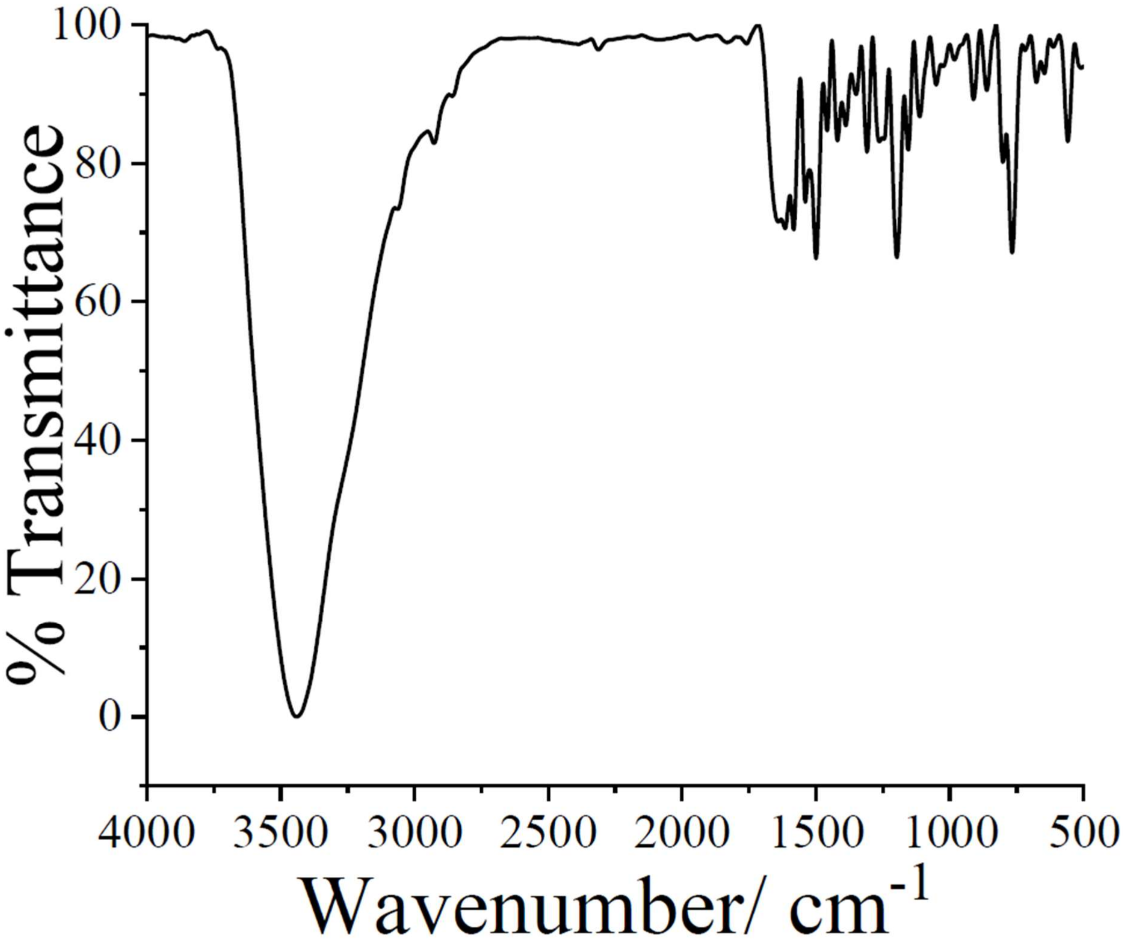

Fig. S8 IR spectra of the complex **5** in solid KBr matrix.

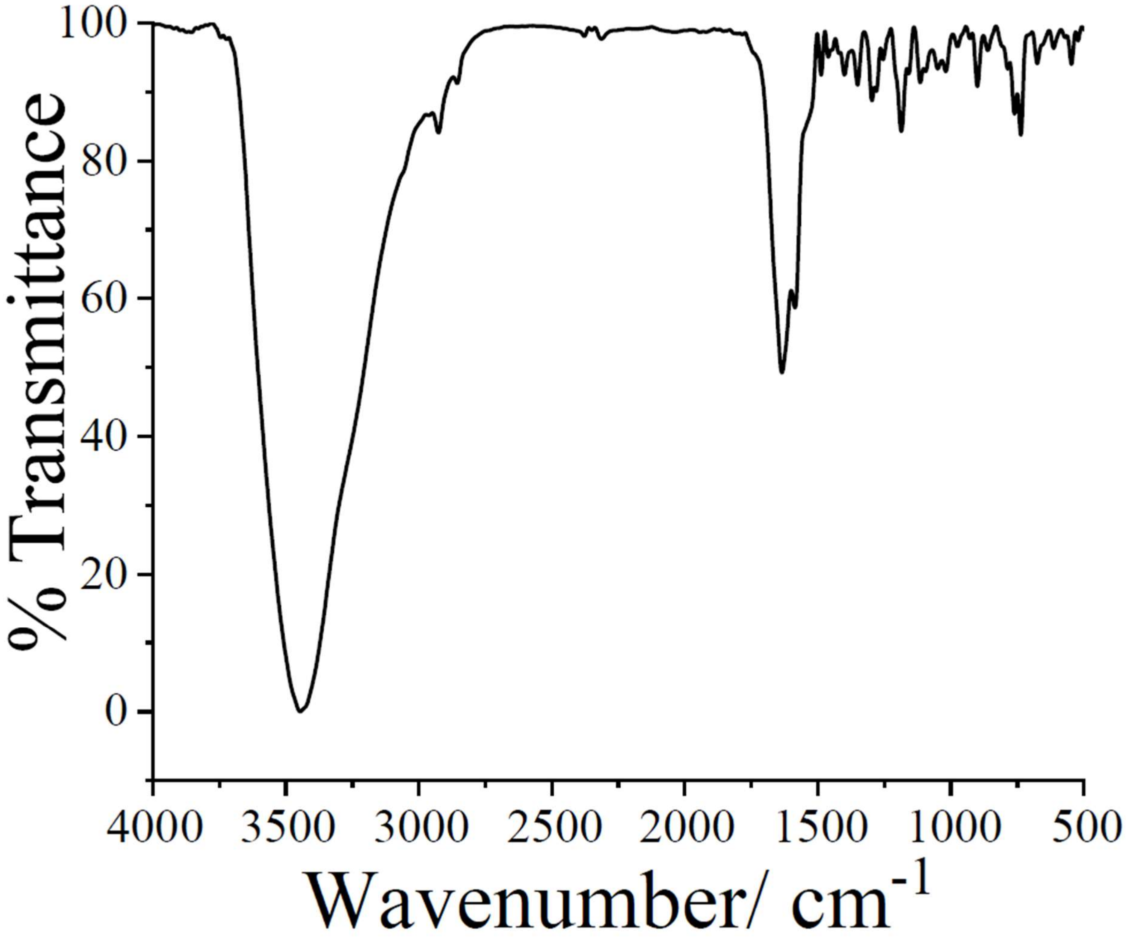

Fig. S9 IR spectra of the complex **6** in solid KBr matrix.

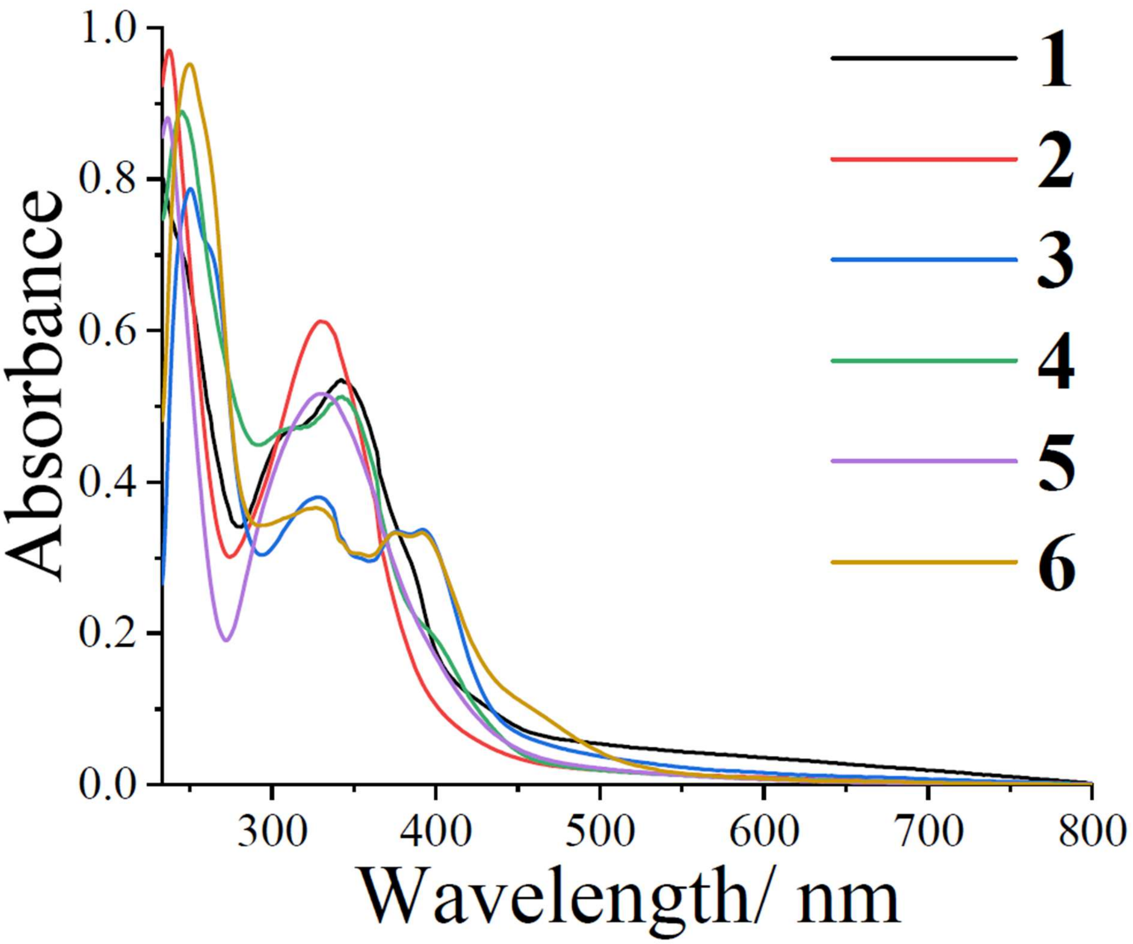

Fig. S10 UV-visible spectra of complexes **1-6** (25 μM) in DMSO-Tris-HCl buffer (pH 7.2) (1:4 v/v) showing the ligand centred bands.

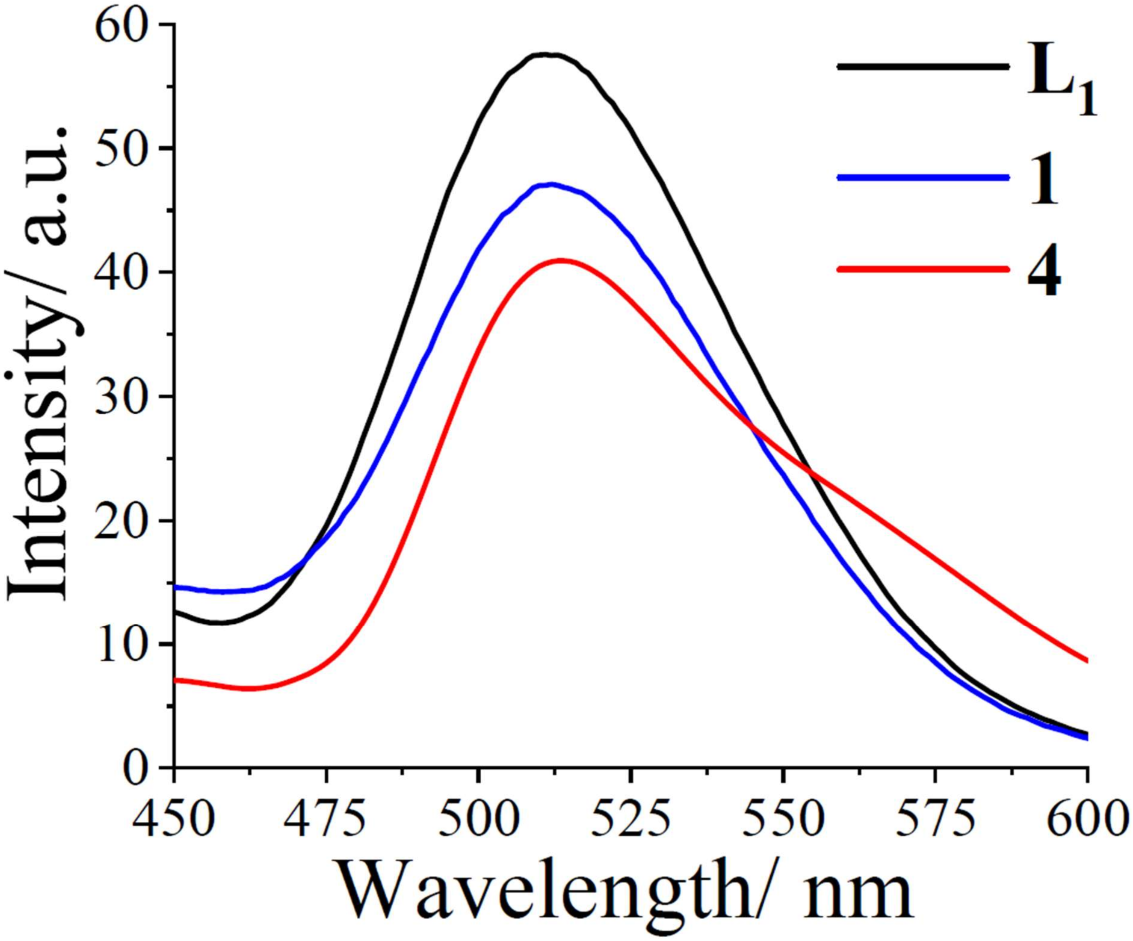

Fig. S11 Emission spectra of ligand **L_1** and the complexes **1** and **4** (10 μM) in DMSO-Tris-HCl buffer (pH 7.2) (1:4 v/v).

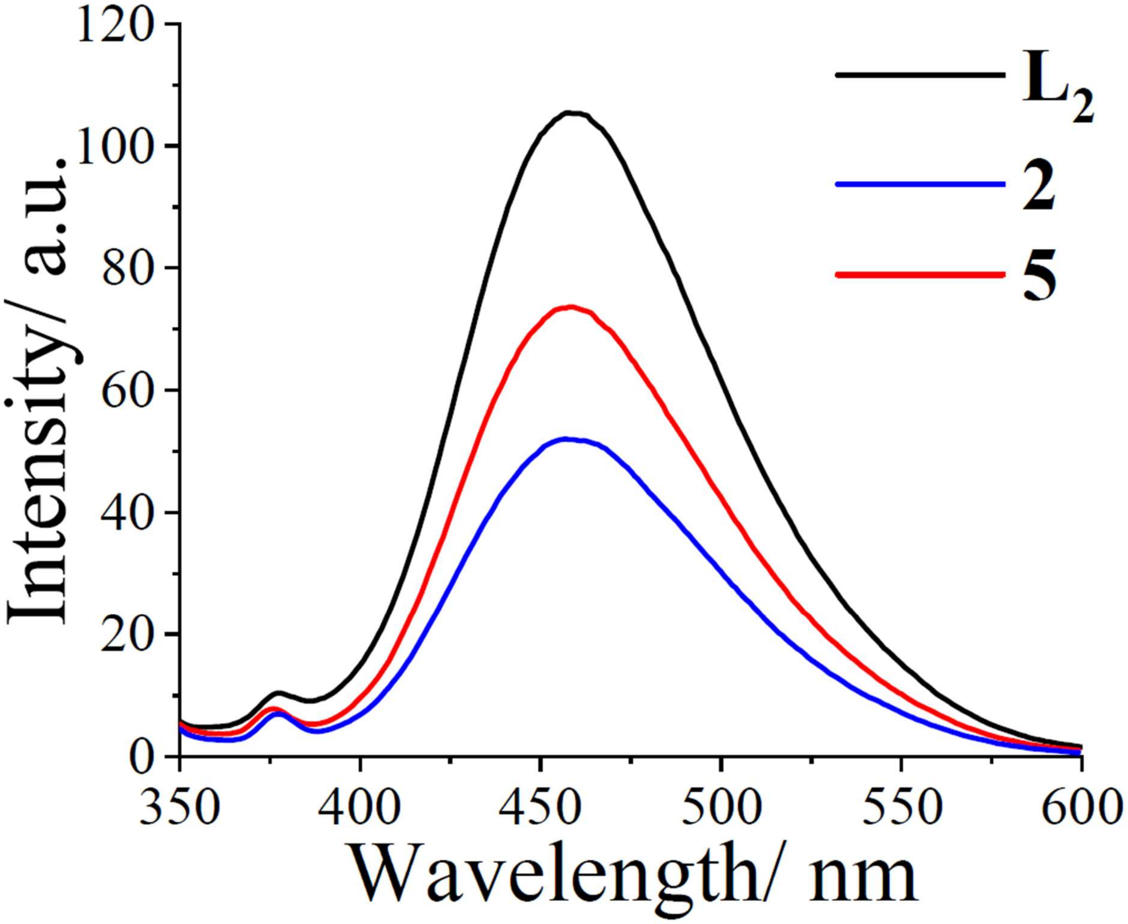

Fig. S12 Emission spectra of ligand $\mathbf{L_2}$ and the complexes **2** and **5** (10 μM) in DMSO-Tris-HCl buffer (pH 7.2) (1:4 v/v).

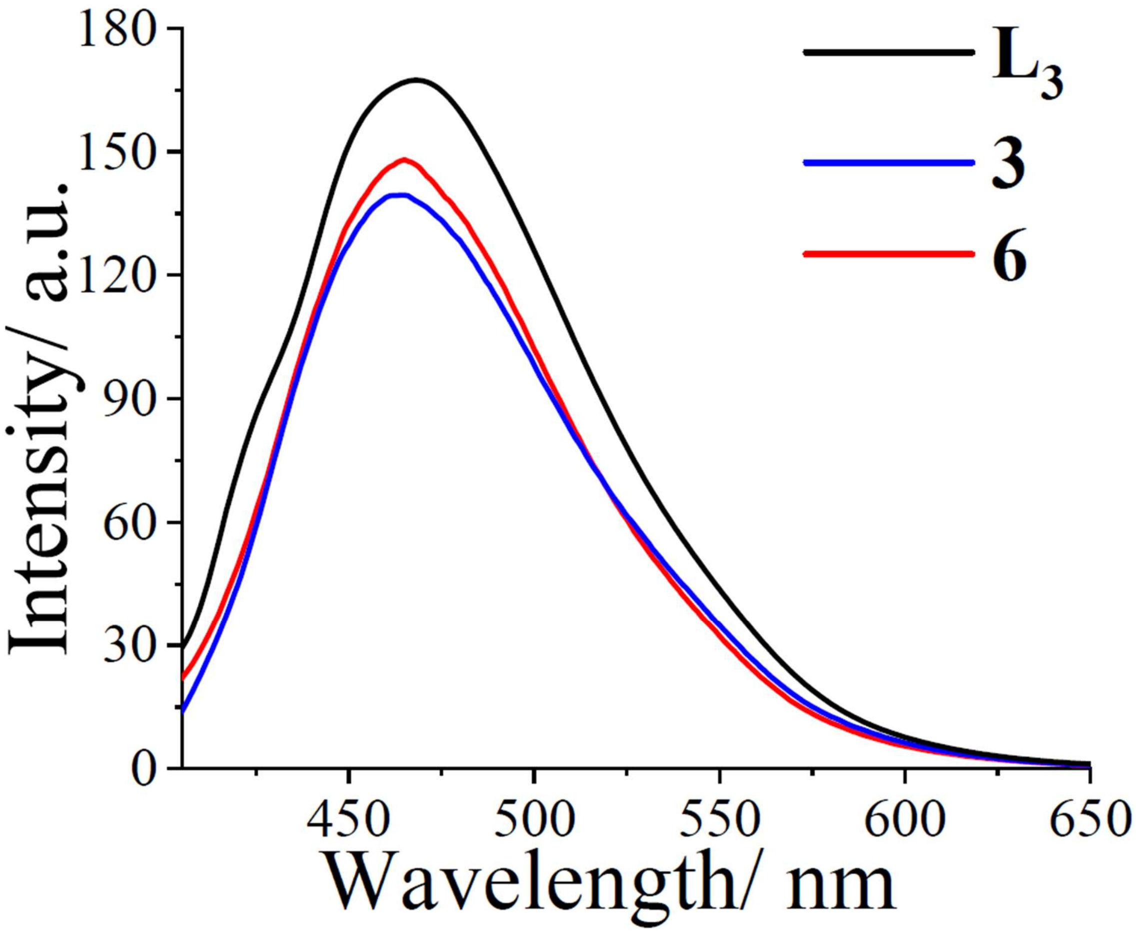

Fig. S13 Emission spectra of ligand **L**$_3$ and the complexes **3** and **6** (10 μM) in DMSO-Tris-HCl buffer (pH 7.2) (1:4 v/v).

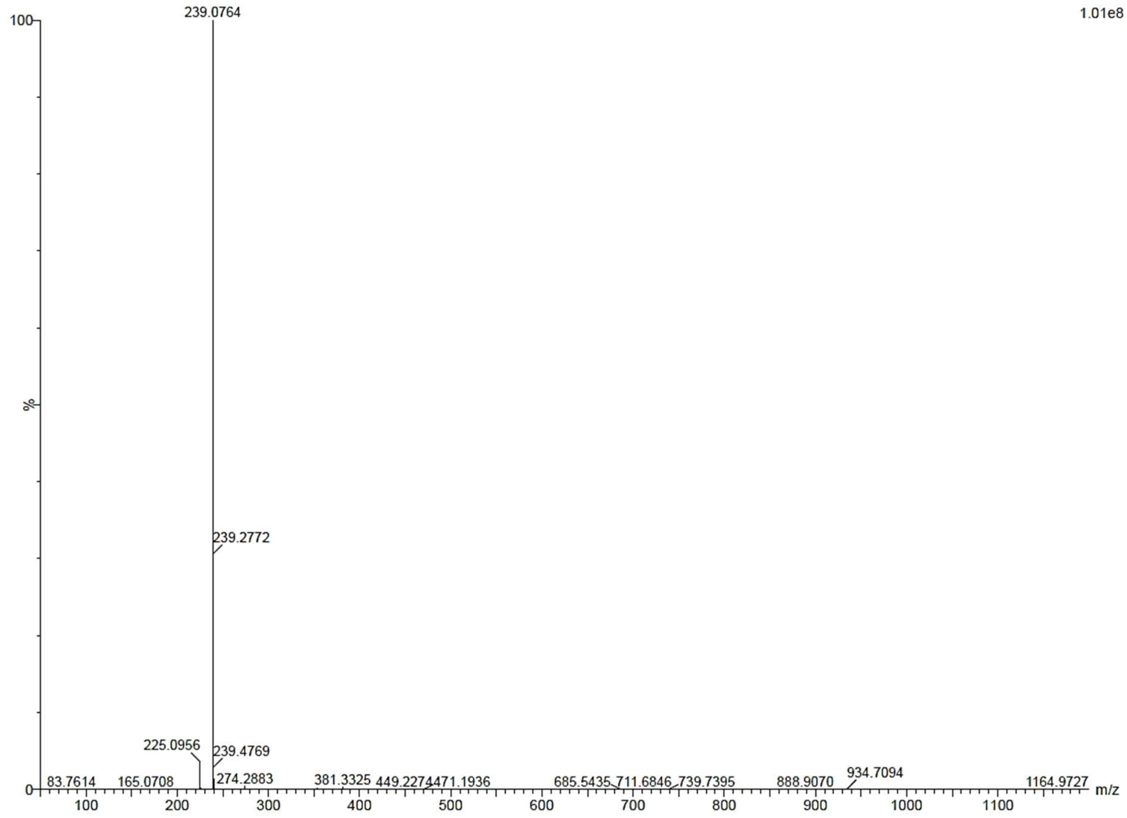

Fig. S14 ESI Mass spectrum of the ligand 3-hydroxy flavone (**L$_1$**) in methanol showing the $[M+H]^+$ peak at m/z = 239.0764 (Calcd: 239.0708).

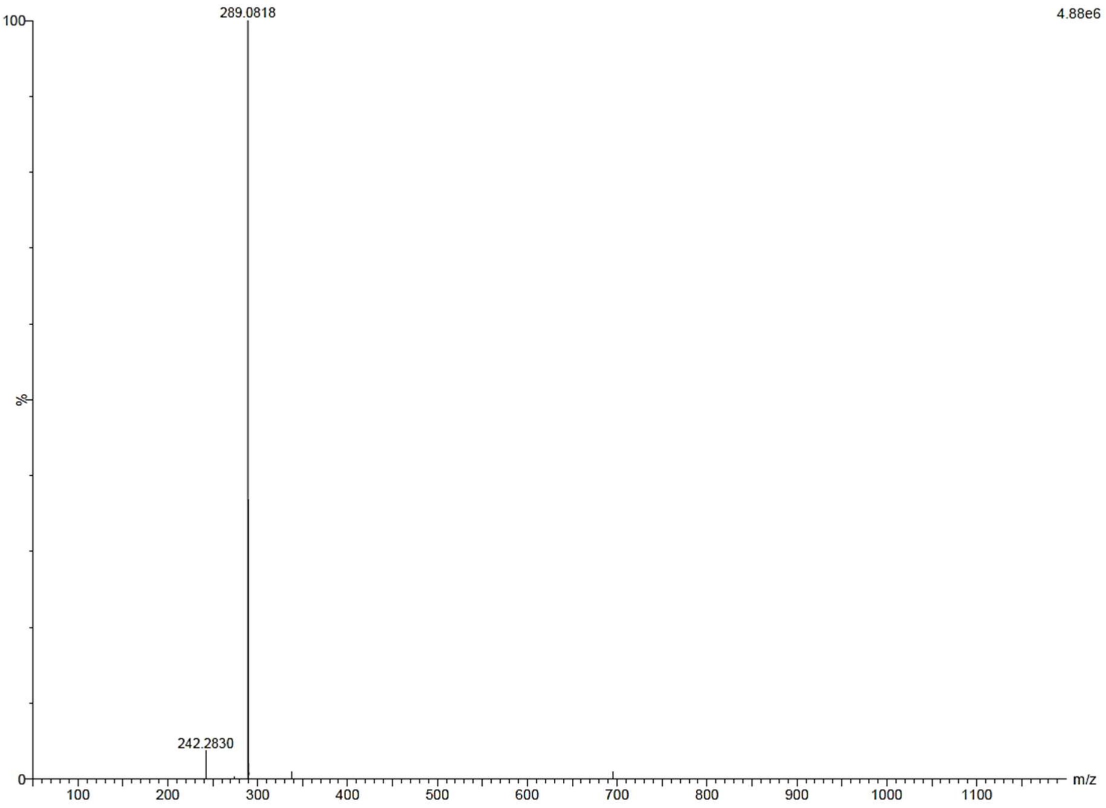

Fig. S15 ESI Mass spectrum of the ligand naphthyl flavone (**L**$_2$) in methanol showing the $[M+H]^+$ peak at m/z = 289.0818 (Calcd: 289.0865).

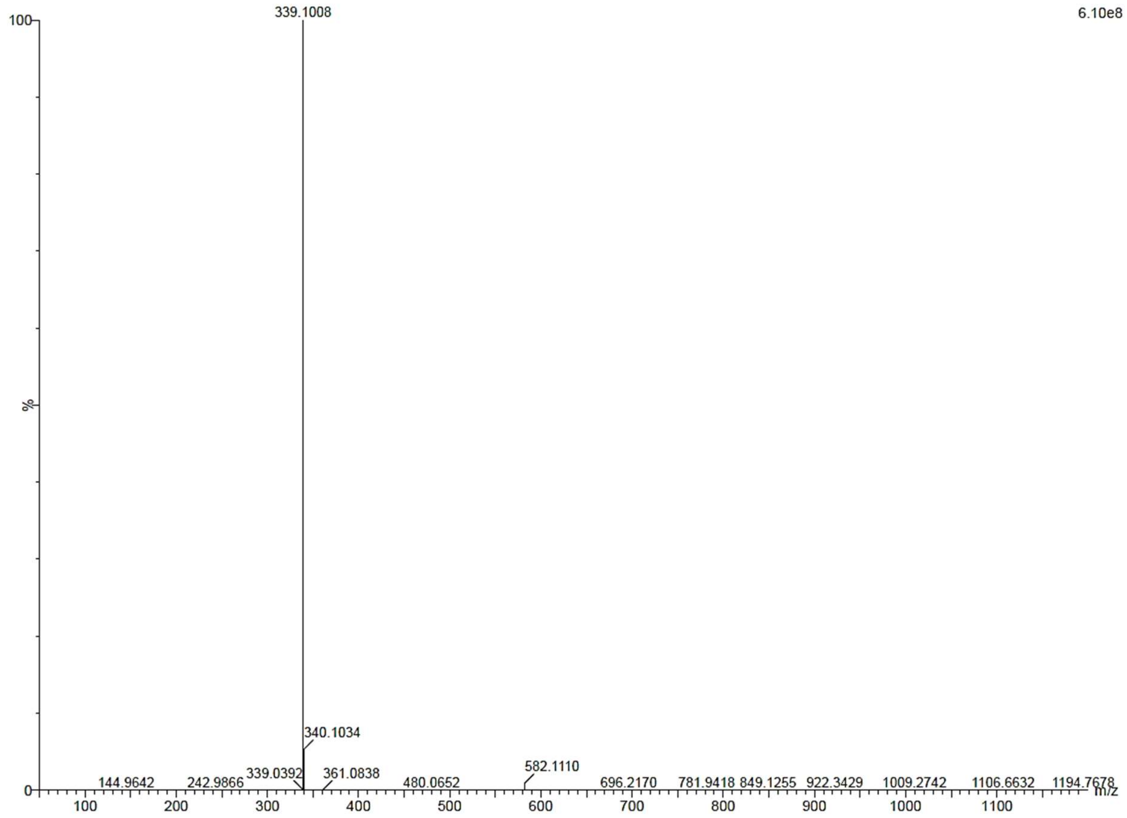

Fig. S16 ESI Mass spectrum of the ligand anthracenyl flavone (**L**$_3$) in methanol showing the $[M+H]^+$ peak at m/z = 339.1008 (Calcd: 339.1021).

.

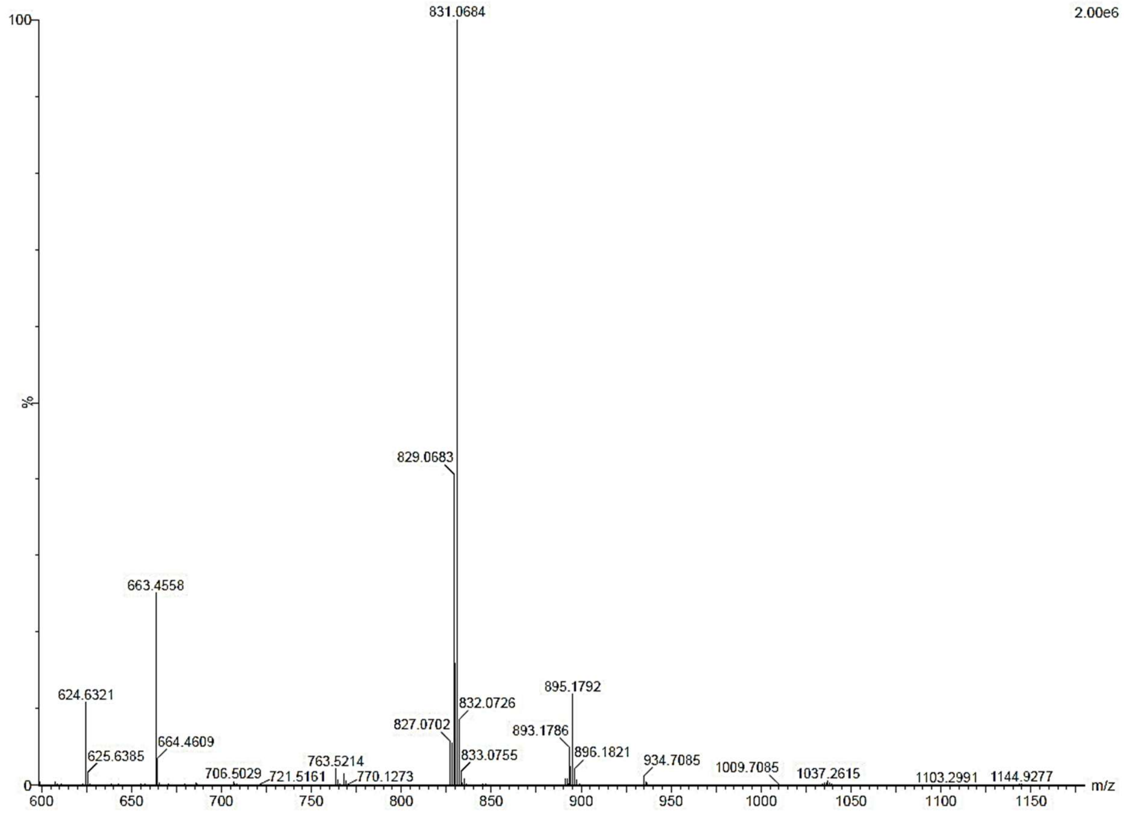

Fig. S17 ESI Mass spectrum of the complex **1** in methanol showing the $[M]^+$ peak at m/z = 831.0684 (Calcd: 831.0677).

.

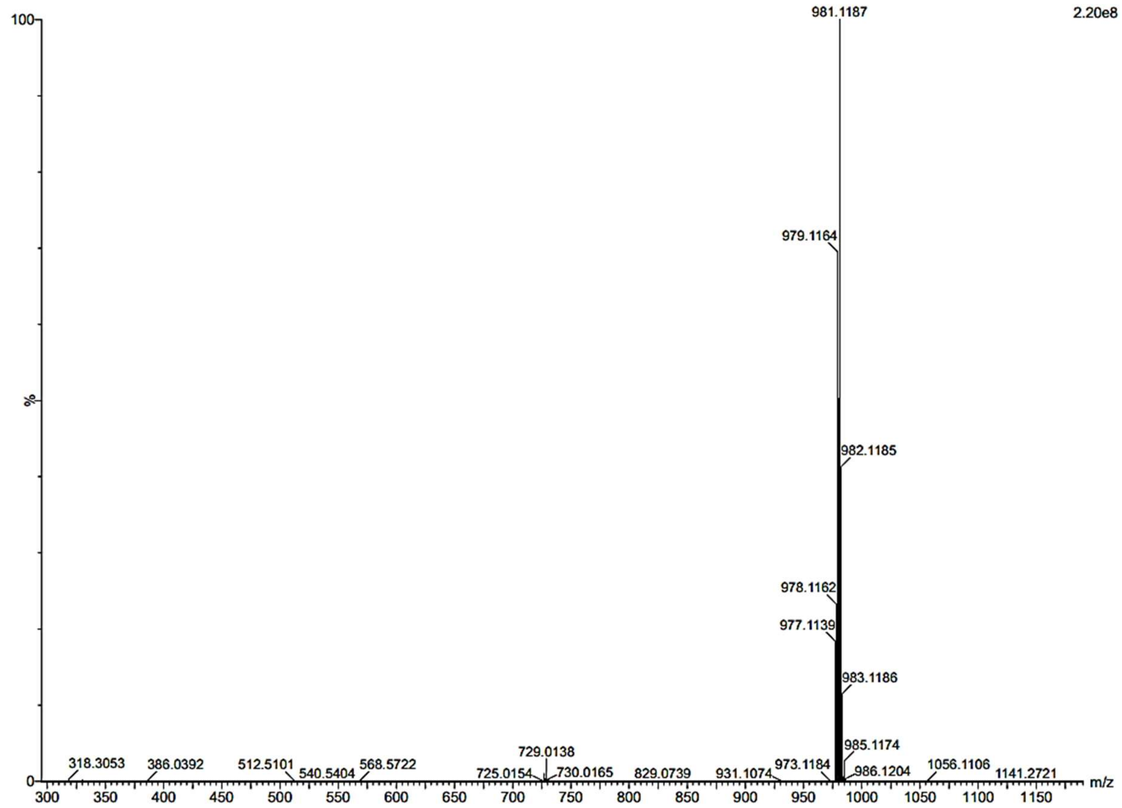

Fig. S18 ESI Mass spectrum of the complex **2** in methanol showing the $[M]^+$ peak at m/z = 981.1187 (Calcd: 981.1144).

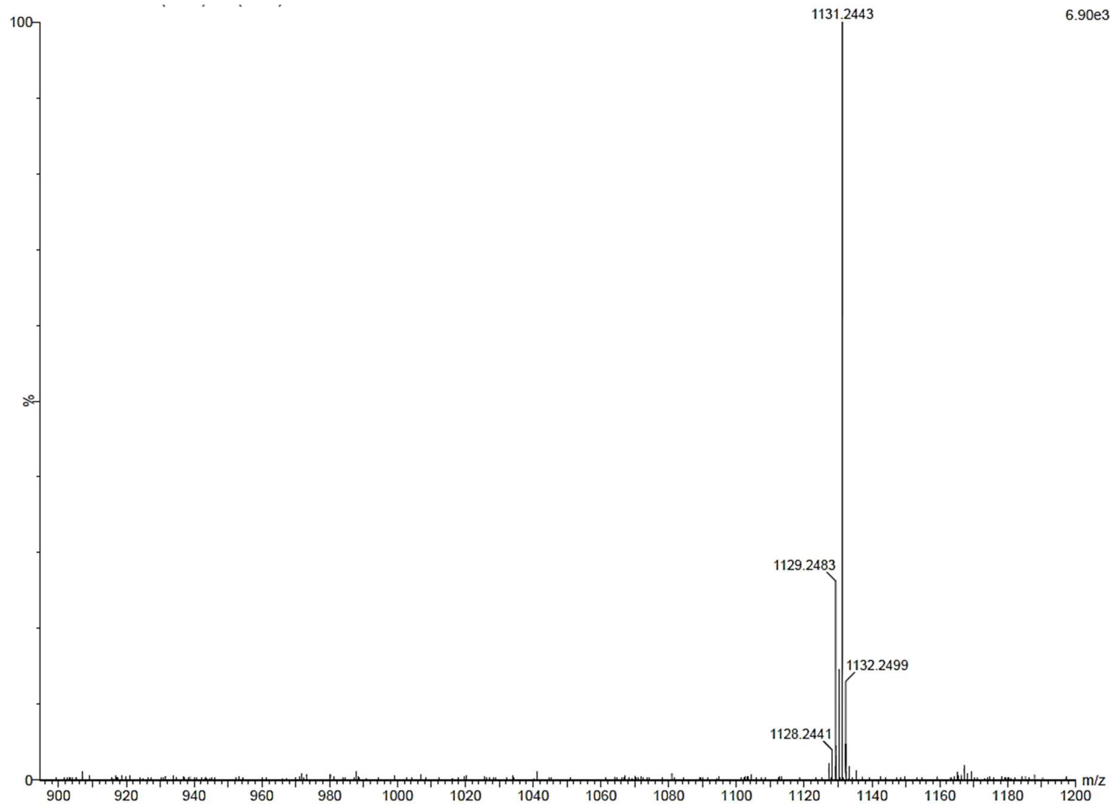

Fig. S19 ESI Mass spectrum of the complex **3** in methanol showing the $[M]^+$ peak at m/z = 1131.2443 (Calcd: 1131.1616).

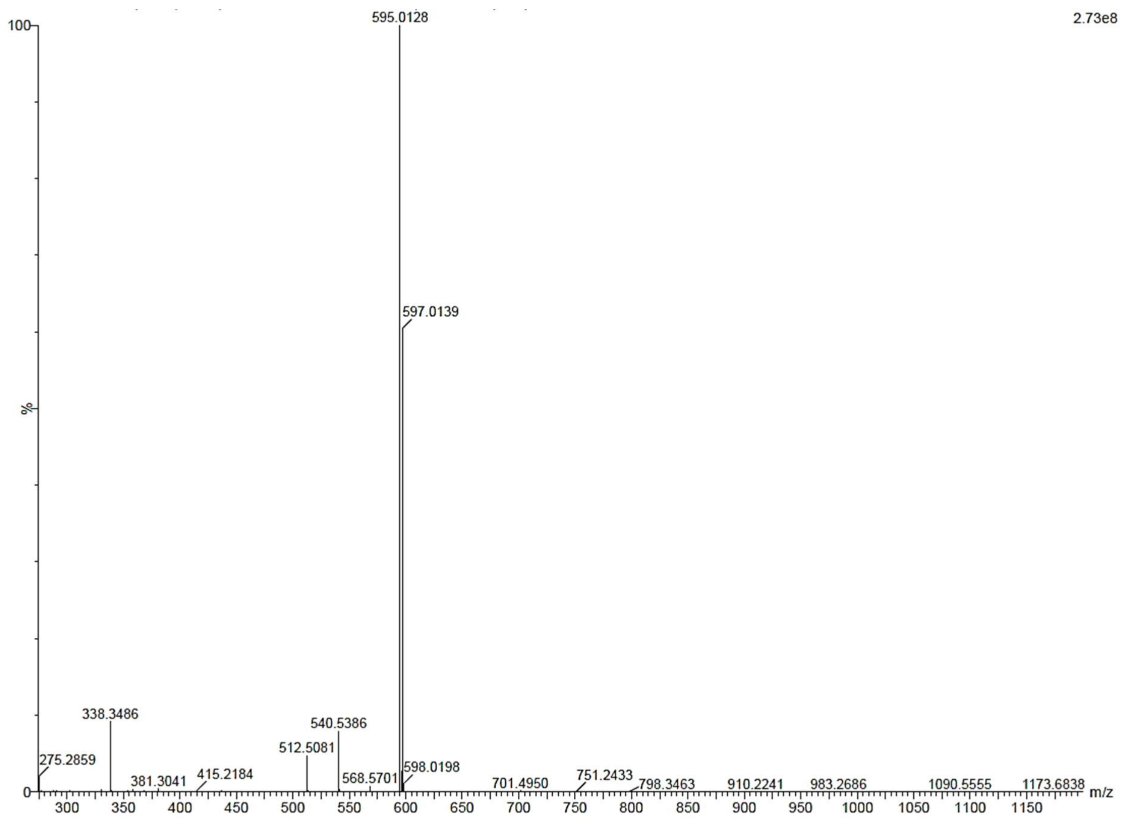

Fig. S20 ESI Mass spectrum of the complex **4** in methanol showing the $[M\text{-}Cl]^+$ peak at m/z = 595.0128 (Calcd: 595.0136).

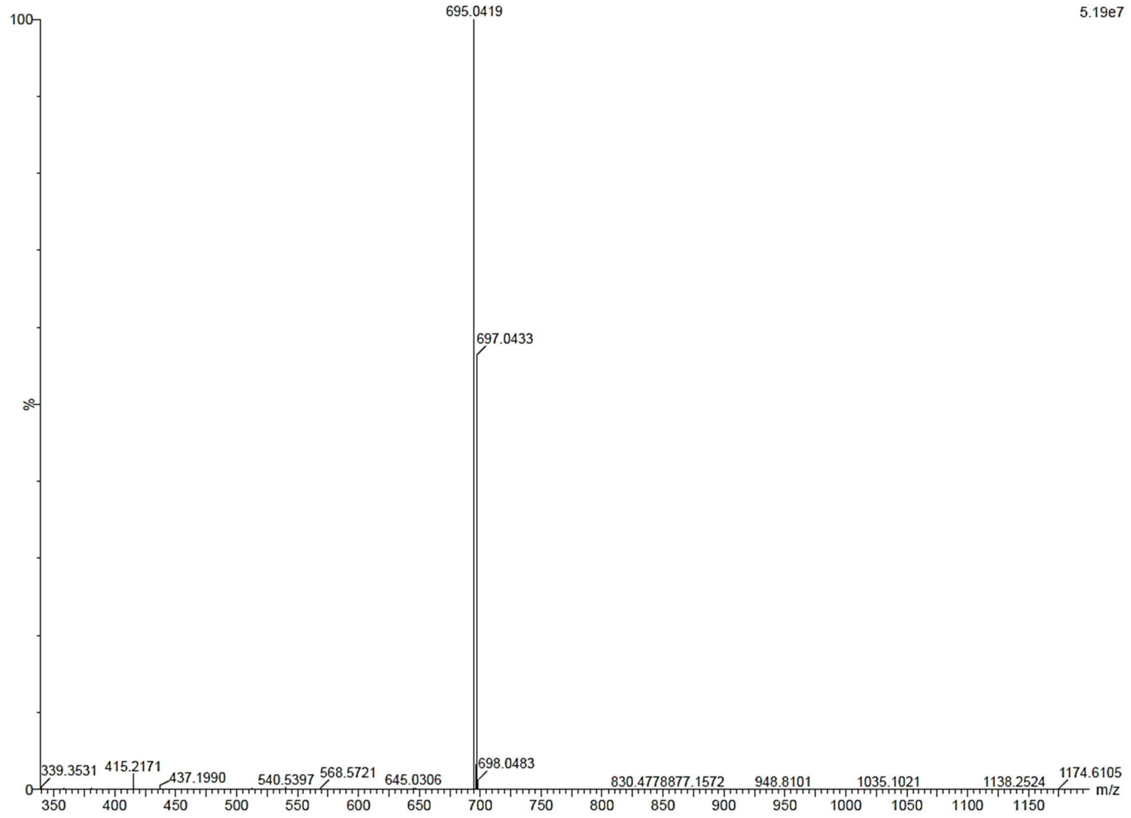

Fig. S21 ESI Mass spectrum of the complex **5** in methanol showing the $[M\text{-}Cl]^+$ peak at m/z = 695.0419 (Calcd: 695.0449).

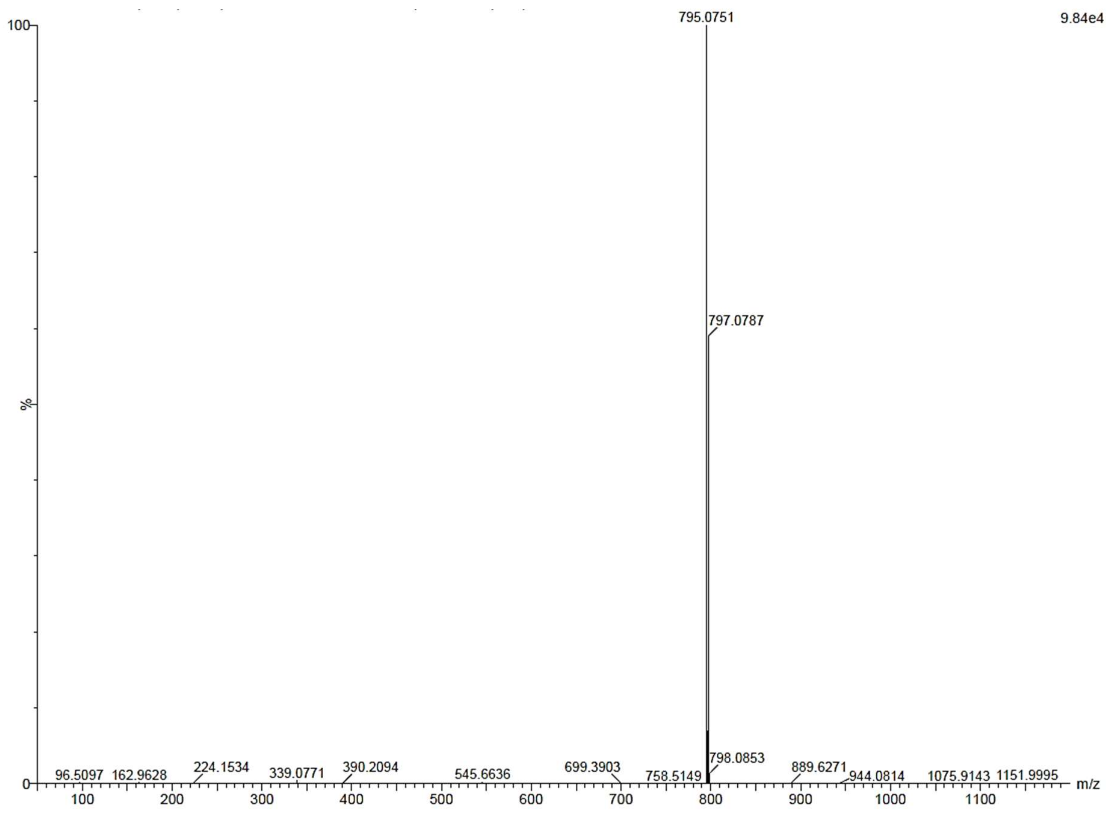

Fig. S22 ESI Mass spectrum of the complex **6** in methanol showing the $[M\text{-}Cl]^+$ peak at m/z = 795.0751(Calcd: 795.0762).

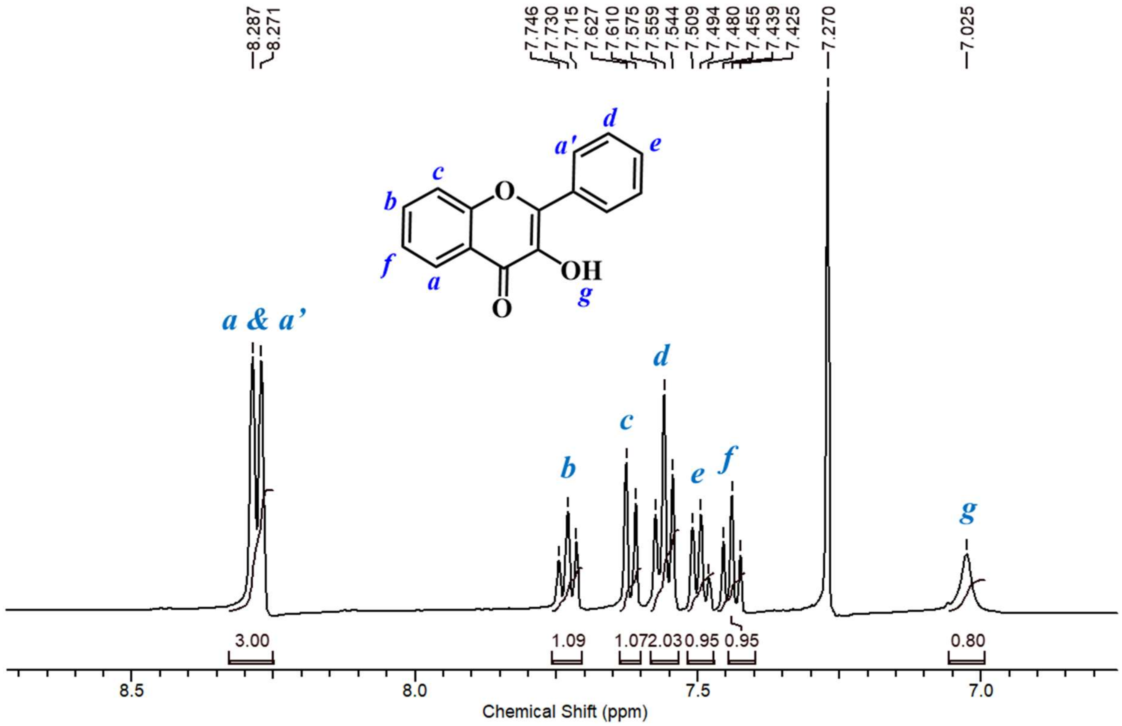

Fig. S23 ^{1}H NMR spectrum of 3-hydroxy flavone (**L_1**) in $CDCl_3$.

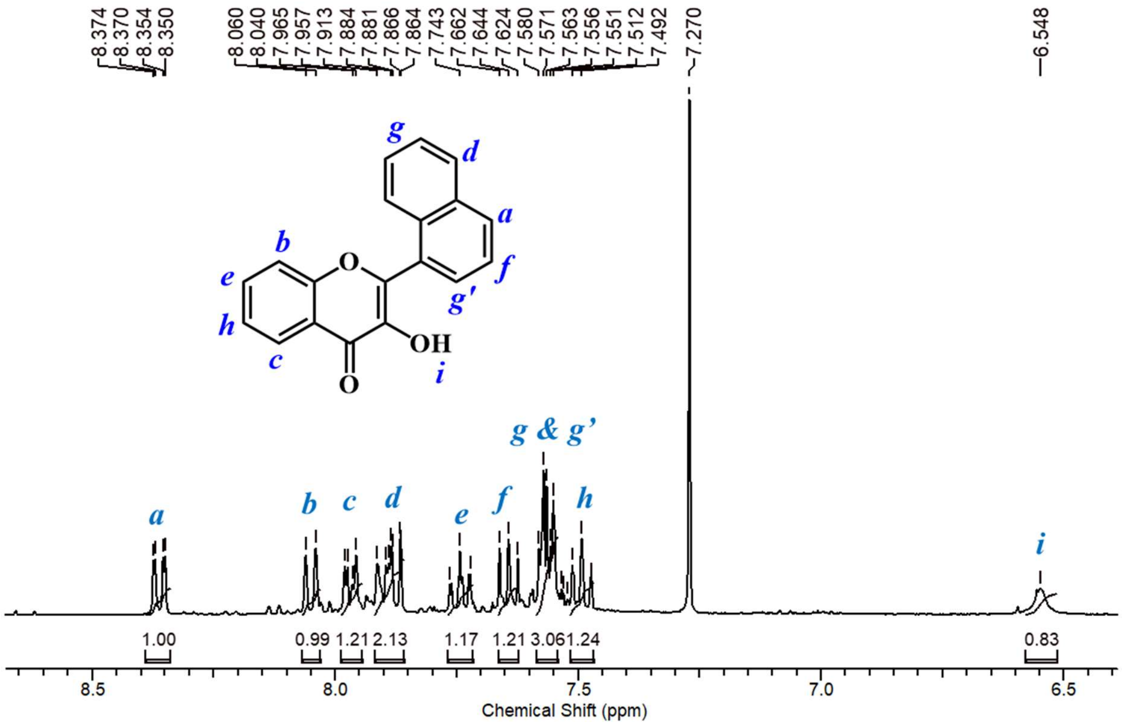

Fig. S24 ^{1}H NMR spectrum of naphthyl flavone (**L$_2$**) in $CDCl_3$.

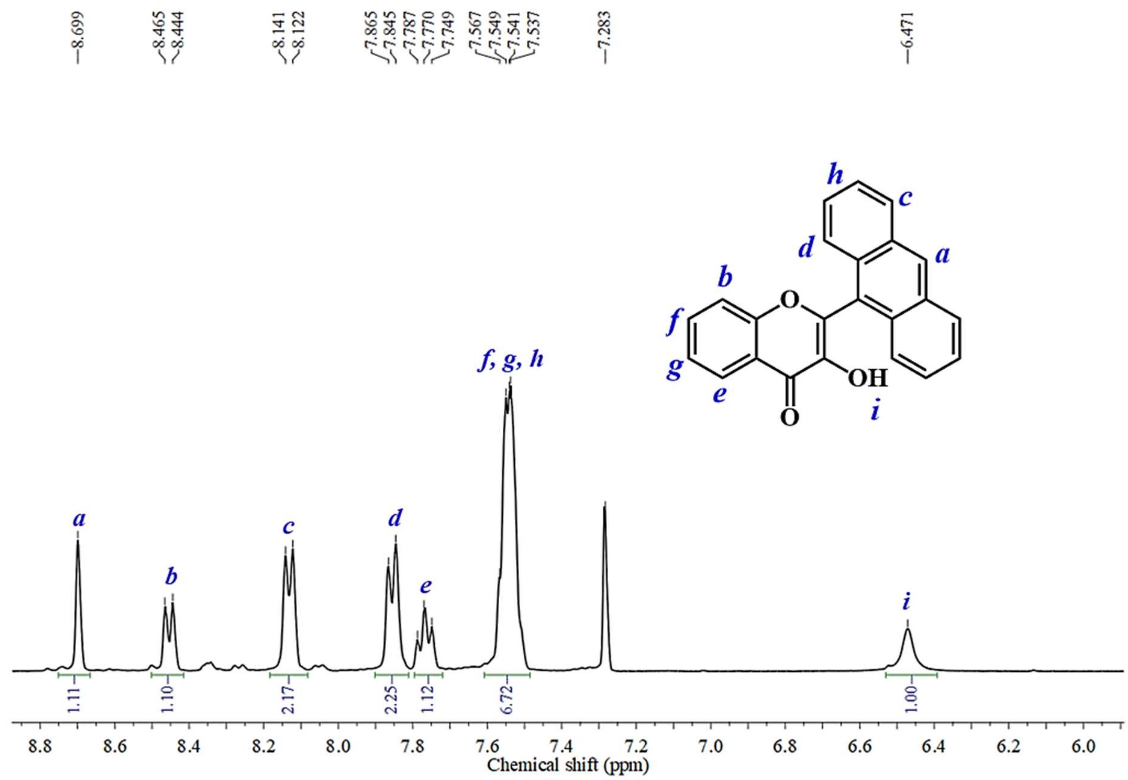

Fig. S25 ^{1}H NMR spectrum of anthracenyl flavone (**L$_3$**) in $CDCl_3$.

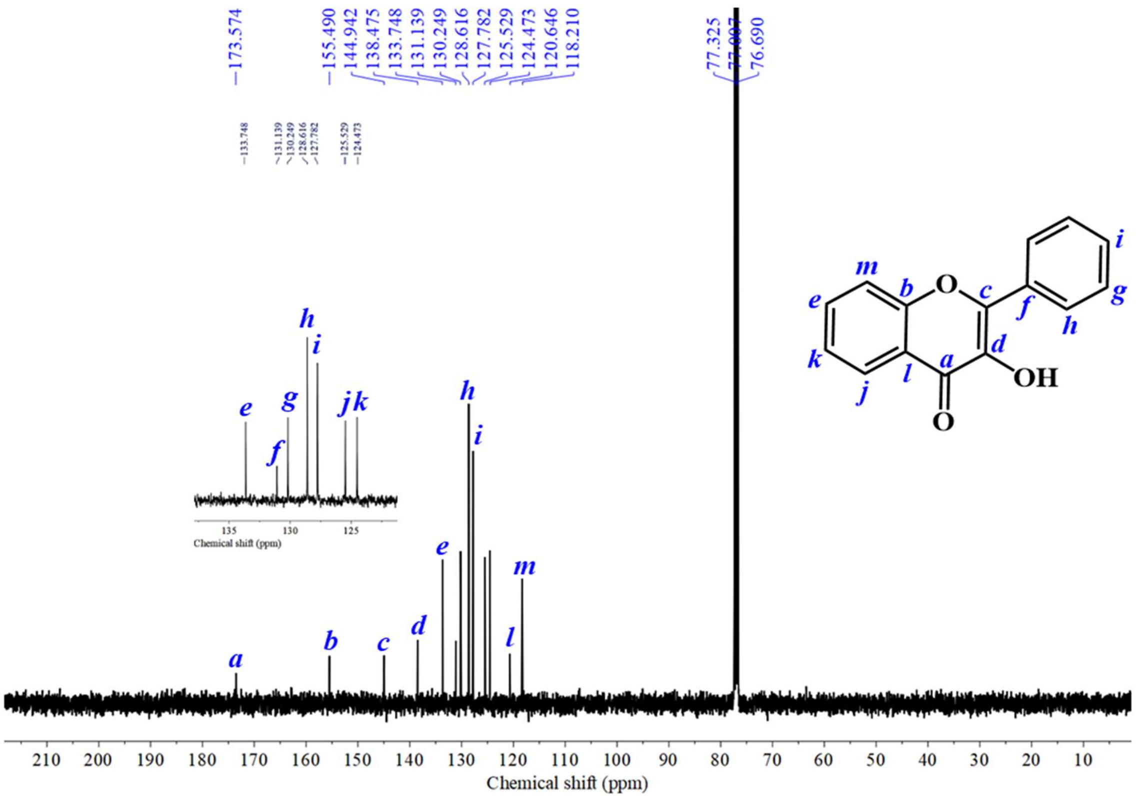

Fig. S26 ^{13}C NMR spectrum of 3-hydroxy flavone (**L_1**) in $CDCl_3$.

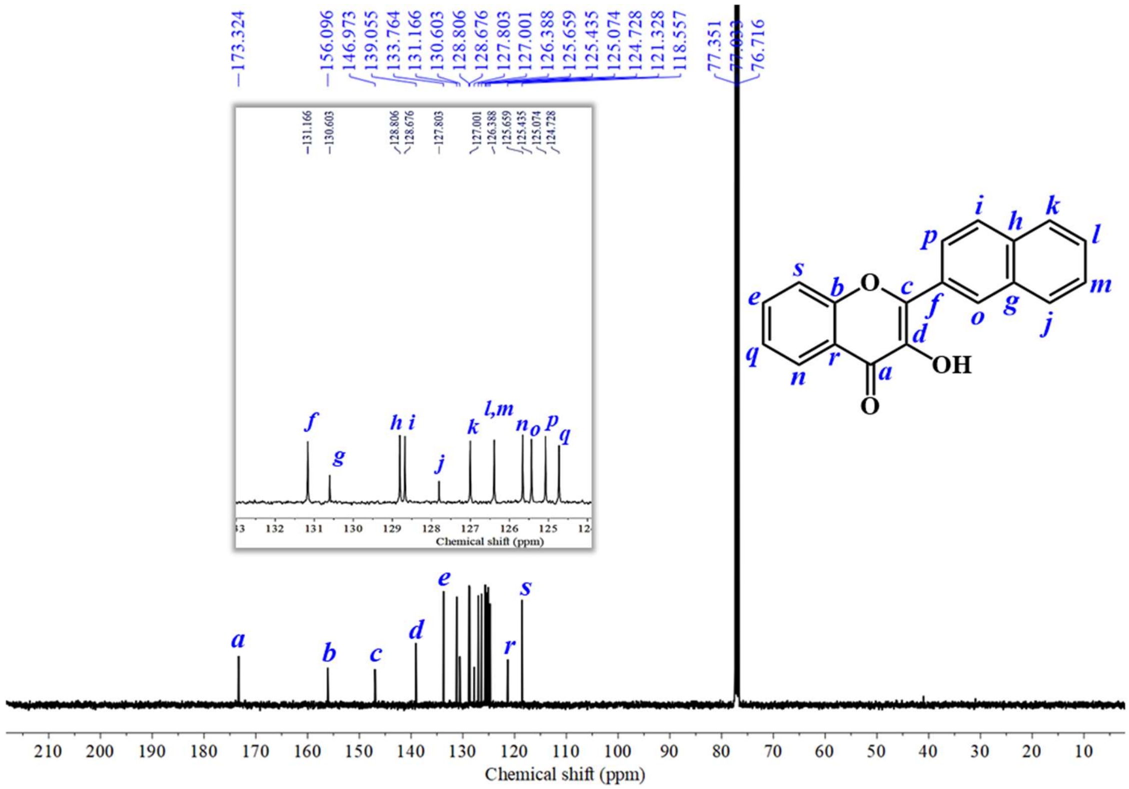

Fig. S27 ^{13}C NMR spectrum of naphthyl flavone (**L$_2$**) in $CDCl_3$.

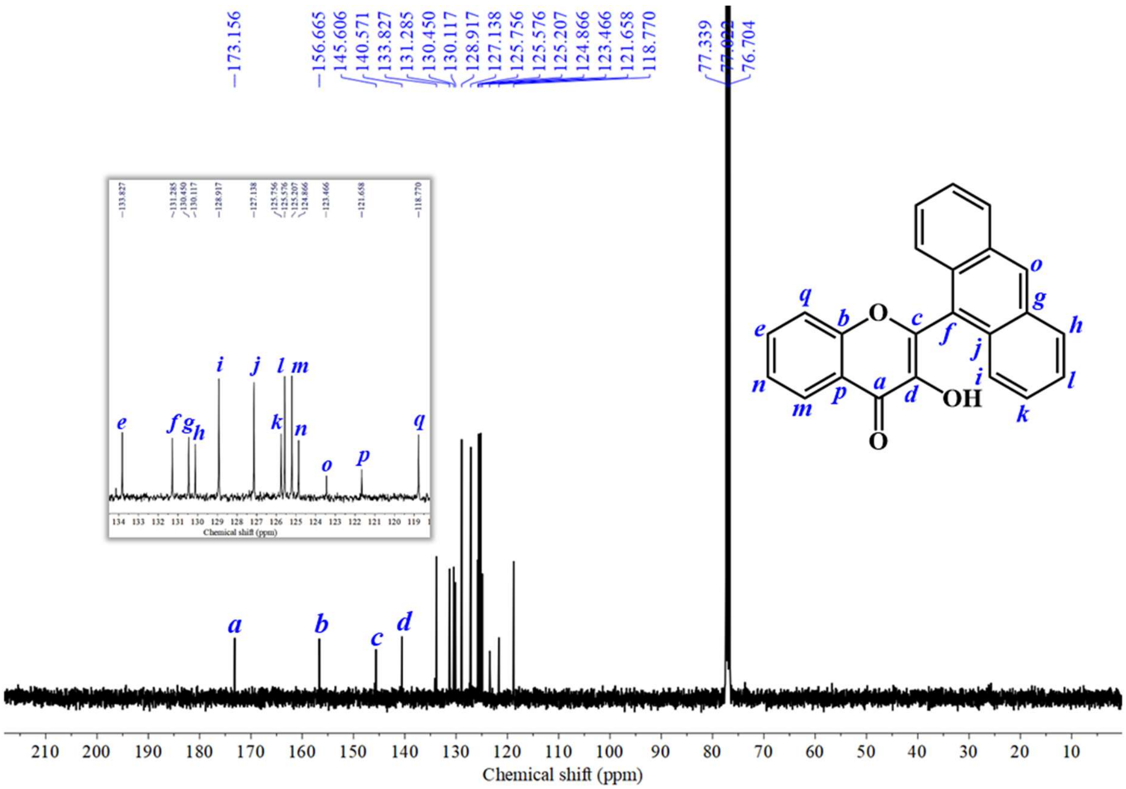

Fig. S28 ^{13}C NMR spectrum of anthracenyl flavone (**L3**) in $CDCl_3$.

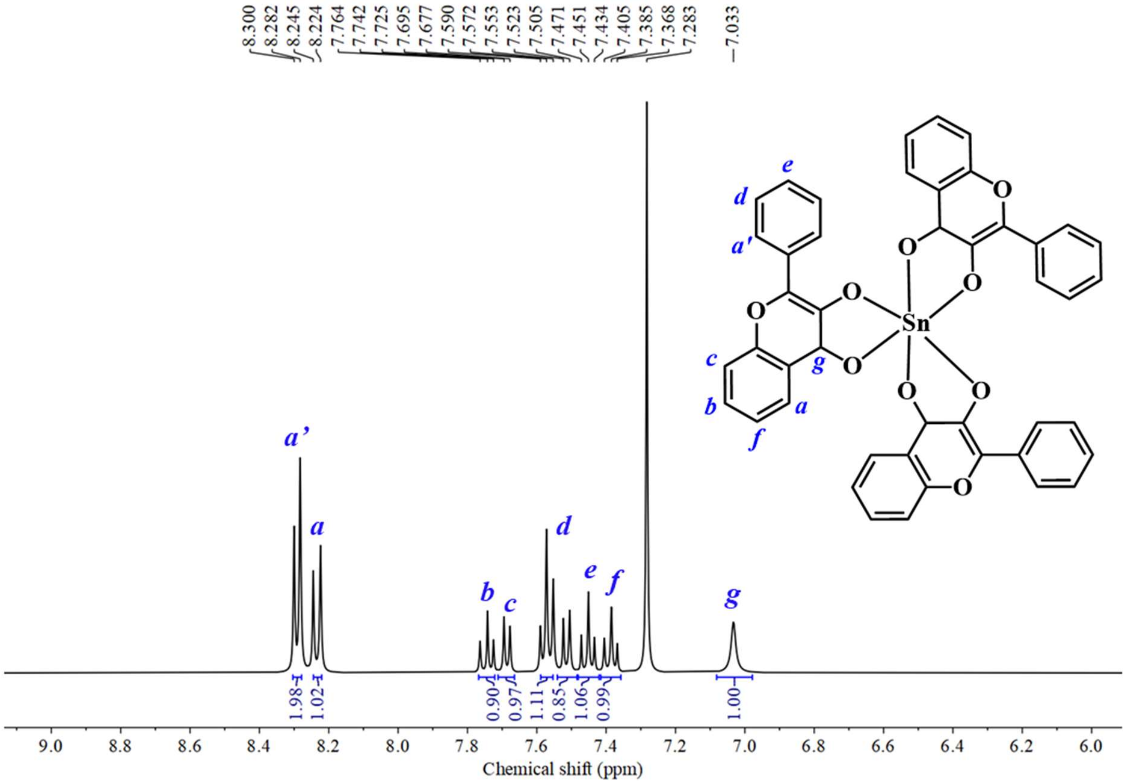

Fig. S29 ^{1}H NMR spectrum of complex **1** in $CDCl_3$.

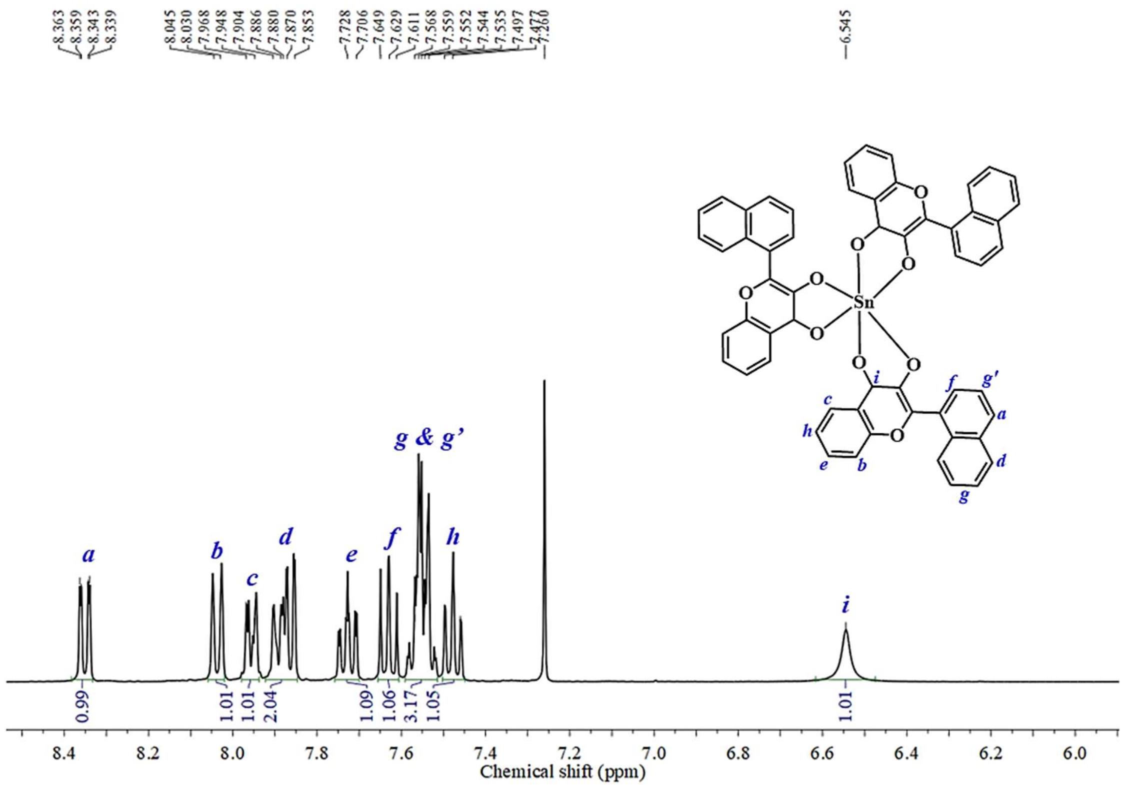

Fig. S30 ^{1}H NMR spectrum of complex **2** in $CDCl_3$.

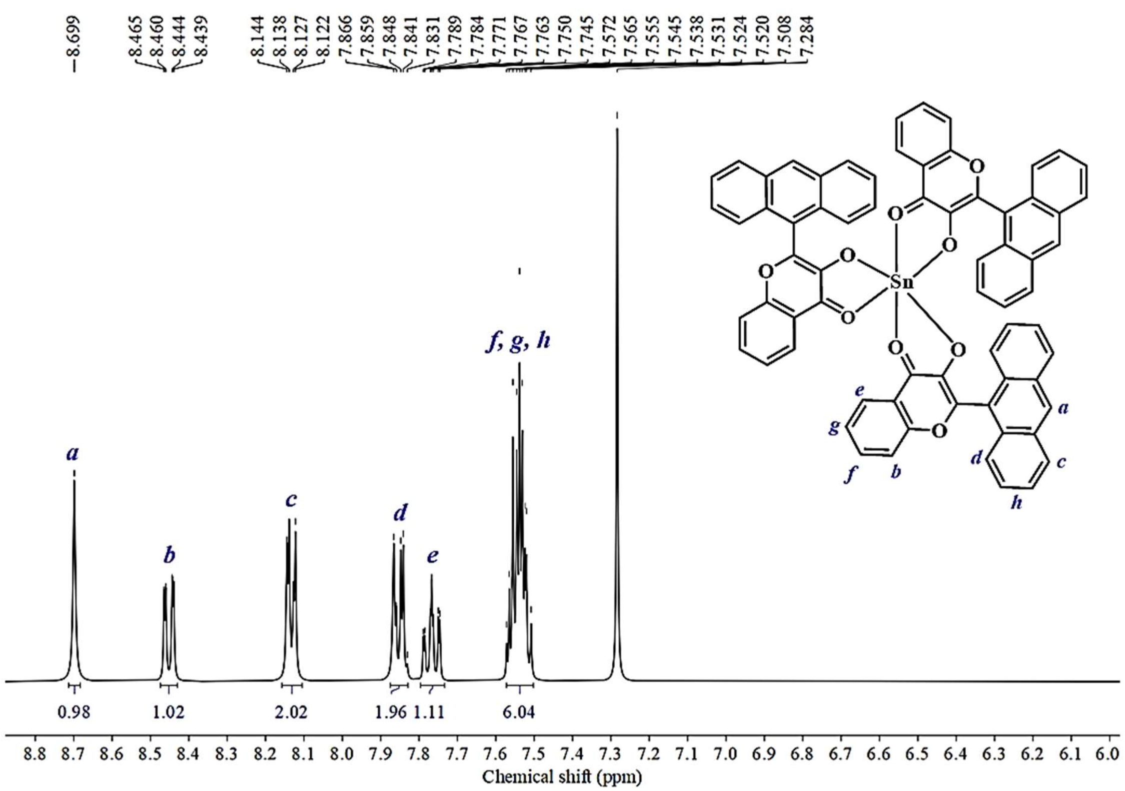

Fig. S31 ^{1}H NMR spectrum of complex **3** in $CDCl_3$.

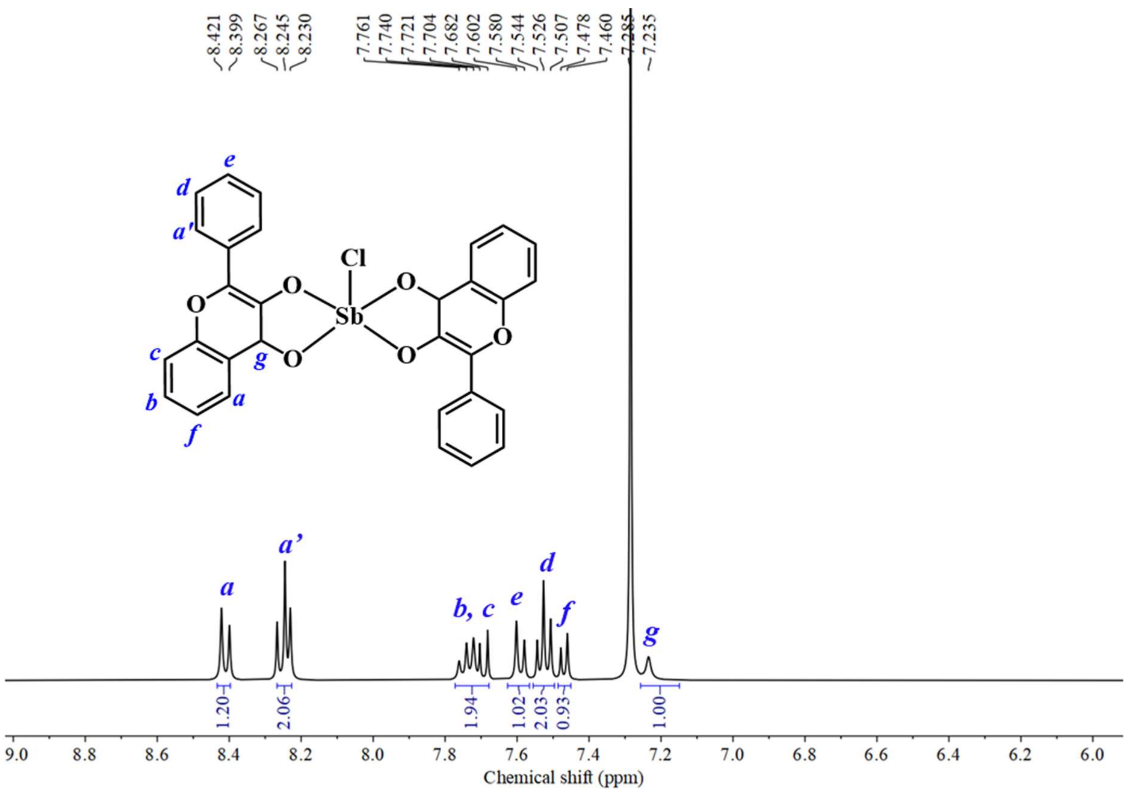

Fig. S32 ^{1}H NMR spectrum of complex **4** in $CDCl_3$.

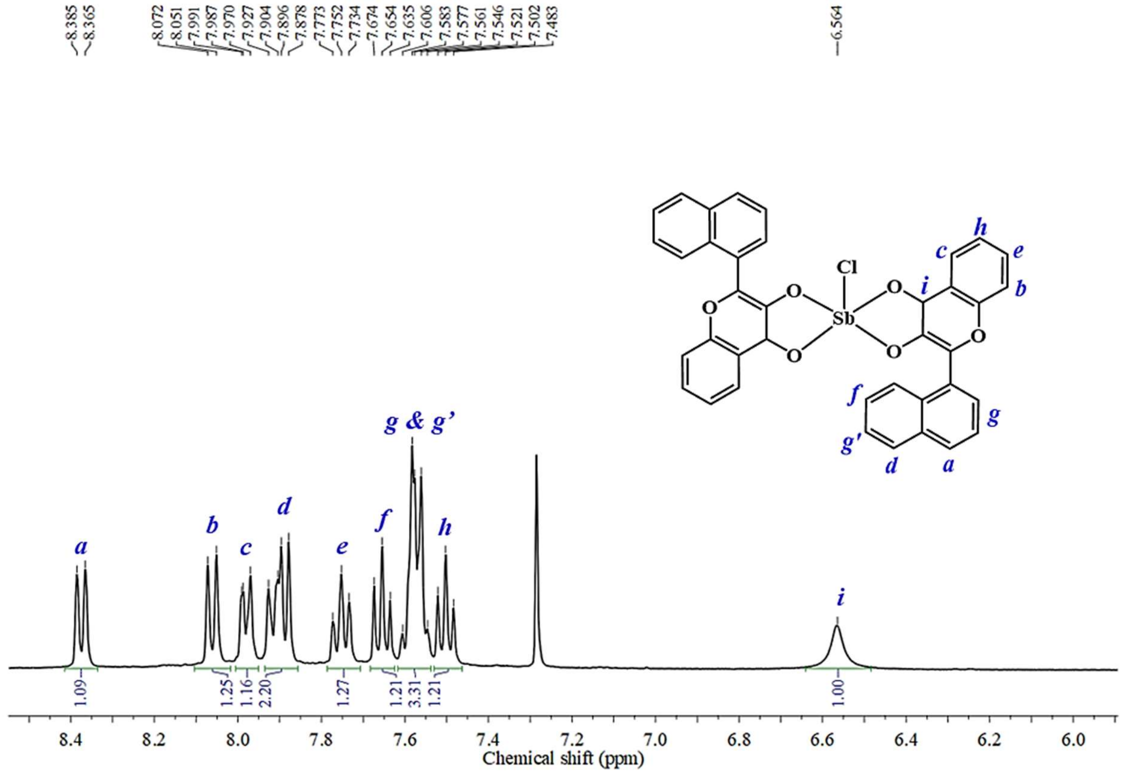

Fig. S33 ^{1}H NMR spectrum of complex **5** in $CDCl_3$.

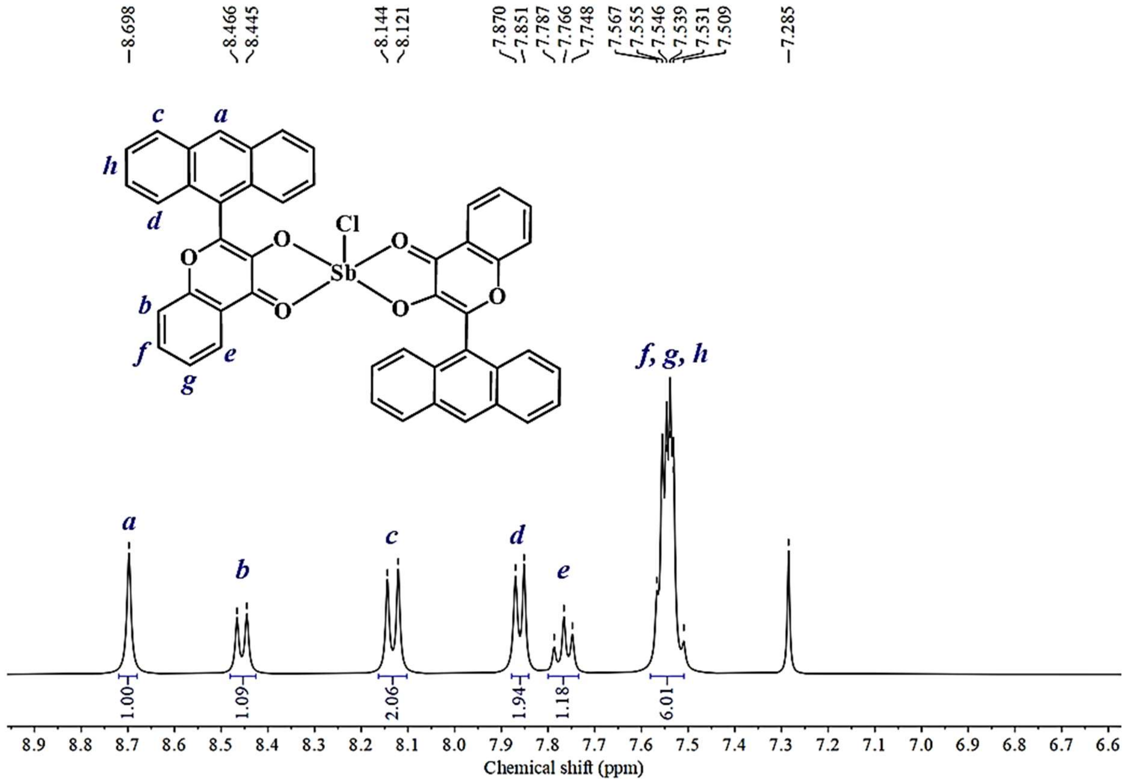

Fig. S34 ^{1}H NMR spectrum of complex **6** in $CDCl_3$.

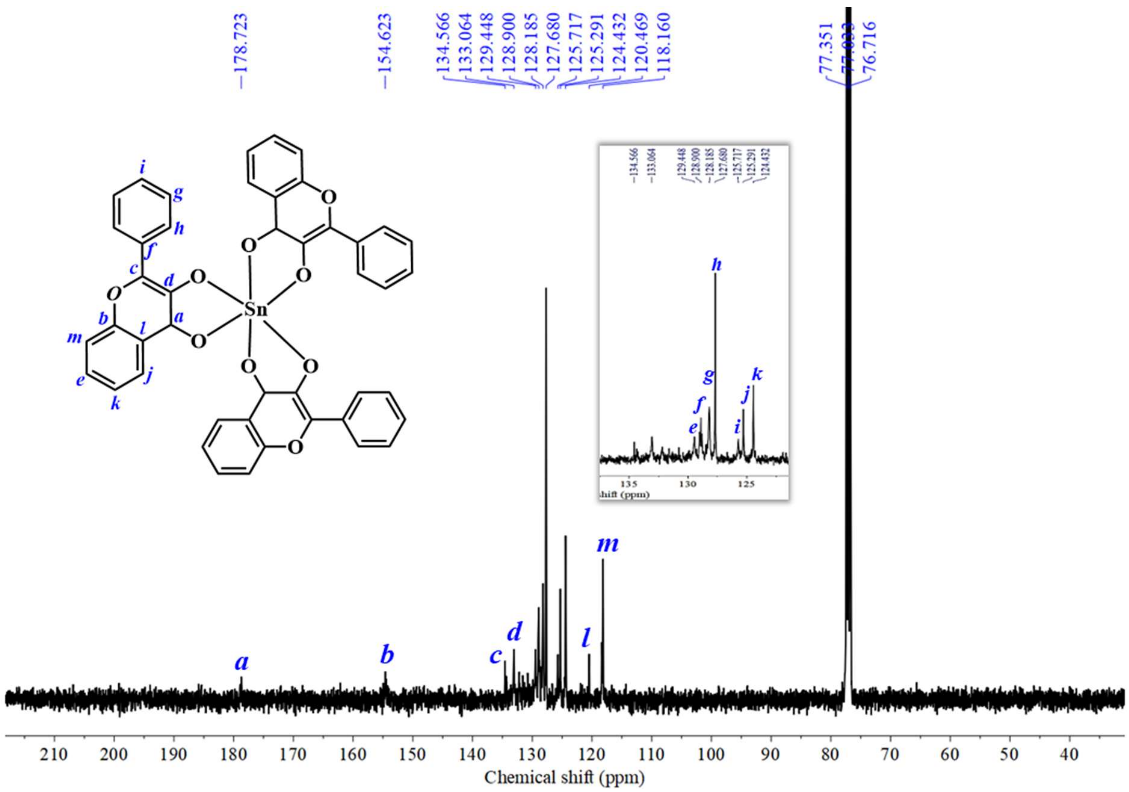

Fig. S35 ^{13}C NMR spectrum of complex **1** in $CDCl_3$.

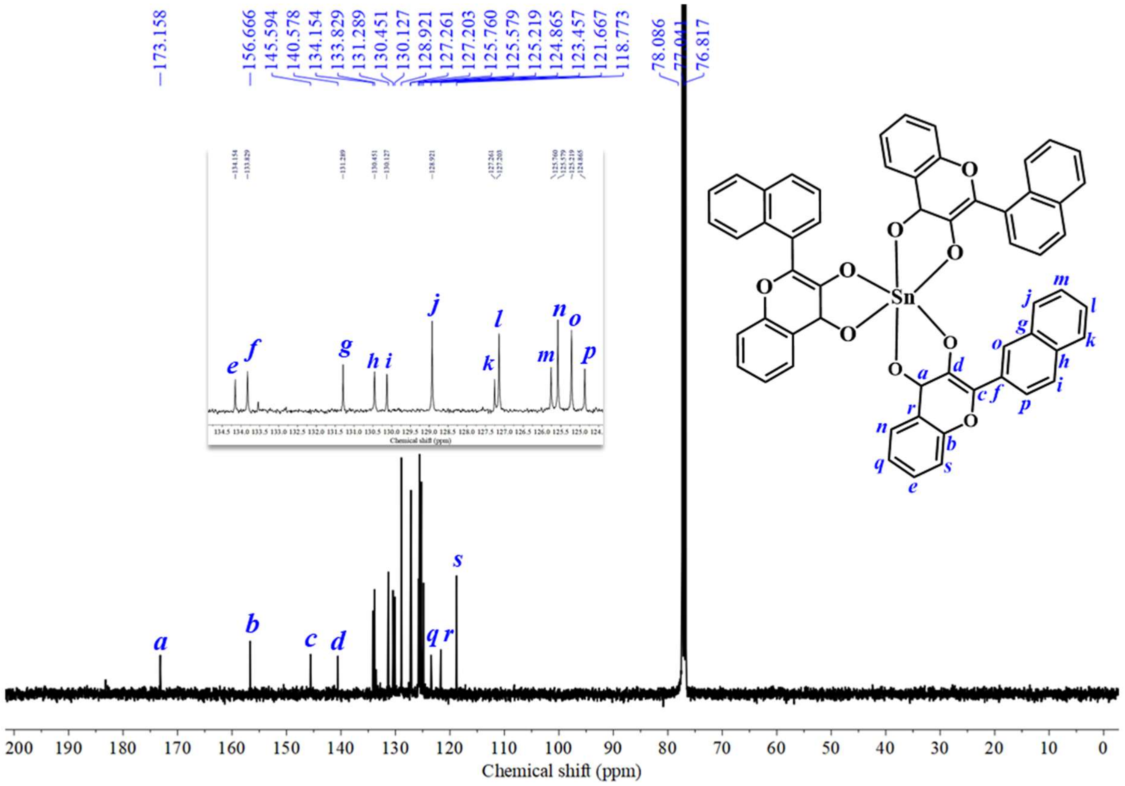

Fig. S36 ^{13}C NMR spectrum of complex **2** in $CDCl_3$.

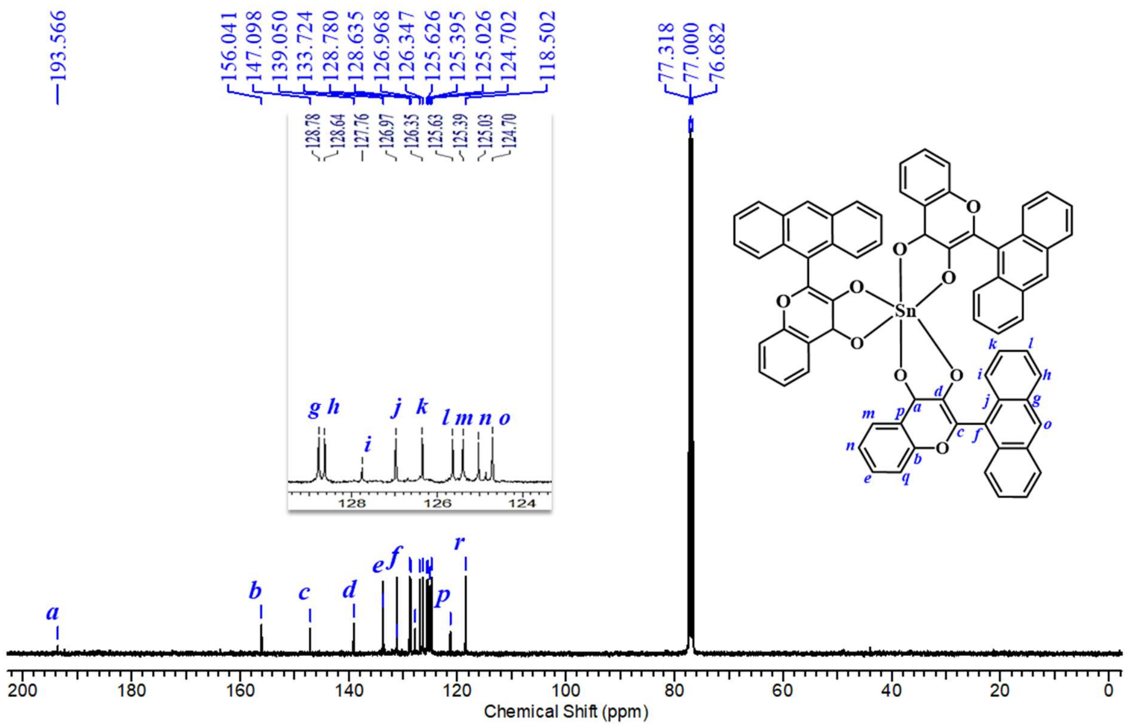

Fig. S37 ^{13}C NMR spectrum of complex **3** in $CDCl_3$.

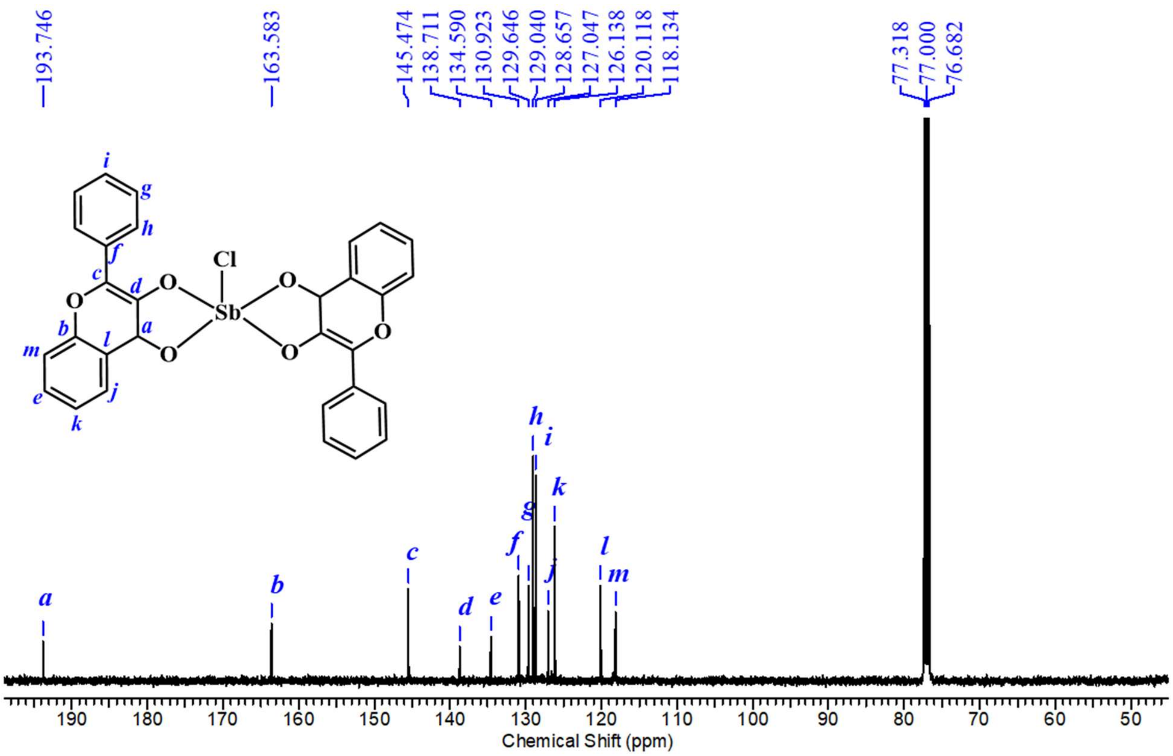

Fig. S38 ^{13}C NMR spectrum of complex **4** in $CDCl_3$.

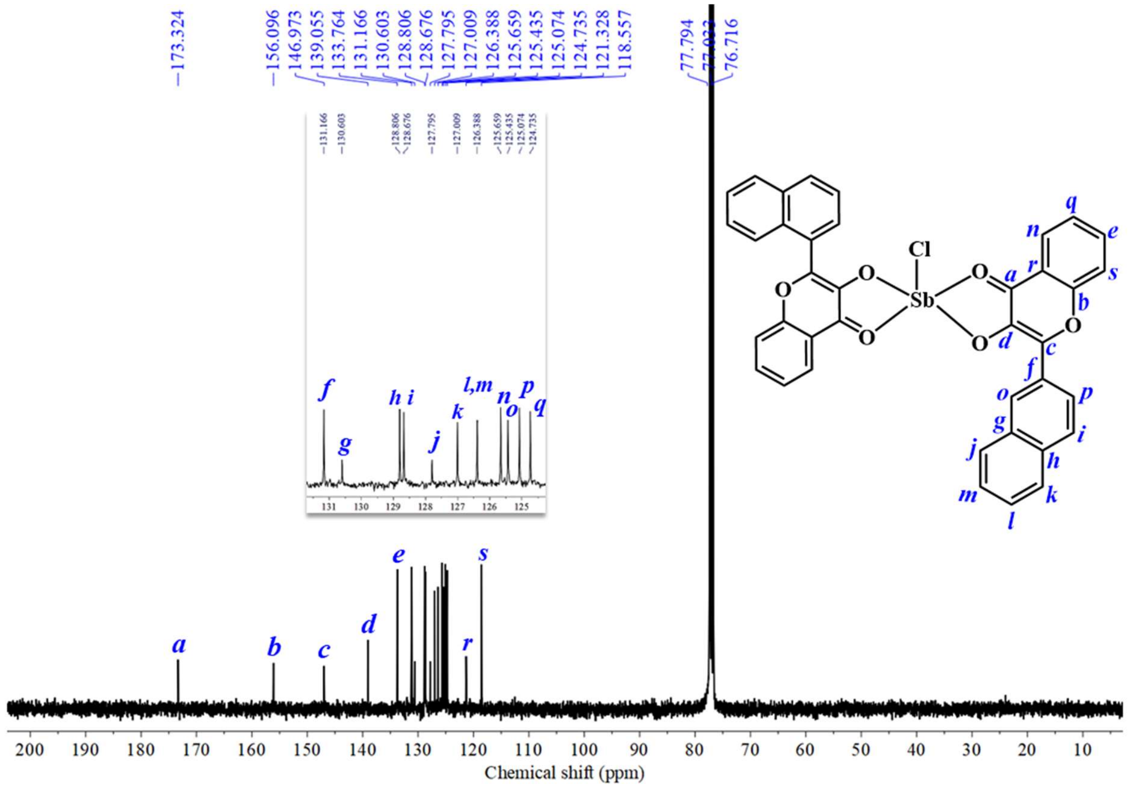

Fig. S39 ^{13}C NMR spectrum of complex **5** in $CDCl_3$.

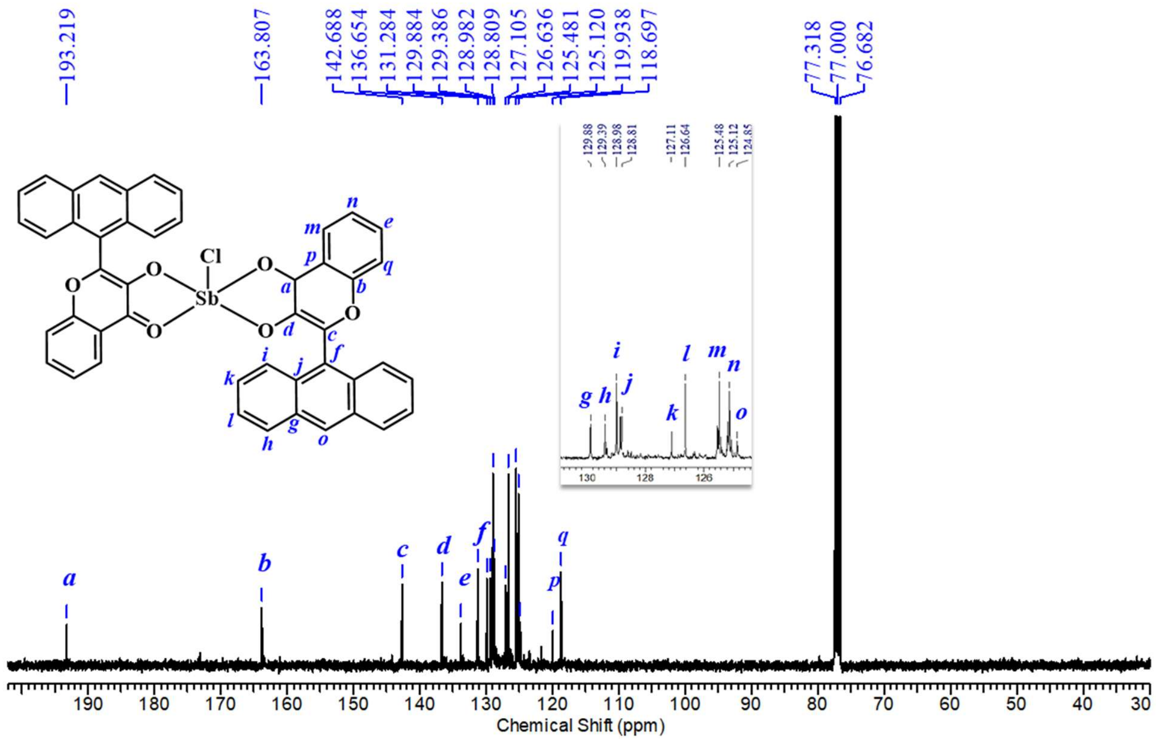

Fig. S40 ^{13}C NMR spectrum of complex **6** in $CDCl_3$.

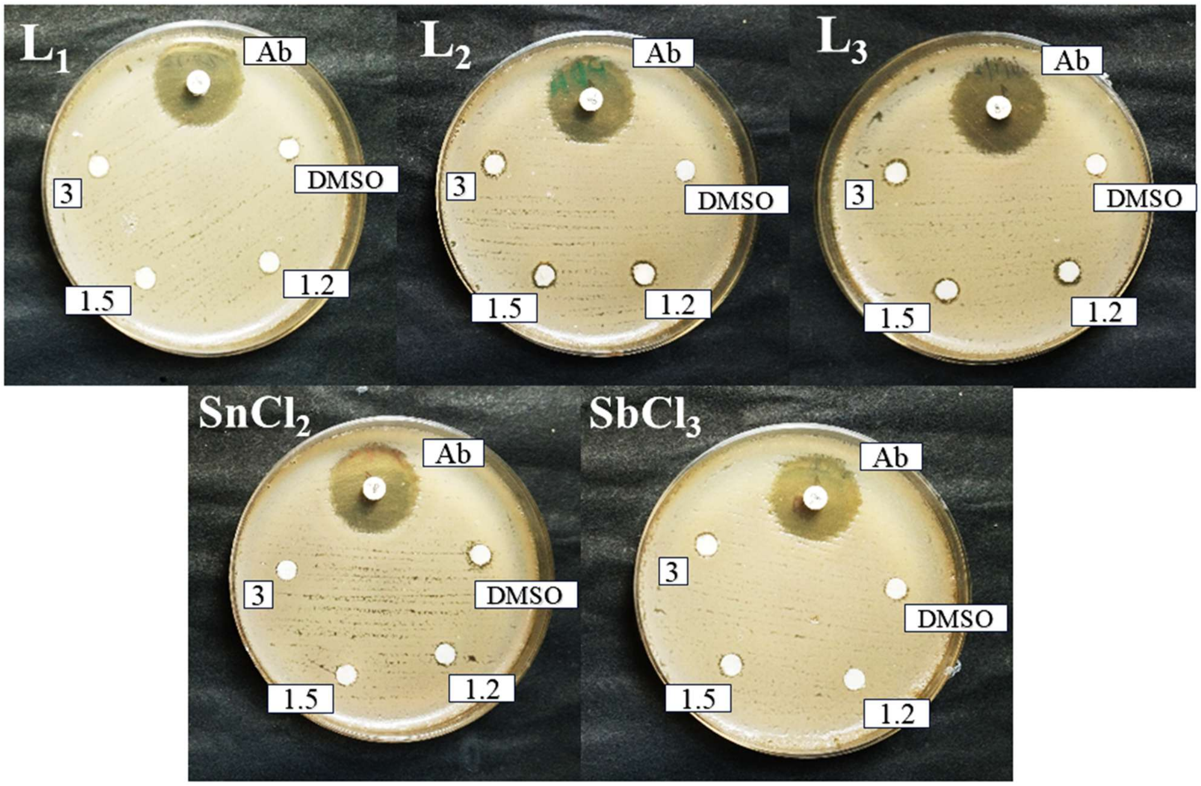

Fig. S41 Zone of inhibition of the ligands (**L_1-L_3**) and metal salts against *S. aureus*.

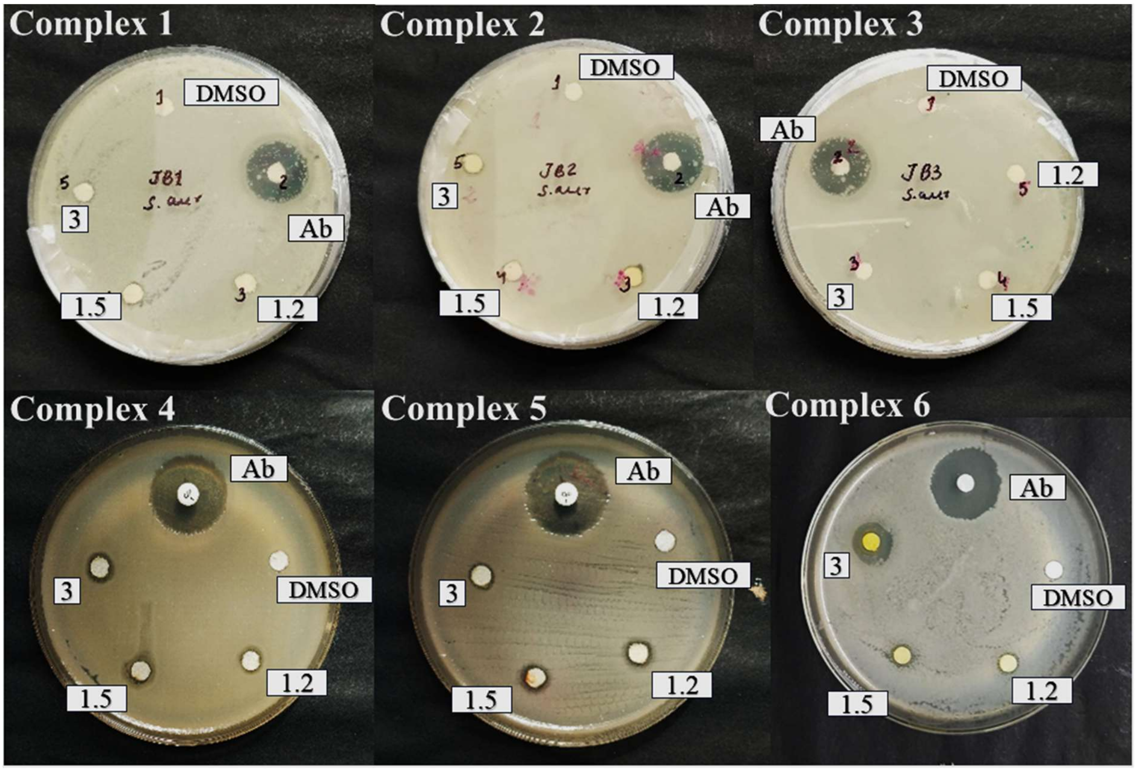

Fig. S42 Zone of inhibition of the complexes (**1**-**6**) against *S. aureus*.

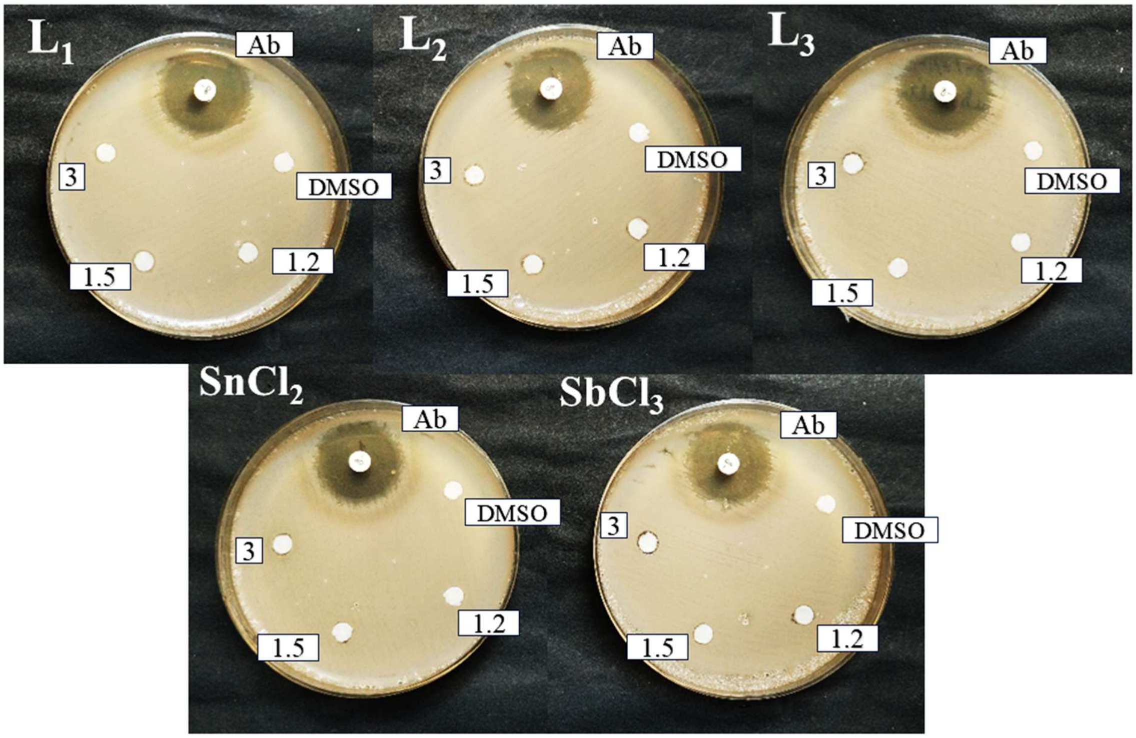

Fig. S43 Zone of inhibition of the ligands (**L_1-L_3**) and metal salts against *E. coli*.

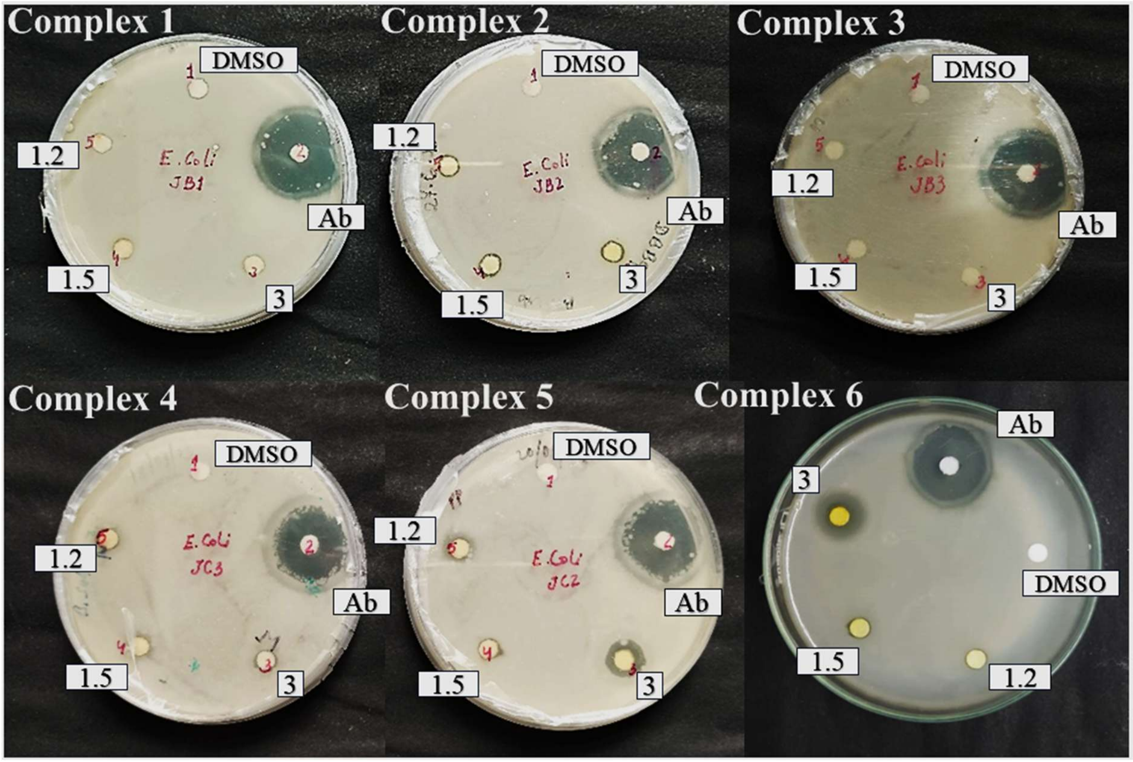

Fig. S44 Zone of inhibition of the complexes (**1-6**) against *E. coli*.

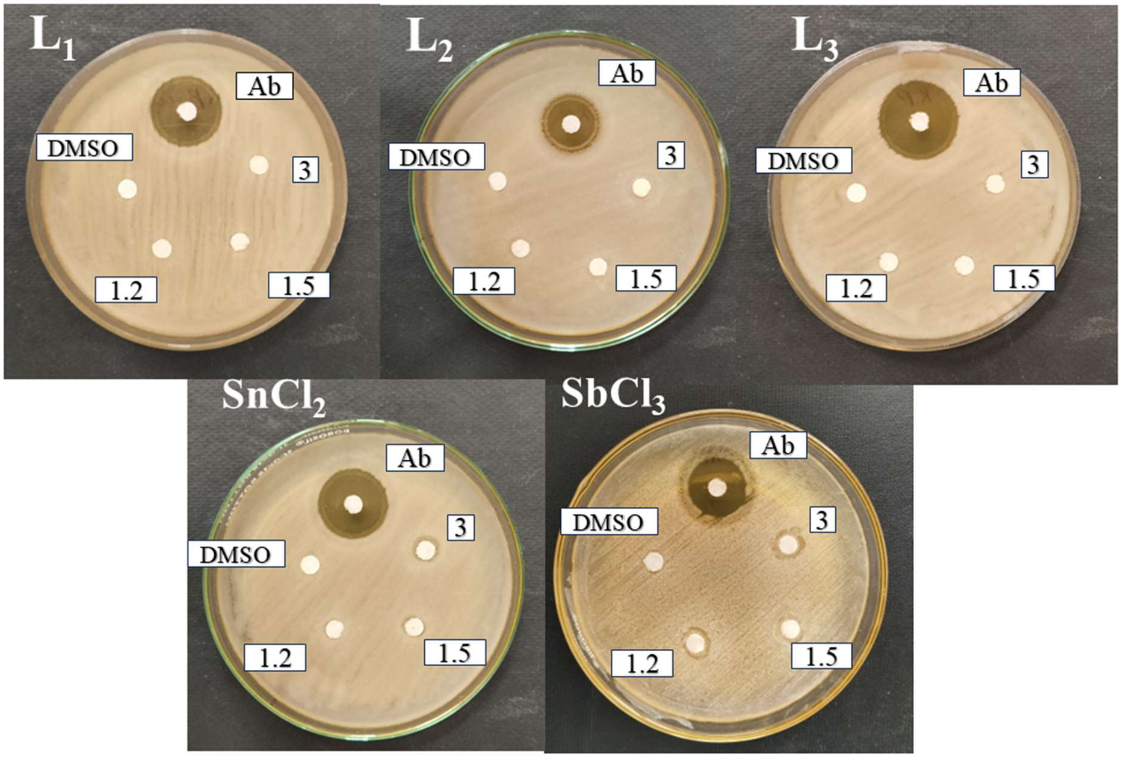

Fig. S45 Zone of inhibition of the ligands (**L_1-L_3**) and metal salts against *P. aeruginosa*.

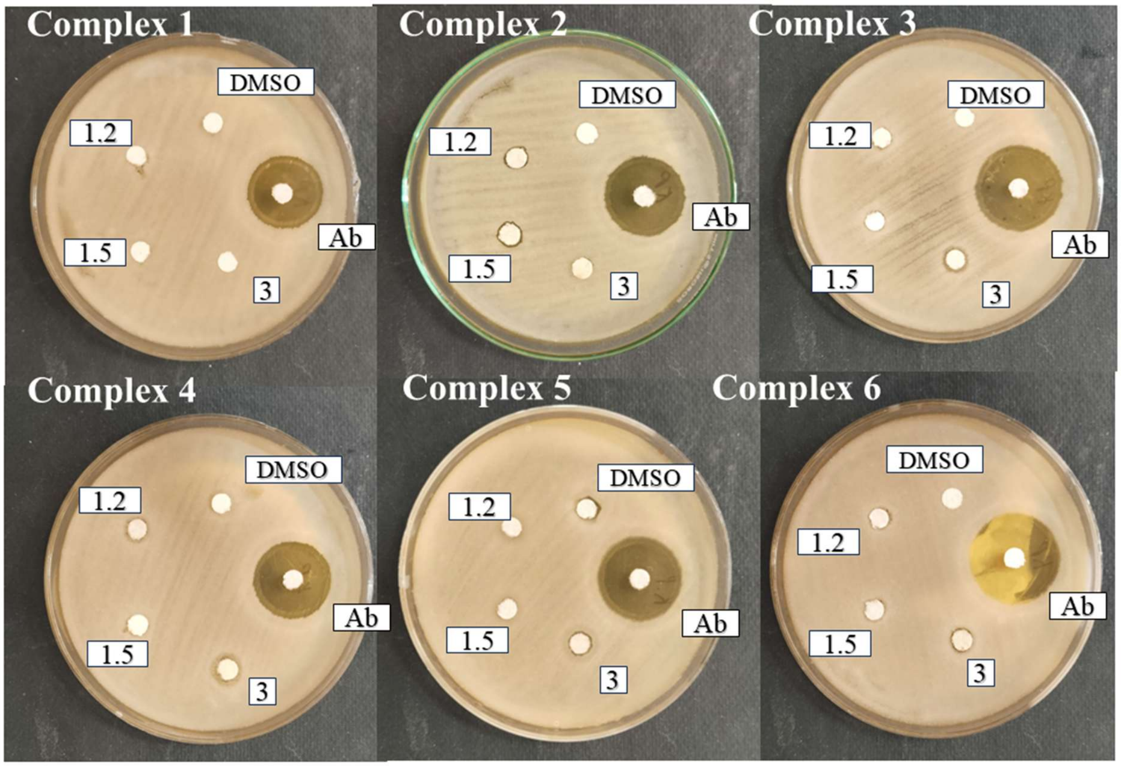

Fig. S46 Zone of inhibition of the complexes (**1**-**6**) against *P. aeruginosa*.

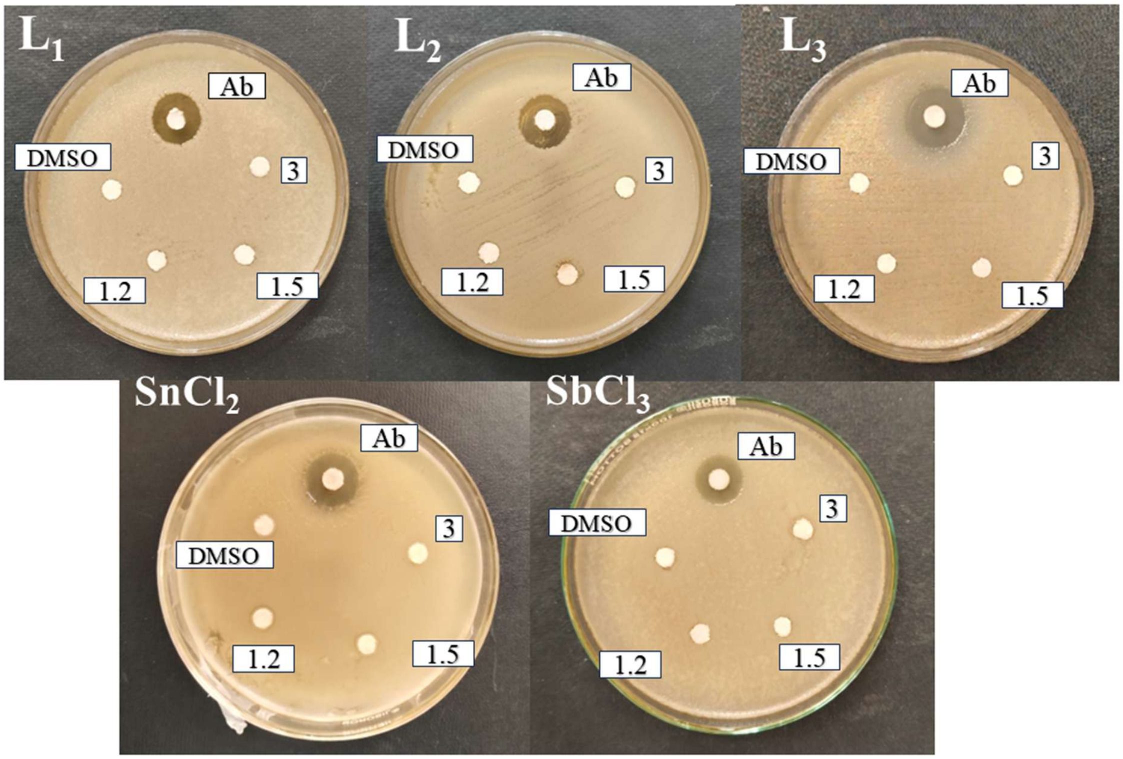

Fig. S47 Zone of inhibition of the ligands (**L_1-L_3**) and metal salts against *K. pneumoniae*.

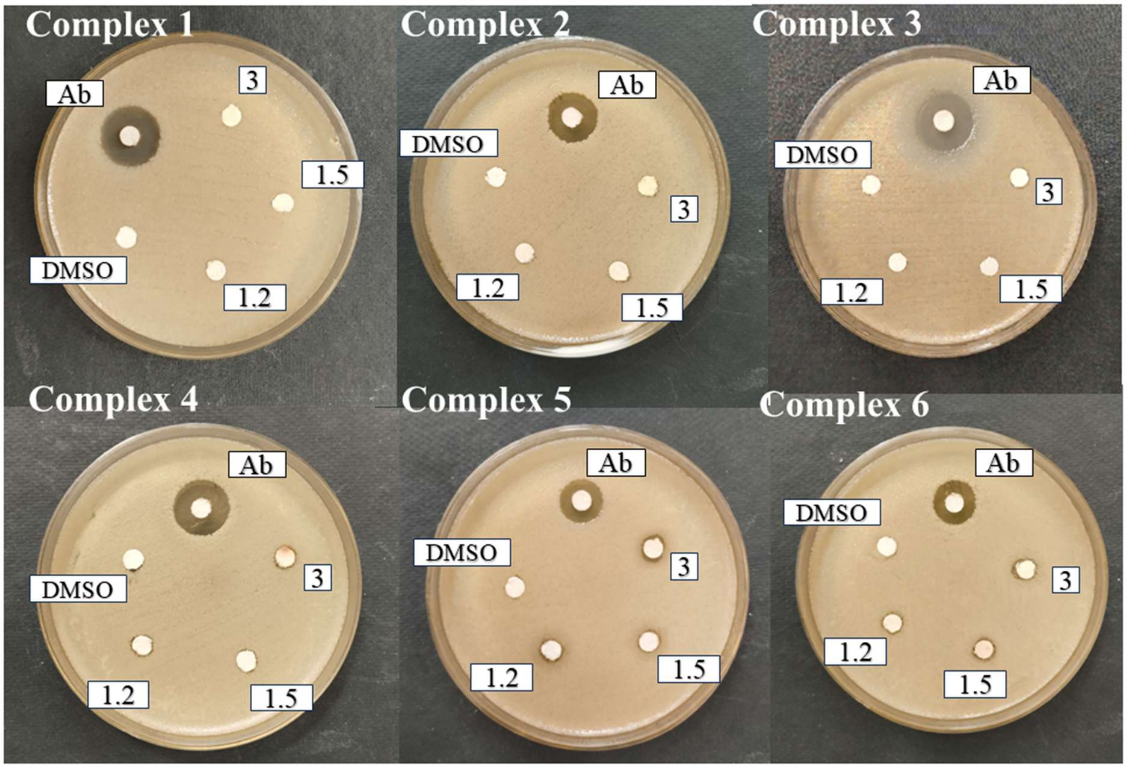

Fig. S48 Zone of inhibition of the complexes (**1-6**) against *K. pneumoniae*.

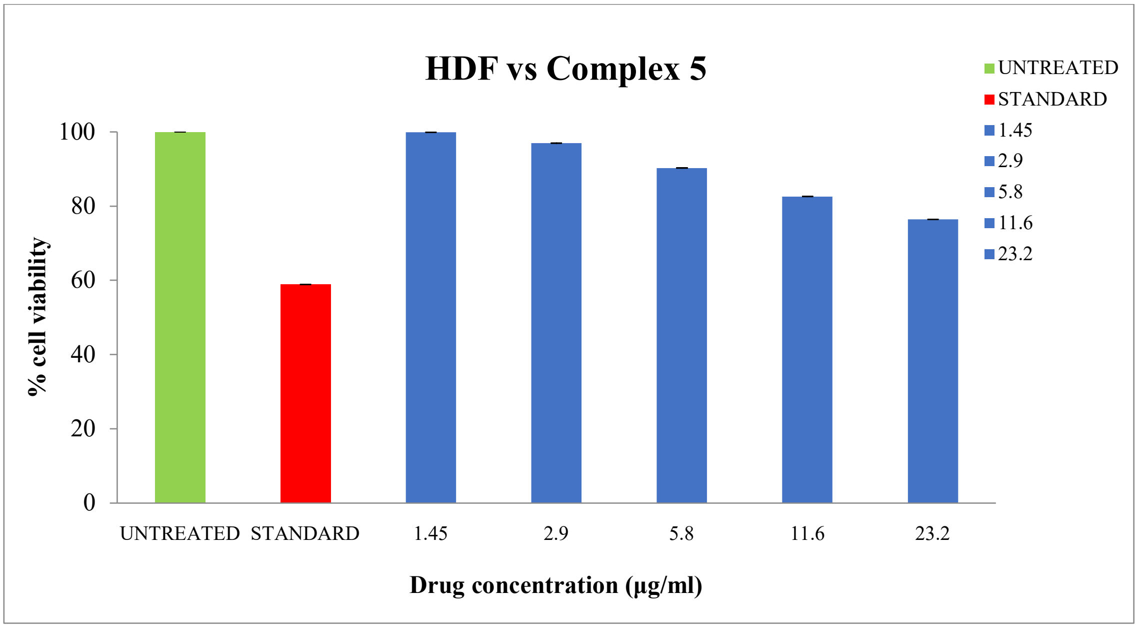

Fig. S49 Mean % cell viability of HDF cells after exposing to complex **5** for 24 hours. [Here, STANDARD indicates the reference drug doxorubicin (positive control) at a concentration of 2.5μM].

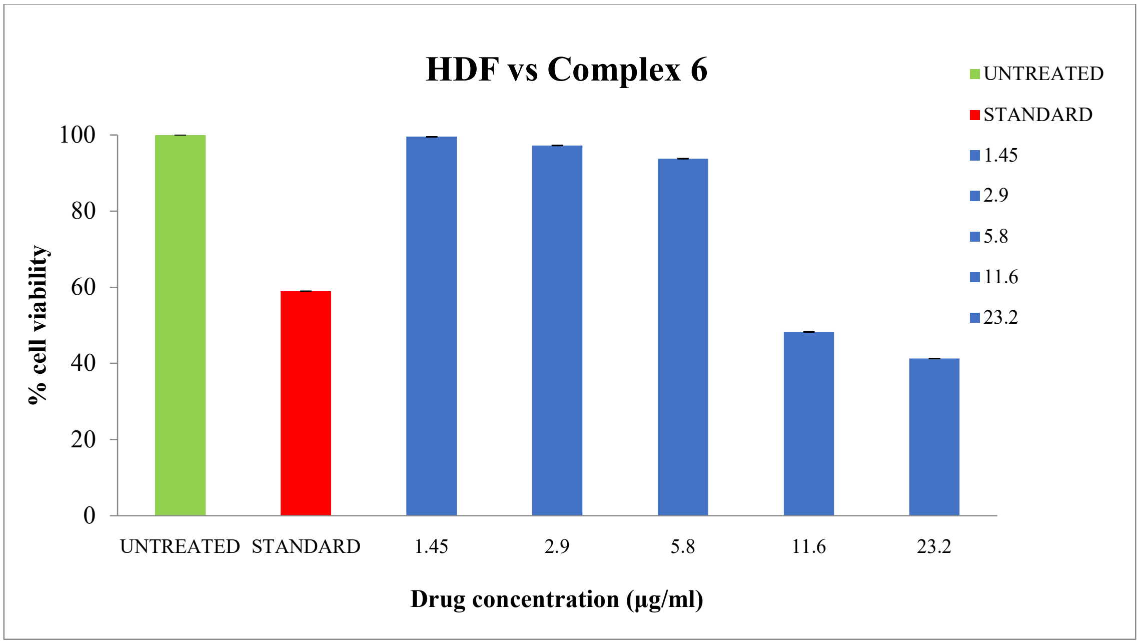

Fig. S50 Mean % cell viability of HDF cells after exposing to complex **6** for 24 hours. [Here, STANDARD indicates the reference drug doxorubicin (positive control) at a concentration of 2.5µM].
